# The First Example of the Friedel–Crafts Cyclization Leading to (10-Hydroxy-9,10-dihydroanthr-9-yl)phosphonium Salts without the Expected Bradsher Dehydration

**DOI:** 10.3390/ijms25031741

**Published:** 2024-02-01

**Authors:** Krzysztof Owsianik, Ewa Różycka-Sokołowska, Marek Koprowski, Marika Turek, Łucja Knopik, Vivek Vivek, Bogdan Dudziński, Piotr Bałczewski

**Affiliations:** 1 Division of Organic Chemistry, Centre of Molecular and Macromolecular Studies, Polish Academy of Sciences, Sienkiewicza 112, 90-363 Łódź, Poland; marek.koprowski@cbmm.lodz.pl (M.K.); lucja.knopik@cbmm.lodz.pl (Ł.K.); vivek@cbmm.lodz.pl (V.V.); bogdan.dudzinski@cbmm.lodz.pl (B.D.); 2 Institute of Chemistry, Faculty of Science and Technology, Jan Długosz University in Częstochowa, Armii Krajowej 13/15, 42-201 Częstochowa, Poland; e.sokolowska@ujd.edu.pl (E.R.-S.); marika.turek93@gmail.com (M.T.); 3 The Bio-Med-Chem Doctoral School of the University of Łódź and Łódź Institutes of the Polish Academy of Sciences, University of Łódź, Matejki 21/23, 90-237 Łódź, Poland

**Keywords:** Friedel–Crafts cyclization, Bradsher dehydration, anthrol, phosphonium salt, phosphine

## Abstract

The reaction of (*ortho-*acetalaryl)arylmethanols with various phosphines PR^1^R^2^R^3^ (R^1^ = R^2^ = R^3^ = Ph; R^1^ = R^2^ = Ph, R^3^ = Me and R^1^ = R^2^ = Me, R^3^ = Ph) under acidic conditions (e.g., HCl, HBF_4_, TsOH) unexpectedly led to the formation of (10-hydroxy-9,10-dihydroanthr-9-yl)phosphonium salts instead of the corresponding anthryl phosphonium salts. The cyclization occurred according to the Friedel–Crafts mechanism but without the usually observed Bradsher dehydration, giving cyclic products in the form of *cis/trans* isomers and their conformers. In case of electron-rich and less-hindered dimethylphenylphosphine, all four stereoisomers were recorded in ^31^P{^1^H} NMR spectra, while for the other phosphines, only the two most stable *cis*/*trans* stereoisomers were detected. This study was supported by DFT and NCI calculations in combination with FT-IR analysis.

## 1. Introduction

Organic electronics and optoelectronics are relatively new fields of basic knowledge and technology that have become a subject of interest to chemists, physicists and process engineers [1,2,3,4]. Therefore, the search for organic fluorescent and semiconducting materials for the construction of new-generation electronic devices, such as organic light-emitting diodes (OLEDs) [5], organic field-effect transistors (OFETs) [6], organic solar cells (OPVs) [7], organic solar concentrators (OSCs) [8], organic lasers [9], etc., has drawn the attention of numerous multidisciplinary joint laboratories. Among the aromatic hydrocarbons, linearly fused acenes are being considered as key organic compounds for achieving these goals [10]. Anthracene and its derivatives are particularly attractive due to their high thermal stability, relatively good solubility, low price and blue photoluminescent and electroluminescent properties [11,12]. Many blue-light-emitting materials with an anthracene core structure have been developed; however, deep blue is still in demand due to the lack of electrically and photochemically stable light-emitting materials [13].

**Scheme 1:**
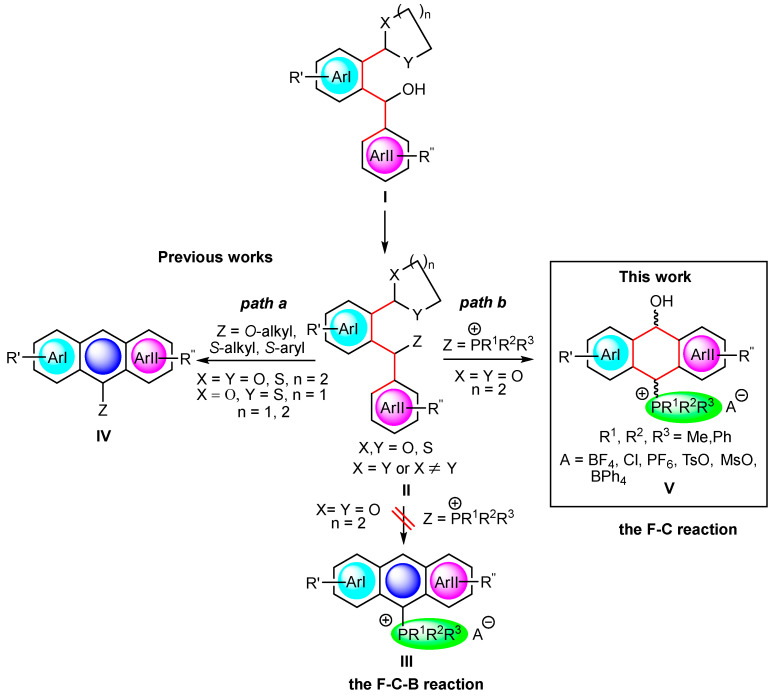
A diagram showing the potential for the synthesis of multiply substituted Z-acenes **IV** (Z = OR, SR; previous works) and dihydroanthrols **V** (Z = P^+^R^1^R^2^R^3^; this work) from diarylmethanols **I**.

Earlier, we elaborated a new approach to mono-hetero (Z = OR, SR)-substituted acenes **IV** via the *hetero-*Friedel–Crafts–Bradsher (F-C-B) cyclization of *ortho*-*O,O*-acetals, *S,S*-dithioacetals and *O,S*-thioacetals **II** obtained from diarylmethanols **I** [14,15,16,17]. In this reaction, a new benzene ring, fused to two other (hetero)aromatic moieties, ArI and ArII, is formed to give acenes **IV** (Scheme 1, path a). A major advantage of this approach over others known in the literature is the possibility of introducing a large number of substituents of a different electron character, which rapidly change the photophysical properties of the obtained acenes.

In addition to the unique multiple substitution, in this study, we planned to definitely increase the electron donor–acceptor properties of such acenes by introducing strongly electron-withdrawing phosphonium groups (P^+^R^1^R^2^R^3^) in order to obtain favorable absorption and fluorescence properties [18,19,20]. Thus, following the hetero-F-C-B strategy, we planned to synthesize anthryl phosphonium salts **III** via intermediate (*ortho-*acetalaryl)arylmethylphosphonium salts **II** (Z = P^+^R^1^R^2^R^3^). However, the change in the nature of the non-ionic Z substituents (Z = OR, SR) to strongly electron-withdrawing and bulky, ionic ones (Z = P^+^R^1^R^2^R^3^) proved to be a challenge and resulted in the unexpected formation of non-aromatic products **V**, which are hitherto unknown derivatives of 9,10-dihydro-9-anthrols (Scheme 1, path b). The reaction proceeded via the Friedel–Crafts mechanism without the subsequent and usually observed Bradsher dehydration leading to aromatic species. Advantageously, the synthesis of the latter was a one-pot reaction bypassing the isolation of intermediate phosphonium salts **II** occurring through the direct treatment of diarylmethanols **I** with various acids (HBF_4_, MeSO_3_H, HCl, TsOH) in the presence of triphenyl-, diphenylmethyl- or dimethylphenylphosphine (Scheme 1).

## 2. Results and Discussion

**Scheme 2:**
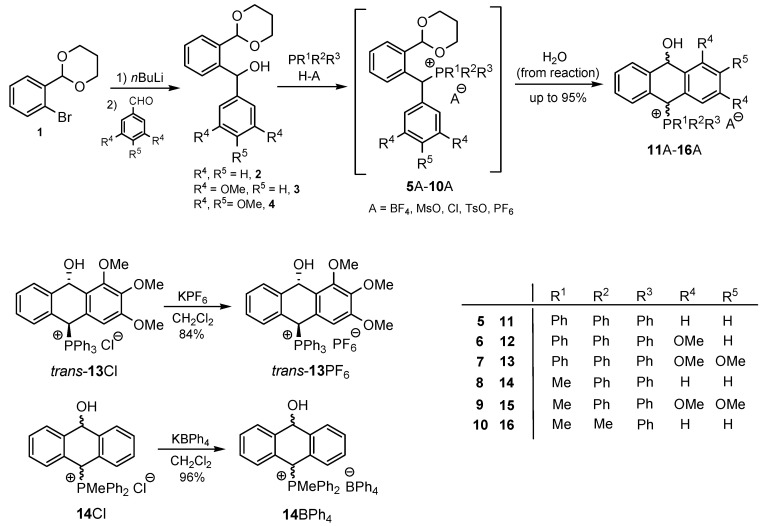
Synthesis of phosphonium salts **11**A–**16**A with different anions.

*Synthesis:* Of the many routes described for the synthesis of phosphonium salts, few relate to those with a specific diarylmethyl substituent [21,22,23]. In this study, we focused on the synthesis of phosphonium salts using diarylmethanols **I** and phosphines as the starting materials under acidic conditions [22]. This approach involving the formal OH/P^+^R^1^R^2^R^3^ conversion seemed to be the most promising, since in the literature, unsubstituted benzyl and dibenzyl alcohols were converted in this way. However, unsubstituted and multiply substituted dibenzyl alcohols and the corresponding phosphonium salts, containing an acid labile *ortho*-acetal function, which enabled a subsequent Friedel–Crafts–Bradsher reaction, had not been investigated so far and required a modified synthesis protocol (Scheme 2).

Thus, diarylmethanols **2**–**4** were first obtained by treating the protected bromobenzaldehyde **1** with *n*-butyllithium followed by other multiply substituted benzaldehydes. The ^1^H-NMR spectra of these diarylmethanols showed characteristic signals confirming their structure. For instance, in the spectrum of diarylmethanol **2**, broad singlets at 3.82 ppm from the OH group and at 5.69 ppm from the 1,3-dioxane CH proton and a characteristic doublet at 6.41 ppm (*J* = 3.3 Hz) due to the C*H*OH proton were observed. Then, the obtained alcohols **2**–**4** were added dropwise at room temperature to the mixture of the acid HA and phosphine solution and stirred for about 1 h. This strategy gave better results and differed from that described in the literature for the synthesis of phosphonium salts [22]. The reverse addition of acid to the diarylmethanol/phosphine solution resulted in the formation of an impure product. The mixture of alcohol and phosphine in the presence of acid HA spontaneously cyclized to give products **11**A–**16**A (A = anion) due to the presence of the *ortho*-acetal function, which, under acidic conditions, enabled the Friedel–Crafts (F-C) cyclization. The products were not the expected anthracenes **III**, as was the case during the previously described intramolecular *hetero*-Friedel–Crafts–Bradsher reactions [15,16,17], but 9,10-dihydroanthrols with a non-aromatic middle ring containing both 9-hydroxyl and 10-phosphonium groups. The subsequent Bradsher dehydration is usually the next step after the F-C reaction leading to the aromatic system, but not in this case [24]. The structures of the unexpected compounds were confirmed by 1D and 2D NMR, HRMS, IR and UV spectra. They were formed as mixtures of stereoisomers resonating in the ^31^P{^1^H} NMR spectrum in the 17–23 ppm range. Interestingly, the *trans/cis* isomeric ratio varied depending on the phosphine used. In case of bulky triphenylphosphine, this ratio was even 12.3:1 (*trans*-**13**Cl:*cis*-**13**Cl), while in case of less-hindered diphenylmethylphosphine and dimethylphenylphosphine, it was almost 1.2:1 (*trans*-**14**MsO:*cis*-**14**MsO and *trans*-**16**BF_4_:*cis*-**16**BF_4_). The intermediates of this reaction were most probably acyclic phosphonium salts **5**A–**10**A, as in other *hetero*-F-C-B reactions [14,17], but they were not detected by the ^31^P{^1^H} NMR spectroscopy and were not isolated due to their rapid cyclization under an acidic conditions. The structure of *trans*-**13**Cl was further confirmed by a chloride anion exchange reaction with KPF_6_, leading to *trans*-**13**PF_6_ (Scheme 2). The high reactivity of phosphonium salts **5**A–**10**A under acidic conditions enabled the use of a one-pot procedure to synthetize salts **11**A–**16**A from diarylmethanols **2**–**4**.

*Structure determination:* The ^1^H NMR spectra of **11**A–**16**A showed characteristic signals in the range of 6.39–8.41 ppm due to the PC*H* group and coupling constants of ^2^*J*_PH_ from 7.5 to 11.0 Hz, and they showed signals in the range of 5.03–6.46 ppm and the corresponding coupling constants of ^5^*J*_PH_ from 6.7 to 11.3 Hz due to the C*H*OH proton (Table 1). Relatively large long-range coupling constants ^5^*J*_PH_ are characteristic of cyclic bisallyl systems [25,26]. In the ^13^C{^1^H} NMR spectra, typical signals from the benzyl group in the range of 78.7–81.9 ppm and ^1^*J*_CP_ coupling constants from 55.7 to 65.0 Hz were observed. The *J*_PH_ and *J*_PC_ coupling constants were identified by ^1^H{^31^P} NMR and ^13^C{^1^H,^31^P} NMR measurements. In some cases, a problem with the identification of PC*H* protons resulted from their overlapping with aromatic protons; therefore, their position in the spectrum could be indirectly determined by recording the ^1^H-^13^C HSQC spectra or by changing the solvent, as was conducted in case of *trans*-**13**PF_6_ (DCM/MeOD).

The position of the PCH protons in the ^1^H NMR spectra depended mostly on the type of anion and phosphine used. According to the studies of non-cyclic benzyl and benzhydryl triphenylphosphonium salts in solution, by Ammer et al., the strong deshielding effect for PC*H* protons was due to the high acidity of the C–H bond. The effect was influenced by both the substituents in the aromatic rings and the type of X^−^ anion that formed PC-H---X^−^ hydrogen bonds. The strongest interactions, according to the literature observations, which were carried out for benzhydryl triphenylphosphonium salt, took place for X = Cl (δ_PC*H*_ = 8.25 ppm) in contrast to X = BF_4_ (δ_PC*H*_ = 6.23 ppm in CD_2_Cl_2_) [22]. In this case, the chloride anion, being the smallest one, interacted more strongly with the PCH hydrogen atom. In the rings substituted with methoxy groups of *trans*-**13**Cl and *trans*-**13**BF_4_, the PCH protons resonated at 8.41 ppm and 8.15 ppm (in CD_2_Cl_2_), respectively. A full overview of the shielding/deshielding effects for **11**A–**16**A is presented in Table 1. The highest differences in the PCH chemical shifts were observed for the group of cyclic diphenylmethylphosphonium salts **14**, between the weakly complexing and large BPh_4_^−^ anions in *trans*-**14**BPh_4_/*cis*-**14**BPh_4_ (6.59/6.39 ppm) and the small Cl^−^ anions in *trans*-**14**Cl/*cis*-**14**Cl (8.32/8.09 ppm).

**Scheme 3:**
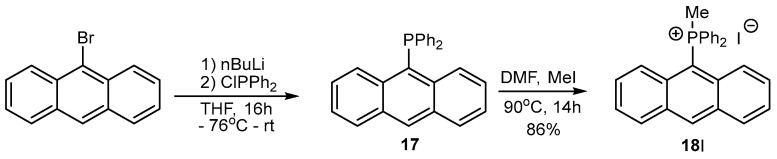
Synthesis of the phosphonium salt **18**I.

The ^31^P NMR spectra of **11**A–**16**A were within the range of 17–23 ppm. To provide further confirmation of the non-aromatic character of the middle ring in the products **11**A–**16**A, the fully aromatic phosphonium salt **18**I was prepared by reacting anthryldiphenylphosphine **17** with methyl iodide in DMF and was characterized by ^1^H, ^31^P and ^13^C NMR, UV and HRMS spectra. It is currently the second known isolated phosphonium salt with an anthryl substituent at the phosphorus atom [27]. A comparison of the spectroscopic data for **18**I and **11**A–**16**A showed a lack of characteristic signals due to C*H*OH benzyl protons in the range of 5.09–6.46 ppm in the ^1^H NMR spectrum and a lack of the signal from the *C*HOH carbon atom in the ^13^C{^1^H} NMR spectrum of **18**I. It is noteworthy that in the ^1^H NMR spectrum, a strongly deshielded signal at 9.11 ppm from the C(9)H carbon atom was recorded [28], which was not observed in the spectra of salts **11**A–**16**A. The ^31^P{^1^H} NMR spectrum of **18**I was characterized by a singlet at 17.6 ppm, and the mass spectrum fully corresponded to the anthracene structure (Scheme 3).

**Figure 1:**
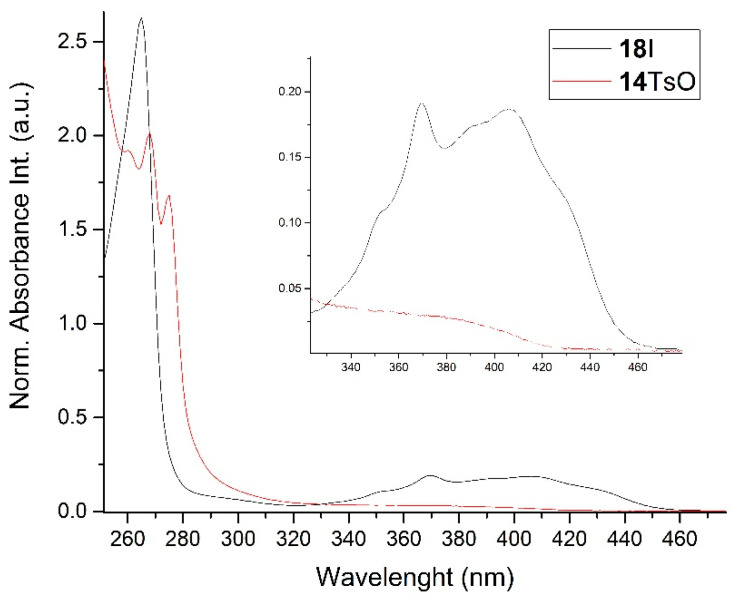
The absorption spectra of 9,10-dihydroanthracene **14**TsO (red line) and anthracene **18**I (black line) in dichloromethane (c = 10^−5^ mol/dm^3^).

Finally, the UV absorption spectra of **14**TsO and **18**I showed different structures of both compounds. The spectrum of **18**I with an anthryl group showed absorption bands at 265, 351, 370, 390, 406 and 432 nm, which were absent in the absorption spectrum of **14**TsO with a 9,10-dihydroanthryl group, where only strong absorption bands at 260, 268 and 275 nm were observed (Figure 1).

The isomeric compounds **11**A–**16**A were spectroscopically characterized as a mixture. Attempts to separate them failed due to their identical *R*_f_ values_._ Moreover, both in the crude reaction mixture and during the purification of triphenylphosphonium salts **12**A and **13**A with a silica gel column, a partial decomposition occurred due to cleavage of the P-C bond and the formation of triphenylphosphine, which was then oxidized to phosphine oxide with air oxygen. Decomposition of the products was partially avoided by using neutral alumina instead of silica gel, which enriched the mixture in one more stable isomer (e.g., *trans*-**13**BF_4_) The introduction of three methoxy substituents onto the side benzene ring in the triphenylphosphonium series increased a steric hindrance and, consequently, significantly prevented the formation of the minor isomer (*trans*-**11**BF_4_ vs *trans*-**13**BF_4_).

**Figure 2:**
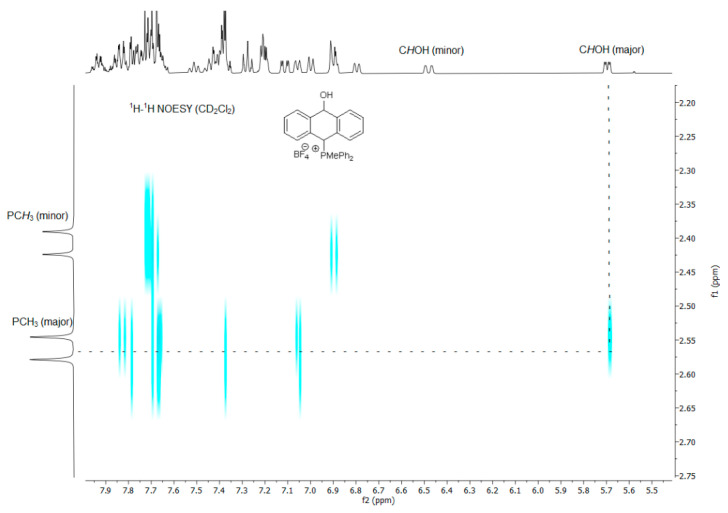
^1^H-^1^H NOESY spectra of the *cis*(minor)/*trans*(major) mixture of the phosphonium salt **14**BF_4_.

**Figure 3:**
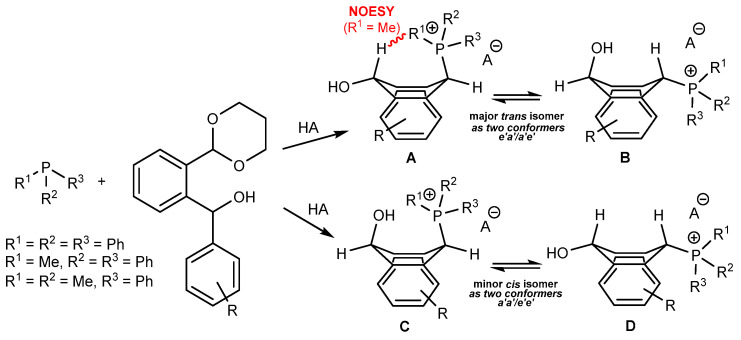
The reaction of phosphines with diarylmethanols under acidic conditions (HA), leading to four stereomeric phosphonium salts **A**–**D**. All four stereoisomers were observed for R^1^ = R^2^ = Me, R^3^ = Ph, while **A** and **C**, as most stable, were only detected for R^1^ = Me, R^2 =^ R^3^ = Ph and for R^1^ = R^2^ = R^3^ = Ph.

^1^H-^1^H NOESY spectra allowed for the determination of the *cis* or *trans* configuration of the major and minor isomer (Figure 2). This experiment, carried out for the salt **14**BF_4_, revealed the proximity of the PCH_3_ and the C*H*OH protons, which indicated that the major isomer with characteristic C*H*OH hydrogen at 5.03–6.46 ppm in the ^1^H NMR spectra was the *trans*-**A** isomer, while the minor isomer with C*H*OH protons at 6.27–6.46 ppm was the *cis* isomer (Figure 3). This proximity was confirmed by DFT calculation, carried out for the *trans*-**A** isomer of **14**Cl.

As we have seen, a larger substituent, such as the triphenylphosphonium group, forced the formation of a *trans*-**A** isomer with a preferred pseudo-axial phosphonium group and a pseudo-equatorial hydroxyl group in ratios of 12.3–2.3:1 for **11**A–**13**A. Replacing the phenyl group with a methyl group in the triphenylphosphonium group reduced this hindrance, and the 1.2–1.4:1 ratio of the two isomers equalized, as observed for compounds **14**A, **15**A and **16**A (Table 1).

The ^31^P{^1^H} NMR temperature experiments (from 25 to 130 °C in DMSO) carried out for the salt **14**BF_4_ revealed two signals due to *cis*/*trans* isomers and no coalescence phenomenon at the tested temperature, which indicated the presence of two configurational isomers, such as **A** or **B** and **C** or **D**.

**Figure 4:**
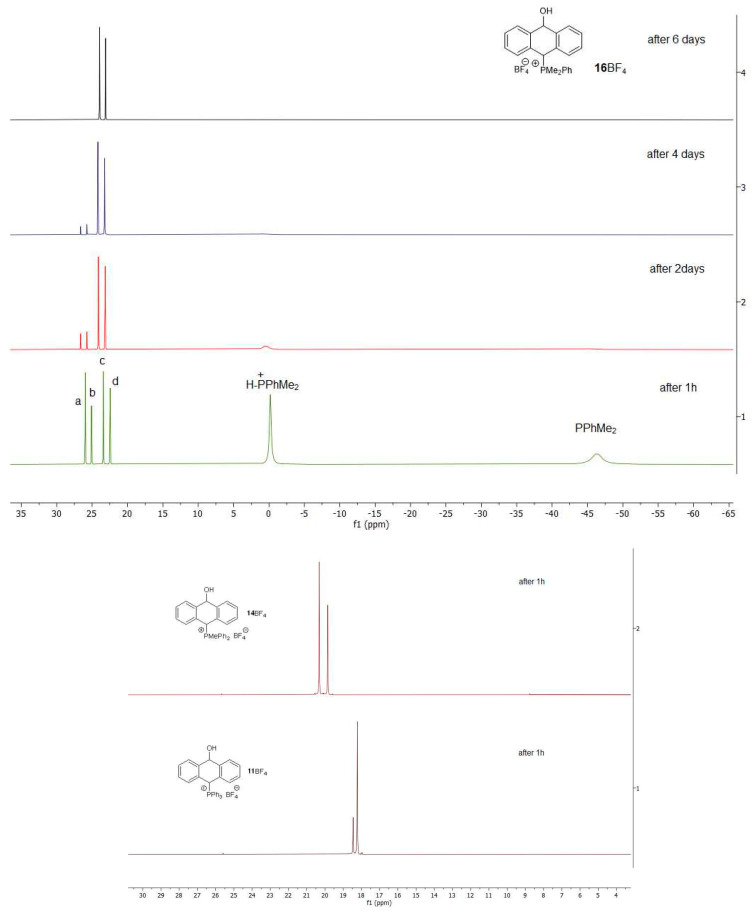
Reaction progress of the alcohol **4** with dimethylphenylphosphine, diphenylmethylphosphine and triphenylphosphine with HBF_4_ in CH_2_Cl_2_ at room temperature monitored with ^31^P{^1^H} NMR. The singlets a–d represent the corresponding products and intermediates **A**–**D**.

**Figure 5:**
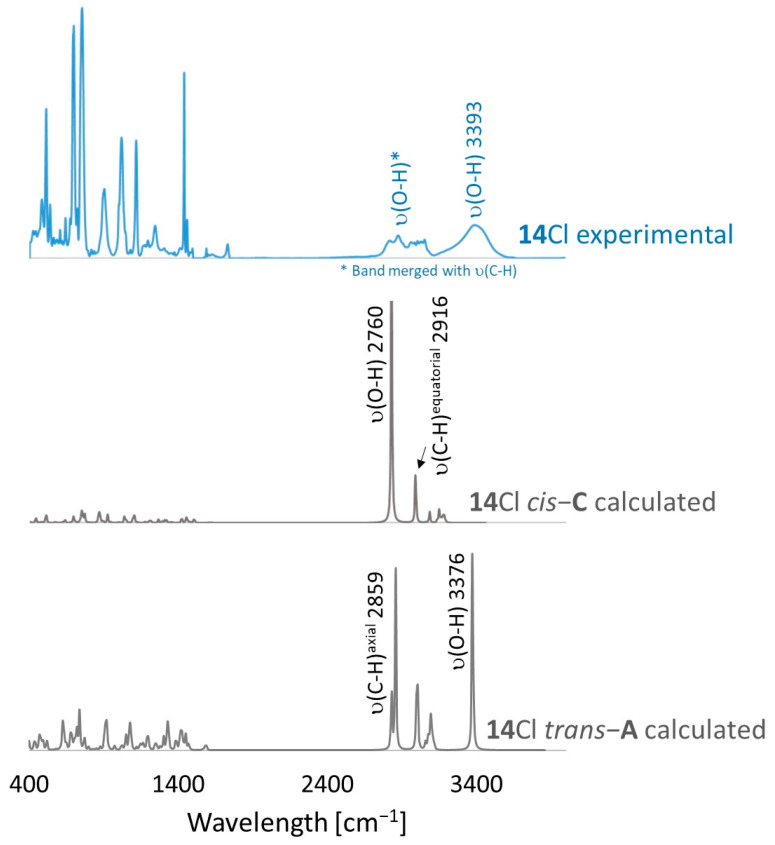
A comparison of experimental (blue line) and theoretical (grey lines) FT-IR spectra for **14**Cl.

Since the ^31^P{^1^H} NMR spectra of the salts with triphenyl- and diphenylmethylphosphine groups did not allow for the observation of all four **A**, **B**, **C** and **D** isomeric forms of the corresponding salts, we increased the electron density on the phosphorus atom, reduced the steric hindrance at this atom by choosing the less-bulky dimethylphenylphosphine and lowered the reaction temperature. Thus, we started our measurements at −15 °C by treating the mixture of dimethylphenylphosphine and HBF_4_ with the alcohol **7**. After increasing the temperature to room temperature, the reaction progress was monitored by ^31^P{^1^H} NMR (Figure 4). After one hour, two strong singlets were observed, which were due the substrates, i.e., dimethylphenylphosphine at −46.2 ppm and protonated phosphine at −0.2 ppm, as well as four singlets, which were due to the four **A–D** isomers of the salt **16**BF_4_. The singlets at 23.4 ppm (*c*) and 22.45 ppm (*d*) corresponded to the *trans-***A** and *cis*-**C** isomers, respectively, while the singlets at 26.0 ppm (*a*) and 25.1 ppm (*b*) were due to their conformers (**B** and **D**). As the reaction progressed, we observed the disappearance of singlets from the substrates and singlets *a* and *b* from the kinetic products. After 6 days, the spectrum showed trace *a* and *b* singlets and dominant *c* and *d* singlets originating from thermodynamically more stable *trans* and *cis* conformational isomers **A** and **C** (see calculations). For a comparison, in the case of the triphenylphosphonium salt **11**BF_4_ and diphenylmethylphosphonium salt **14**BF_4_, no signals from the corresponding starting phosphines and/or their protonated forms were obtained in the ^31^P{^1^H} NMR spectra. Instead, only two singlets in each case, due to the formation of the thermodynamic products in ratios of 3.6:1 and 1.4:1, respectively, were observed after 1 h at room temperature. The electron density on the phosphorus atom was lower in the case of the latter two phosphines than in the case of dimethylphenylphosphine, in which the positive induction effect of the two methyl groups predominated; therefore, protonation of the phosphorus atom by HBF_4_ did not occur in a visible way for these two phosphines. Finally, it can be concluded that there was no equilibration between the thermodynamic stereoisomers **A** and **C** via benzyl carboacation due to the lack of a change in the ratio of *c* and *d* signals during the equilibration process (Figure 3, Figure 4 and Figure 5).

**Figure 6:**
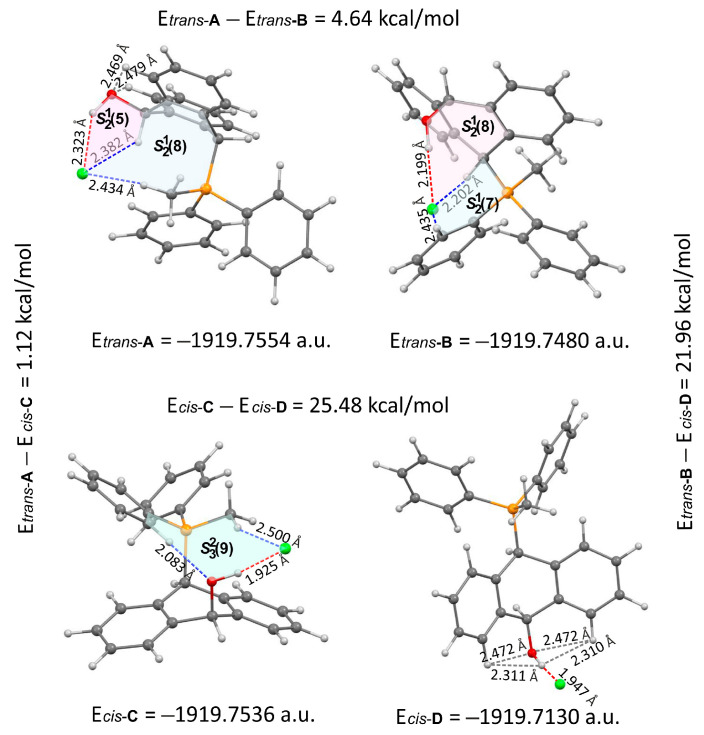
Molecular structures of four stereoisomers **A**–**D** of **14**Cl. The dashed red and blue lines denote O-H∙∙∙Cl^−^ and C-H∙∙∙Cl^−^ hydrogen bonds, and the grey dashed lines denote short non-covalent interactions between atoms of hydroxyl groups and aromatic H atoms.

**Figure 7:**
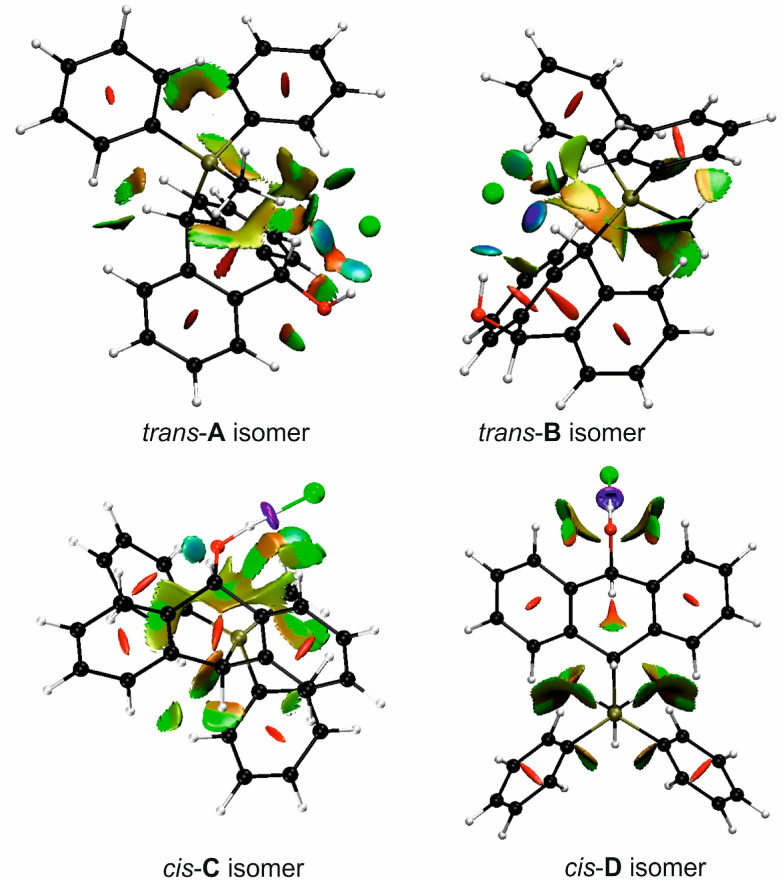
Color-filled reduced density gradient (RDG) isosurfaces depicting non-covalent interactions in four stereoisomers **A**–**D** of **14**Cl.

*FT-IR for **14**Cl in combination with DFT and NCI calculation:* The analysis of the FT-IR spectrum measured for **14**Cl (Table 2, Figure 5 and see Supplementary Materials) proved that the hydroxyl group was covalently bound to the 9-carbon atom of the non-aromatic ring, as evidenced by the presence of υ(C-O) vibrations, both equatorial and axial. By comparing the experimental FT-IR spectrum with the frequencies calculated (DFT-B3LYP 6-31++G(d,p)) for the most stable **A** and **C** isomers of **14**Cl (Figure 3) in the ground state, in the gas phase, it can be concluded that the *trans* stereoisomer **A** predominated in the obtained sample, as evidenced by the position of the vibration of the hydroxyl group (υ(O-H) 3393 cm^−1^), which corresponded to the position of the vibration calculated for the *trans*-**A** isomer (3376 cm^−1^). The analysis of the calculated frequencies indicated that the υ(O-H) vibration in the *cis*-**C** isomer was shifted towards lower wavelengths (calculated value 2760 cm^−1^), and in the *trans*-**A** isomer, it was observed at a higher wavelength (3376 cm^−1^). The coexistence of the *cis* form (in addition to the *trans* form) can be proven by broadening and strengthening the entire υ(C-H) vibration band (2700–2900 cm^−1^), in which the υ(O-H)*_cis_* vibration may be hidden. Importantly, the presence of two bands for both the υ(C-H) and υ(C-O) vibrations proves the co-occurrence of the axial and equatorial forms. In order to explain (1) why the Bradsher dehydration leading to the aromatic system did not occur as the next step after the F-C reaction and (2) thermodynamic stability of the two stereoisomers **A** and **C** present in the product of this reaction, DFT calculations for the four possible **A**–**D** stereoisomers of **14**Cl (Figure 3) in the ground state, in the gas phase, were made. The optimized geometries of the *trans*-**A**, *trans*-**B**, *cis*-**C** and *cis-***D** isomers of **14**Cl are presented in Figure 6. According to the DFT calculations, the *trans-***A** isomer is more thermodynamically stable than its conformer **B** and *cis-***D** isomer by 4.64 and 26.61 kcal/mol, respectively, and only more stable by 1.12 kcal/mol than the *cis-***C** isomer (Figure 7). Thus, **14**Cl exists predominantly in two forms as *trans*-**A** and *cis*-**C** isomers. As illustrated in Figure 6, in the case of each stereoisomer, the Cl^−^ anion is the acceptor in a strong hydrogen bond, with the hydroxyl group acting as the donor. The distance between H and Cl^−^ is shorter than the sum of the van der Waals radii of these atoms by ca. as much as 0.7 Å and 1.0 Å in *trans* and *cis* isomers, respectively. In addition, for the two conformers, **A**, **B** and the isomer **C**, the anion acts as an acceptor for two unconventional but very short C-H∙∙∙Cl^−^ hydrogen bonds (d_H∙∙∙Cl_ < the sum of the van der Waals radii of the H and Cl atoms by ca. 0.5 Å in the case of the *trans*-**A**, 0.8 Å and 0.5 Å in the case of the *trans*-**B** and 0.9 Å and 0.2 Å in the case of the *cis-***C** isomers). In the *trans*-**A** and **B** isomers, the mutual combination of the hydrogen bond of the type O-H∙∙∙Cl^−^ with P-C-H∙∙∙Cl^−^ (in **B**) or O-C-H∙∙∙Cl^−^ (in **A**) hydrogen bonds leads to the formation of rings with descriptors in the form of *S*_2_^1^(5) or *S*_2_^1^(5), respectively. In the *cis*-**C** isomer, a combination of three hydrogen bonds, i.e., one O-H∙∙∙Cl^−^ and two C-H∙∙∙Cl^−^, leads to the formation of a *S*_3_^2^(9) ring. This type of intramolecular ring does not occur only in the highest-energy *cis-***D** isomer, which results from the lack of hydrogen bonds other than O-H∙∙∙Cl^−^. Practically all of the above hydrogen bonds, and especially O-H∙∙∙Cl^−^, are visible on three-dimensional NCI diagrams (Figure 6) as dark or light-blue, disk-shaped isosurfaces, indicating strong attractive interactions. The above analysis of the strongest non-covalent interactions occurring in the **14**Cl isomers allowed us to conclude that the Bradsher dehydration associated with the elimination of the hydroxyl group could not occur due to (1) its participation in strong hydrogen bonds and the motifs formed by them (Figure 6) and, especially, (2) combinations of the many weak, non-covalent van der Waals interactions visible in the two isomers *trans*-**A** and *cis*-**C** as greenish isosurfaces, which prevent the middle ring from flattening to form an aromatic system (Figure 7). In the minor *trans*-**B** and *cis*-**D** isomers, the number of these interactions is much smaller (Figure 7), which causes their interconversion to the more stable isomers *trans*-**A** and *cis*-**C**.

**Scheme 4:**
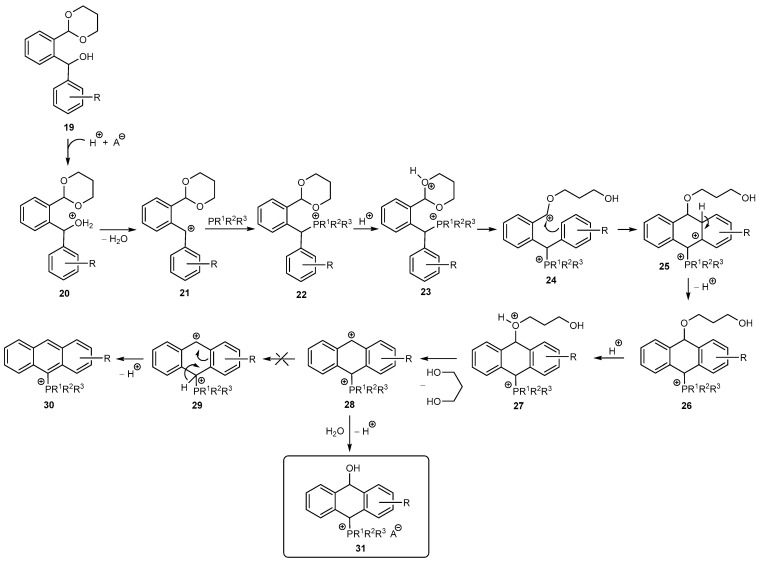
Plausible mechanism for the synthesis of phosphonium salts **31**.

*Reaction mechanism:* The reaction between diarylmethanol **19** and phosphine PR^1^R^2^R^3^ under acidic conditions in the first step led to protonation of the OH group to give **20** followed by the dehydration and formation of the carbocation **21**, which reacted with the phosphine to give the phosphonium salt **22**. However, in the case of electron-rich phosphine PMe_2_Ph, unlike PMe_2_Ph and PPh_3_, the phosphorus atom was also protonated. In this case, signals from both the phosphine and its protonated form were observed in the ^31^P{^1^H} NMR (Figure 4). In contrast, the phosphonium salt **22** was never detected because it underwent a rapid protonation to give **23** followed by opening the acetal ring and the formation of the carbocation **24**. Then, the ring closed according to the Friedel–Crafts reaction to give **25** and aromatization of the benzene ring brought **26**. Next, protonation of the propandiol moiety gave **27**, which easily lost the diol to obtain the carbocation **28**. The ^1^H NMR spectrum of the crude mixture showed signals from free propanediol and no signal from the aldehyde group, indicating the removal of the protecting group. Interestingly, the carbocation **28** did not undergo further aromatization of the middle ring, typically via the Bradsher dehydration, to give the anthracene **30** via the dicationic species **29**, but it unexpectedly reacted with water originating from the dehydration of **20** (in the case of anhydrous acids, e.g., HCl in Et_2_O, HBF_4_^.^Et_2_O) and/or from the aqueous acid solutions (e.g., HPF_6(aq)_, HCl_aq_, *p*-TosH^.^H_2_O) to give (10-hydroxy-9,10-dihydroanthr-9-yl)phosphonium salts **31** (Scheme 4).

**Scheme 5:**
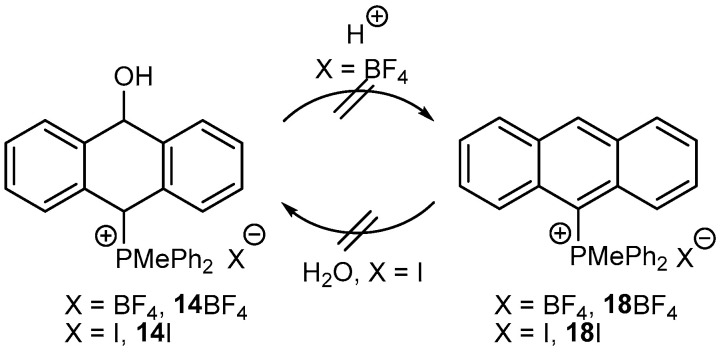
The reactivity study of the anthrol **14**BF_4_ to acid and the anthracene **18**I to water.

Attempts to dehydrate the isolated anthrols to anthracenes under strongly acidic conditions failed. For instance, **14**BF_4_ did not aromatize with various acids (HCl, H_2_SO_4_) to obtain **18**BF_4_ at room temperature and even at higher temperatures. The reverse reaction, i.e., the addition of water to the activated by a phosphonium group anthracene **18**I to form **14**I, even at higher temperatures, also did not occur (Scheme 5).

## 3. Materials and Methods

Organic solvents were purchased from commercial sources (ChemPur, Piekary Śląskie, Poland; POCh, Gliwice, Poland) and used as-received or dried using standard procedures. Tetrahydrofuran (THF) was purchased from J.T. Baker and purified using a Solvent Purification System (MBraun SPS-800). All reagents were from obtained commercial suppliers (Sigma-Aldrich, Merck–USA, Beijing, China) and used without further purification. An Avance NEO 400 NMR spectrometer, operating at 400.15 MHz for ^1^H, 100.63 MHz for ^13^C, 128.40 MHz for ^31^P, 376.55 MHz for ^19^F and 192.64 MHz for ^11^B, was used, which consisted of a narrow-bore 9.4 Tesla Ascend superconducting magnet and an Avance NEO console from Bruker Corporation (Karlsruhe, Germany) operating with a SampleCase Plus autosampler. The spectrometer was equipped with 5 mm high-resolution dual-channel ^2^H/^1^H(^19^F)/BB i-Probe (Bruker) with a z-gradient coil capable of tuning to nuclei ^19^F/^31^P-199Hg and 17O-109Ag on the BB channel with an automatic tuning and matching system (ATMA). The spectrometer used a BCU-I, controlled by a Bruker Smart Variable Temperature (BSVT) system for the temperature regulation and stabilization. The spectrometer was operated using the TopSpin 4.1 program. High-resolution mass spectrometry (HRMS) measurements were performed using an SQ Detector 2 mass spectrometer (Waters, Milford, MA, USA). Melting points were measured using a Boetius apparatus. Thin-layer chromatography (TLC) was performed on precoated Merck 60 (F254 60, Darmstad, Germany) silica gel plates with a fluorescent indicator with detection by means of UV light at 254 and 360 nm or on aluminum cards (Fluka Chemie AG, Buchs, Switzerland) with a fluorescent indicator with detection by means of UV light at 254 nm. Column chromatography was performed on Merck silica gel (Kieselgel 60, Darmstad, Germany, 230–400 mesh) or using a Pure FlashPrep 850 Chromatography System (Büchi, Flawil, Switzerland). The UV-Vis absorption spectra were recorded in 1 cm cuvettes on a Shimadzu UV-2700 spectrophotometer (Kioto, Japan) using two types of light source: a D2 64604 deuterium lamp and a Wl L6380 halogen lamp (Kioto, Japan, 220–600 nm).

*Fourier Transform Infrared Spectroscopy:* The Fourier transform mid-infrared spectra of the compounds **14**Cl were measured at a 2 cm^−1^ resolution and by 32 scans on a Nicolet-Nexus spectrometer in the region of 4000–400 cm^−1^ using the KBr pellet technique. The data were processed using spectrum software OMNIC 8.2.0.387 (Thermo Fisher Scientific Inc., Waltham, MA, USA). IR frequencies predicted for the **14**Cl *trans-***A** and *cis-***C** isomers were compared with experimental frequencies measured for the synthesized **14**Cl compounds. A precomputed vibrational scaling factor of 0.966 was used.

*DFT and NCI calculations.* The molecular structures of the four stereoisomers **A**–**D** of **14**Cl^−^ were calculated by the DFT method using a gradient-corrected three-parameter hybrid functional (B3LYP) with the 6-31++G(d,p) basis set. Full-geometry optimizations of the compounds in the gas phase were performed using their starting geometries prepared manually. The above computations were performed using the GAUSSIAN09 quantum chemistry package. In order to check the structural optimizations, the calculated vibrational frequencies of the compounds were used (no imaginary frequencies). The IR frequencies predicted for the **4**Cl *trans-***A** and *cis-***C** isomers were compared with the experimental frequencies measured for the synthesized **14**Cl compound. A precomputed vibrational scaling factor of 0.966 was used. The calculations in the framework of the noncovalent interactions (NCI) method were performed with the Multiwfn 3.8 version (A Multifunctional Wavefunction Analyzer) software. This method is based on the relationship between the electron density (ρ) and the reduced density gradient (RDG) and is a very useful tool for the analysis and visualization of different types of non-covalent interactions. The Mercury program was employed to visualize the chemical structures of the investigated isomers, and the VMD (Visual Molecular Dynamics)) 1.9.3. software and Multiwfn 3.8 version software were used for the visualization of their RDG isosurfaces.

### 3.1. General Procedure for Synthesis of o-Acetalaryl(aryl)methanols

2-(2-Bromophenyl)-1,3-dioxane **1** (5 mmol, 1.21 g) was placed in a round-bottom flask and dissolved in dry THF (50 mL) at −78 °C under an argon atmosphere. Next, 2.2 mL of *n*-BuLi (5.5 mmol, 2.5 M in hexane) was added. The resulting mixture was stirred for 15 min under argon, and then aromatic aldehyde (5.5 mmol) was added in dry THF. Stirring was continued for 2 h at −78 °C, and the reaction mixture was warmed to room temperature. Saturated aqueous NH_4_Cl solution was added, and the solvent was evaporated. The residue was diluted with ethyl acetate (3 × 30 mL), washed with water (40 mL) and dried over anhydrous MgSO_4_. After filtration, ethyl acetate was removed in a vacuum, and the crude product **2**, **3** or **4** was purified by column chromatography over silica gel with a mixture of hexane/ethyl acetate (4:1 *v*/*v*) as an eluent.

(2-(1,3-Dioxan-2-yl)phenyl)phenylmethanol (**2**). White solid, m.p: 84–85 °C, yield: 85%. ^1^H NMR (CD_2_Cl_2_) δ 7.71–7.64 (m, 1H, Ar), 7.54–7.33 (m, 7H, Ar), 7.33–7.25 (m, 1H, Ar), 6.41 (d, *J* = 3.3 Hz, 1H, C*H*OH), 5.69 (s, 1H, CHO), 4.29 (ddd, *J* = 11.5, 4.4, 2.9 Hz, 2H, OCH_2_), 3.98 (ddd, *J* = 12.1, 6.1, 2.6 Hz, 2H, OCH_2_), 3.83 (s, 1H, OH), 2.34–2.20 (m, 1H, CH_2_), 1.48 (m, 1H, CH_2_) ppm. ^13^C{^1^H} NMR (CD_2_Cl_2_) δ 143.4, 142.6, 136.2, 129.2, 128.8, 128.2, 127.5, 127.0, 126.6, 101.1, 71.7, 67.6, 67.5, 25.7 ppm. HRMS (EI, 70 eV) *m*/*z* calc. for [M]^+^ C_17_H_18_O_3_ 270.1256, found 270.1257.

(2-(1,3-Dioxan-2-yl)phenyl)(3,5-dimethoxyphenyl)methanol (**3**) [17]. Colorless oil, yield: 85%. ^1^H NMR (CD_2_Cl_2_) δ 7.62 (dd, *J* = 5.6, 3.5 Hz, 1H), 7.35 (dd, *J* = 5.8, 3.4 Hz, 2H), 7.26 (dd, *J* = 5.7, 3.4 Hz, 1H), 6.65 (dd, *J* = 2.4, 0.8 Hz, 2H), 6.44 (dd, *J* = 2.4, 2.4 Hz, 1H), 6.30 (d, *J* = 3.0 Hz, 1H), 5.70 (s, 1H), 4.29 (ddd, *J* = 9.5, 7.4, 3.6 Hz, 2H), 4.05–3.94 (m, 2H), 3.81 (s, 6H), 2.34–2.19 (m, 1H), 1.54–1.44 (m, 1H) ppm. ^13^C{^1^H} NMR (CD_2_Cl_2_) δ 160.8 (C), 145.8 (C), 142.4 (C), 136.1 (C), 129.2 (CH), 128.7 (CH), 127.5 (CH), 126.9 (CH), 104.5 (CH), 101.1 (CH), 98.9 (CH), 71.6 (CH), 67.6 (CH_2_), 67.5 (CH_2_), 55.3 (CH_3_), 25.7 (CH_2_) ppm. HRMS (EI, 70 eV) *m*/*z* calc. for [M]^+^ C_19_H_22_O_5_ 330.1467, found 330.1470.

(2-(1,3-Dioxan-2-yl)phenyl)(3,4,5-trimethoxyphenyl)methanol (**4**) [17]. Colorless oil, yield: 88%. ^1^H NMR (C_6_D_6_) δ 7.85–7.75 (m, 1H), 7.48–7.36 (m, 1H), 7.20–7.11 (m, 1H), 7.04 (s, 2H), 6.63 (d, *J* = 3.4 Hz, 1H), 5.63 (s, 1H), 4.02–3.74 (m, 2H), 3.95 (s, 3H), 3.54–3.27 (m, 2H), 3.46 (s, 6H), 2.00–1.74 (m, 1H), 0.72–0.59 (m, 1H) ppm. ^13^C{^1^H} NMR (C_6_D_6_) δ 154.8, 144.3, 139.8, 138.9, 137.5, 130.2, 130.3, 128.5, 128.3, 32.4, 105.2, 102.6, 72.8, 68.1, 68.0, 61.2, 56.4, 26.3 ppm. HRMS (EI, 70 eV) *m*/*z* calc. for [M]^+^ C_20_H_24_O_6_ 360.1573, found 360.1571.

### 3.2. General Procedures for Synthesis of (10-hydroxy-9,10-dihydroanthr-9-yl)phosphonium Salts

#### 3.2.1. Synthesis of Phosphonium Salts **12**A and **13**A

To a solution of triphenyl phosphine (1 mmol) and dry acid (HBF_4_·Et_2_O/MeSO_3_H/HCl in Et_2_O) (1 mmol) in DCM (3 mL), a solution of *o*-acetalaryl(aryl)methanol **3** or **4** (1 mmol) in 3 mL of DCM was added at room temperature. The mixture was stirred under an argon atmosphere for 1 h, and then the solvent was removed with a vacuum pump. The crude products **12** and **13** were purified by flash chromatography over neutral Al_2_O_3_ with a mixture of ethyl acetate/MeOH (1:5 *v*/*v*).

*Trans*-(10-hydroxy-2,4-dimethoxy-9,10-dihydroanthr-9-yl)triphenylphosphonium tetrafluoroborate (*trans*-**12**BF_4_). Yellowish amorphous solid, yield: 54%. ^1^H NMR (200 MHz, CDCl_3_) δ 7.90–7.71 (m, 4H), 7.70–7.53 (m, 11H), 7.47 (dd, *J* = 11.0, 3.6 Hz, 1H, CHP), 7.36–7.16 (m, 2H), 6.87 (d, *J* = 7.5 Hz, 1H), 6.73 (d, *J* = 7.8 Hz, 1H), 6.35 (d, *J* = 2.3 Hz, 1H), 6.29 (d, *J* = 2.3 Hz, 2H), 5.03 (d, *J* = 7.4 Hz, 1H, CHOH), 3.73 (s, 6H) ppm. ^13^C{^1^H} NMR (CD_2_Cl_2_) δ 161.2 (C), 143.4 (d, *J* = 4.9 Hz, C), 141.9 (d, *J* = 6.2 Hz, C), 135.9 (d, *J* = 3.1 Hz, CH), 134.5 (d, *J* = 9.3 Hz, CH), 133.8 (d, *J* = 16.9 Hz, CH), 130.5 (d, *J* = 12.2 Hz, CH), 129.2 (d, *J* = 8.7 Hz, CH), 123.1 (d, *J* = 3.1 Hz, CH), 122.6 (d, *J* = 3.1 Hz, CH), 115.7 (d, *J* = 81.3 Hz, C), 104.9 (CH), 88.6 (CHOH), 81.9 (d, *J* = 58.2 Hz, PCH), 55.4 (OCH_3_) ppm. ^31^P{^1^H} NMR (81 MHz, CDCl_3_) δ 18.5 ppm. HRMS (ESI+) *m*/*z* calc. for [M]^+^ C_34_H_30_O_3_P^+^ 517.1927, found 517.1925.

*Trans*-(10-hydroxy-2,3,4-trimethoxy-9,10-dihydroanthr-9-yl)triphenylphosphonium tetrafluoroborate (*trans*-**13**BF_4_). Yellowish amorphous solid, yield: 57%. ^1^H NMR (500 MHz, Acetone-*d*_6_) δ 8.15 (dd, *J* = 10.1, 2.3 Hz, 1H, CHP), 7.97–7.88 (m, 3H), 7.86-7.61 (m, 13H), 7.40 (dd, *J* = 7.7, 7.7 Hz, 1H), 7.26 (dd, *J* = 7.7, 7.7 Hz, 1H), 7.08 (d, *J* = 7.7 Hz, 1H), 6.79 (d, *J* = 7.7 Hz, 1H), 6.66 (s, 2H), 5.49 (dd, *J* = 6.7, 2.3 Hz, 1H, CHOH), 3.72 (s, 6H, OMe), 3.66 (s, 3H, OMe) ppm. ^13^C{^1^H} NMR (50 MHz, CDCl_3_) δ 152.2 (C), 142.1 (d, *J* = 4.9 Hz, C), 134.4 (d, *J* = 3.0 Hz), 134.2 (d, *J* = 6.5 Hz), 133.1 (d, *J* = 9.2 Hz, CH), 129.2 (d, *J* = 12.3 Hz, CH), 129.8 (CH), 127.7 (CH), 121.6, 114.7 (d, *J* = 81.1 Hz, C), 102.6 (CH), 87.6 (CH), 80.1 (d, *J* = 57.1 Hz, CHP), 59.4 (OCH_3_), 55.0 (OCH_3_) ppm. ^31^P{^1^H} NMR (Acetone-*d*_6_) δ 18.6 ppm. ^19^F{^1^H} NMR (Acetone-*d*_6_) δ −150.70 ppm. HRMS (ESI+) *m*/*z* calc. for [M]^+^ C_35_H_30_O_3_P 529.1933, found 529.1927.

*Trans*-(10-hydroxy-2,3,4-trimethoxy-9,10-dihydroanthr-9-yl)triphenylphosphonium methanesulfonate (*trans*-**13**MsO). Yellow amorphous solid, yield: 38%. ^1^H NMR (CDCl_3_) *δ* 8.20 (d, *J* = 10.2 Hz, 1H, PCH), 7.87–7.82 (m, 2H), 7.76–7.59 (m, 10H), 7.55 (dd, *J* = 7.5, 7.5 Hz, 1H), 7.46 (dd, *J* = 6.5, 6.5 Hz, 1H), 7.39–7.26 (m, 3H), 7.23 (dd, *J* = 7.5, 7.5 Hz, 1H), 6.89 (d, *J* = 7.7 Hz, 1H), 6.81 (d, *J* = 7.7 Hz, 1H), 5.09 (d, *J* = 7.3 Hz, 1H, CHOH), 3.80 (s, 6H, OMe), 3.79 (s, 3H, OMe), 2.71 (s, 3H, SMe) ppm. ^13^C{^1^H} NMR (CDCl_3_) *δ* 153.5, 143.4 (d, *J* = 5.1 Hz), 138.3, 135.6 (d, *J* = 3.0 Hz), 135.3 (d, *J* = 6.5 Hz), 134.6 (d, *J* = 9.3 Hz), 133.7 (d, *J* = 19.5 Hz), 132,1 (*J* = 10 Hz), 132.1 (d, *J* = 2.8 Hz), 131.4 (d, *J* = 2.0 Hz), 130.5 (d, *J* = 12.2 Hz), 128.6 (s, *J* = 12.2 Hz), 128.5 (d, *J* = 7.1 Hz), 123.3, 122.8, 116.3 (d, *J* = 81.0 Hz), 104.2, 88.8 (CHOH), 81.6 (d, *J* = 55.8 Hz, PCH), 39.3 ppm. ^31^P{^1^H} NMR (CDCl_3_) *δ* 17.1 ppm. HRMS (ESI+) *m*/*z* calc. for [M]^+^ C_35_H_30_O_3_P^+^ 529.1933, found 529.1920.

*Trans*-(10-hydroxy-2,3,4-trimethoxy-9,10-dihydroanthr-9-yl)triphenylphosphonium chloride (*trans*-**13**Cl). Orange amorphous solid, yield: 18%. ^1^H NMR (CD_2_Cl_2_) δ 8.41 (dd, *J* = 10.2, 2.6 Hz, 1H, PCH), 7.97–7.88 (m, 3H), 7.80–7.65 (m, 9H), 7.40–7.27 (m, 7H), 6.99 (d, *J* = 7.7 Hz, 1H), 6.77 (dd, *J* = 7.7, 1.7 Hz, 1H), 5.25 (dd, *J* = 7.1, 2.4 Hz, 1H, CHOH), 3.80 (s, 6H), 3.76 (s, 3H) ppm. ^13^C NMR (CD_2_Cl_2_) δ 153.6, 143.4 (d, *J* = 5.1 Hz), 138.4, 137.3 (d, *J* = 11.0 Hz), 135.7 (d, *J* = 3.2 Hz), 135.3 (d, *J* = 6.5 Hz), 134.6 (d, *J* = 9.5 Hz), 133.8, 133.6, 131.4 (d, *J* = 2.0 Hz), 130.7 (d, *J* = 3.6 Hz), 130.4 (d, *J* = 12.2 Hz), 129.1 (d, *J* = 3.2 Hz), 128.8, 128.5 (d, *J* = 6.8 Hz), 128.3, 123.0 (d, *J* = 3.0 Hz), 122.9 (d, *J* = 3.4 Hz), 116.2 (d, *J* = 81.2 Hz), 104.2, 88.8, 81.8 (d, *J* = 55.9 Hz), 60.4, 56.3 ppm. ^31^P{^1^H} NMR (CD_2_Cl_2_) δ 18.3 ppm. HRMS (ESI+) *m*/*z* calc. for [M]^+^ C_35_H_30_O_3_P^+^ 529.1933, found 529.1920.

#### 3.2.2. Synthesis of Phosphonium Salts **11**A and **14**A–**16**A

To a solution of phosphine (1 mmol) and acid (HBF_4_·Et_2_O/MeSO_3_H/HCl_(aq)_/pTsOH·H_2_O/HPF_6(aq)_) (1 mmol) in DCM (5 mL), a solution of *o*-acetalaryl(aryl)methanol **2**, **3** or **4** (1 mmol) in 5 mL of DCM was added at room temperature. The mixture was stirred under an argon atmosphere for 1 h, and then water (5 mL) was added, extracted with DCM (10 mL) and dried over anhydrous MgSO_4_. After separating the drying agent, the resulting solution was concentrated using a vacuum pump. The residue was washed with ethyl acetate twice, and the residual solvent was removed by a vacuum pump.

(10-Hydroxy-9,10-dihydroanthr-9-yl)triphenylphosphonium tetrafluoroborate (**11**BF_4_). Yellowish amorphous solid, yield: 79%. Isomer ratio *trans*/*cis*: 3.6:1. ^1^H NMR (CDCl_3_) δ 7.90–7.80 (m, 6H, major + minor) 7.72–7.57 (m, 23H, major + minor, CHP major), 7.40 (d, ^2^*J*_PH_ = 8.0 Hz, 1H, CHP, minor), 7.37–7.24 (m, 6H, major + minor), 7.21 (dd, *J* = 7.5, 7.5 Hz, 2H, major), 7.18–7.11 (m, 2H, major), 7.06 (dd, *J* = 7.7, 7.7 Hz, 2H, minor), 7.00 (d, *J* = 7.6 Hz, 1H, minor), 6.87 (d, ^3^*J*_HH_ = 7.6 Hz, 1H, major), 6.75 (d, ^3^*J*_HH_ = 7.7 Hz, 1H, major), 6.55 (d, *J*_HH_ = 7.8 Hz, 1H, minor), 6.46 (d, *J*_HH_ = 7.5 Hz, 2H, minor), 6.36 (dd, ^5^*J*_PH_ = 11.3, ^5^*J*_HH_ = 2.3 Hz, 1H, CHOH, minor), 5.15 (dd, ^5^*J*_PH_ = 7.5, ^5^*J*_HH_ = 2.3 Hz, 1H, CHOH, major) ppm. ^13^C{^1^H} NMR (CDCl_3_) δ 143.5 (d, *J* = 5.0 Hz, C), 141.6 (d, J = 5.0 Hz, C), 139.4 (d, *J* = 6.1 Hz, C), 135.8 (d, *J* = 3.2 Hz), 134.7 (d, *J* = 9.4 Hz), 134.5 (d, *J* = 9.5 Hz), 131.2 (C), 130.8, 130.6 (d, *J* = 8.7 Hz), 130.5 (d, *J* = 12.1 Hz), 129.2 (d, *J* = 3.1 Hz), 129.0, 128.8, 128.6, 127.5, 127.3, 123.7, 123.0, 122.9 (d, *J* = 3.0 Hz) 122.3, 115.9 (d, *J* = 81.1 Hz, C), 115.8 (d, *J* = 83.0 Hz, C), 88.7 (CHOH, major), 88.4 (CHOH, minor), 81.6 (d, ^1^*J*_CP_ = 57.0 Hz, CHP, major), 79.9 (d, ^1^*J*_CP_ = 56.0 Hz, CHP, minor) ppm. ^31^P{^1^H} NMR (CDCl_3_) δ 18.5 (minor), 18.2 (major) ppm. ^19^F{^1^H} NMR (CDCl_3_) δ −152.42 (minor), −152.47 (major) ppm. ^11^B{^1^H} NMR (CD_2_Cl_2_) δ −0.98 ppm. HRMS (ESI+) *m*/*z* calc. for [M]^+^ C_32_H_26_OP^+^ 457.1716, found 457.1714.

(10-Hydroxy-9,10-dihydroanthr-9-yl)methyldiphenylphosphonium tetrafluoroborate (**14**BF_4_). Yellow amorphous solid, yield: 95%. Isomer ratio *trans*/*cis*: 1.4:1. ^1^H NMR (CD_2_Cl_2_) δ 7.98–7.86 (m, 2H, major), 7.86–7.60 (m, 18H, major + minor), 7.50 (dd, *J* = 7.6, 7.6 Hz, 1H, minor), 7.47–7.33 (m, 7H, major + minor), 7.28 (dd, *J* = 7.6, 7.6 Hz, 2H, minor), 7.24–7.18 (m, 2H, major), 7.15 (dd, ^2^*J*_PH_ = 9.8, ^5^*J*_HH_ = 2.7 Hz, 1H, CHP, major), 7.05 (d, *J*_HH_ = 7.1 Hz, 1H, major), 6.99 (d, *J*_HH_ = 7.4 Hz, 1H, major), 6.96–6.86 (m, 2H, major + minor), 6.80 (d, *J* = 7.8 Hz, 1H, minor), 6.46 (d, ^5^*J*_PH_ = 11.0 Hz, 1H, CHOH, minor), 5.71 (dd, ^5^*J*_PH_ = 7.1, ^5^*J*_HH_ = 2.7 Hz, 1H, CHOH, major), 3.82 (s, 1H, OH, major), 2.55 (d, *J* = 13.3 Hz, 3H, PMe, major), 2.41 (d, *J* = 13.4 Hz, 3H, PMe, minor), 1.78 (s, 1H, OH, minor). ^13^C{^1^H} NMR (CD_2_Cl_2_) δ 142.8 (d, *J* = 4.9 Hz, C), 140.9 (d, *J* = 6.8 Hz, C), 139.8 (d, *J* = 6.2 Hz, C), 138.0 (C), 135.8 (d, *J* = 3.2 Hz, CH), 135.7 (d, *J* = 3.1 Hz, CH), 135.4 (d, *J* = 3.5 Hz, CH), 133.6 (d, *J* = 9.0 Hz, CH), 133.4 (d, *J* = 9.0 Hz, CH), 133.3 (d, *J* = 9.6 Hz, CH), 133.2 (d, *J* = 9.6 Hz, CH), 132.5 (C), 131.0 (C), 130.7 (d, *J* = 3.4 Hz, CH), 130.5 (CH), 130.4 (d, *J* = 2.9 Hz, CH), 130.3 (CH), 130.3 (CH), 130.2 (CH), 129.2 (d, *J* = 4.1 Hz, CH), 129.1 (CH), 128.8 (CH), 128.1 (CH), 127.2 (CH), 124.0 (C), 123.3 (d, *J* = 2.9 Hz, C), 122.5 (d, *J* = 3.3 Hz, C), 122.0 (C), 117.4 (d, *J* = 81.6 Hz C), 117.3 (d, *J* = 80.6 Hz, C), 115.7 (d, *J* = 61.1 Hz, C), 114.9 (d, *J* = 83.0 Hz, C), 88.8 (CHOH, major), 88.3 (d, *J* = 2.7 Hz CHOH, minor), 80.3 (d, *J* = 59.5 Hz, CHP, major), 78.9 (d, *J* = 65.0 Hz, CHP, minor), 4.9 (d, *J* = 55.0 Hz, PCH_3_, minor), 4.6 (d, *J* = 52.9 Hz, PCH_3_, major) ppm. ^31^P{^1^H} NMR (162 MHz, CD_2_Cl_2_) δ 20.3 (major), 19.9 (minor) ppm. ^19^F{^1^H} NMR (376 MHz, CD_2_Cl_2_) δ −150.69, −150.74 ppm. HRMS (ESI+) *m*/*z* calc. for [M]^+^ C_27_H_24_OP 395.1565, found 395.1580.

(10-hydroxy-9,10-dihydroanthr-9-yl)methyldiphenylphosphonium methanesulfonate (**14**MsO). Yellow amorphous solid, yield: 83%. Isomer ratio *trans*/*cis*: 1.2:1. ^1^H NMR (CD_2_Cl_2_) δ 7.94–7.71 (m, 7H, major + minor), 7.71–7.58 (m, 4H, major + minor), 7.54 (dd, *J* = 8.8, 2.7 Hz, 1H, major, PCH), 7.47 (dd, *J* = 7.6 Hz, 1H, major), 7.41–7.31 (m, 11H), 7.28 (dd, *J* = 8.3, 2.5 Hz, 1H, minor, PCH), 7.26–7.20 (m, 9H, major + minor), 7.17 (dd, *J* = 6.6, 2.0 Hz, 1H, major), 7.13 (d, *J* = 7.6 Hz, 1H, minor), 7.01–6.93 (d, *J* = 13.6 Hz, 1H, major), 6.88 (d, *J* = 7.8 Hz, 1H, minor), 6.85–6.79 (m, 2H, major + minor), 6.42 (dd, *J* = 11.0, 2.5 Hz, C*H*OH, 1H, minor), 5.64 (dd, *J* = 7.2, 2.7 Hz, 1H, C*H*OH, major), 2.69 (d, J = 13.6 Hz, 3H, PMe, major), 2.69 (s, 6H, SMe, major + minor)), 2.57 (d, *J* = 13.6 Hz, 3H, minor) ppm. ^13^C NMR (101 MHz, CD_2_Cl_2_) δ 142.9 (d, *J* = 4.8 Hz), 141.0 (d, *J* = 6.8 Hz), 140.1 (d, *J* = 6.2 Hz), 138.2, 135.5 (d, *J* = 3.2 Hz), 135.4 (d, *J* = 3.1 Hz), 135.2 (dd, *J* = 2.8 Hz), 133.8 (d, *J* = 9.0 Hz), 133.6 (d, *J* = 9.0 Hz), 133.5, 133.4 (d, *J* = 9.4 Hz), 133.0 (d, *J* = 2.4 Hz), 131.5, 130.5 (d, *J* = 3.7 Hz), 130.4, 130.3 (d, *J* = 2.6 Hz), 130.2 (d, *J* = 3.0 Hz), 130.0, 129.1, 129.1 (d, *J* = 2.8 Hz), 128.9, 128.8, 128.7, 128.0, 127.2, 123.9 (d, *J* = 2.6 Hz), 123.1 (d, *J* = 3.0 Hz), 122.7 (d, *J* = 3.2 Hz), 122.1 (d, *J* = 2.5 Hz), 117.7 (d, *J* = 80.7 Hz), 116.2 (d, *J* = 66.1 Hz), 115.4 (d, *J* = 67.6 Hz), 88.5 (CHOH, major), 88.1 (d, *J* = 2.8 Hz, CHOH, minor), 80.4 (d, *J* = 58.4 Hz, PCH, major), 78.9 (d, *J* = 64.1 Hz, PCH, minor), 39.4 (s, SMe), 5.1 (d, *J* = 54.2 Hz), 4.9 (d, *J* = 52.1 Hz). ^31^P{^1^H} NMR (162 MHz, CD_2_Cl_2_) δ 20.7 (s, major), 20.3 (s, minor) ppm. HRMS (ESI+) *m*/*z* calc. for [M]^+^ C_27_H_24_OP^+^ 395.1559, found 395.1562.

(10-hydroxy-9,10-dihydroanthr-9-yl)methyldiphenylphosphonium *para*-toluenesulfonate (**14**TsO). Yellow amorphous solid, yield: 85%. Isomer ratio *trans*/*cis*: 1.3:1. ^1^H NMR (CD_2_Cl_2_) δ 8.09–7.49 (m, 23H, major + minor), 7.48–7.17 (m, 13H, major + minor) 7.14 (d, *J* = 7.7 Hz, 1H, major), 7.10–7.03 (m, 6H, major + minor), 6.92 (d, *J* = 6.4 Hz, 1H, major), 6.83–6.75 (m, 4H, major + minor), 6.27 (dd, ^5^*J*_PH_ = 10.7; ^5^*J*_HH_ =2.9 Hz, 1H, CHOH, minor), 5.59 (dd, ^5^*J*_PH_ = 7.2; ^5^*J*_HH_ =2.7 Hz, 1H, CHOH, major), 2.69 (d, ^2^*J*_PH_ = 13.6 Hz, 3H, major), 2.57 (d, ^2^*J*_PH_ = 13.6 Hz, 3H, minor), 2.32 (s, 6H, major + minor) ppm. ^13^C{^1^H} NMR (CD_2_Cl_2_) δ 145.1, 142.8 (d, *J* = 4.9 Hz), 141.1 (d, *J* = 7.0 Hz), 140.3 (d, *J* = 6.2 Hz), 138.7, 138.4, 135.2 (d, *J* = 3.0 Hz), 135.1 (d, *J* = 3.1 Hz), 135.0 (d, *J* = 3.2 Hz), 134.9 (d, *J* = 3.2 Hz), 133.9 (d, *J* = 9.0 Hz), 133.7 (d, *J* = 4.3 Hz), 133.6 (d, *J* = 4.8 Hz), 133.5 (d, *J* = 9.6 Hz), 133.3 (d, *J* = 2.3 Hz), 131.7, 130.2 (d, *J* = 11.0 Hz), 130.0 (d, *J* = 13.2 Hz), 129.0, 128.89, 128.8, 128.7 (d, *J* = 2.0 Hz), 128.4, 128.0, 127.2, 125.9, 123.6 (d, *J* = 2.4 Hz), 122.9 (d, *J* = 3.2 Hz), 122.1 (d, *J* = 2.7 Hz), 117.8 (d, *J* = 80.6 Hz), 116.5 (d, *J* = 60.3 Hz), 115.7 (d, *J* = 61.7 Hz), 88.3 (CHOH, major), 87.8 (d, ^4^*J*_CP_ = 3.1 Hz, CHOH, minor), 80.4 (d, *J* = 57.9 Hz, CHP, major), 78.7 (d, ^1^*J*_CP_ = 64.0 Hz, CHP, minor), 21.1, 4.8 (d, ^1^*J*_CP_ = 51.7 Hz), 4.8 (d, *J* = 51.9 Hz) ppm. ^31^P{^1^H} NMR (162 MHz, CD_2_Cl_2_) δ 21.0 (major), 20.5 (minor) ppm. HRMS (ESI+) *m*/*z* calc. for [M]^+^ C_27_H_24_OP^+^ 395.1559, found 395.1565.

(10-Hydroxy-9,10-dihydroanthr-9-yl)methyldiphenylphosphonium chloride (**14**Cl). White solid, mp: 78–80 °C, yield: 82%. Isomer ratio *trans*/*cis*: 1.4:1. ^1^H NMR (CD_2_Cl_2_) δ 8.32 (d, *J* = 7.8 Hz, 1H, major), 8.12 (d, *J* = 8.1 Hz, 2H, minor), 8.09 (d, *J* = 8.0 Hz, 1H, minor), 7.97–7.64 (m, 11H, major, minor), 7.64–7.54 (m, 5H, major, minor), 7.43 (dd, *J* = 7.4, 7.4 Hz, 2H, minor), 7.39–7.24 (m, 9H, major, minor), 7.23–7.11 (m, 4H, major, minor), 7.07 (d, *J* = 7.6 Hz, 1H, minor), 6.94–6.86 (m, 2H, major, minor), 6.71 (d, *J* = 7.6 Hz, 2H, minor), 6.34 (d, *J* = 11.2, 1H, minor), 5.46 (d, *J* = 7.6 Hz, 1H, major), 2.98 (d, *J* = 13.8 Hz, 3H, major), 2.87 (d, *J* = 13.9 Hz, 3H, minor) ppm. ^13^C{^1^H} NMR (101 MHz, CD_2_Cl_2_) δ 142.8 (d, *J* = 4.9 Hz, C), 141.0 (d, *J* = 7.0 Hz, C), 140.1 (d, *J* = 6.2 Hz, C), 138.4 (C), 135.2 (d, *J* = 3.1 Hz, CH), 135.1 (d, *J* = 2.9 Hz, CH), 134.9 (d, *J* = 3.5 Hz, CH), 134.9 (d, *J* = 4.1 Hz, CH), 134.0 (d, *J* = 7.5 Hz, CH), 133.4 (d, *J* = 6.3 Hz, CH), 133.8 (d, *J* = 2.9 Hz, CH), 133.7 (d, *J* = 3.3 Hz, CH), 132.0 (d, *J* = 12.6 Hz, CH), 131.9 (d, 5.6 Hz, CH), 130.2 (d, *J* = 3.5 Hz, CH), 130.1 (d, *J* = 12.3 Hz, CH), 130.0 (d, *J* = 12.3 Hz, CH), 129.9 (CH), 129.8 (d, *J* = 2.8 Hz, CH), 128.9 (d, *J* = 2.9 Hz, CH), 128.8 (d, *J* = 2.7 Hz, CH), 128.6 (CH), 128.6 (CH), 128.4 (d, *J* = 4.1 Hz, CH), 128.3 (CH), 127.9 (CH), 127.2 (CH), 123.7 (d, *J* = 2.2 Hz, CH), 123.2 (d, *J* = 3.5 Hz, CH), 122.8 (d, *J* = 3.2 Hz, CH), 122.2 (d, *J* = 2.7 Hz, CH), 117.8 (d, *J* = 79.5 Hz, PC), 117.2 (d, *J* = 53.8 Hz, PC), 115.7 (d, *J* = 81.9 Hz, PC), 88.3 (CHOH), 87.9 (d, *J* = 2.8 Hz, CHOH), 80.9 (d, *J* = 57.1 Hz, PCH), 79.4 (d, *J* = 62.6 Hz, PCH), 6.2 (d, *J* = 50.6 Hz, PCH_3_), 6.1 (d, *J* = 53.0 Hz, PCH_3_) ppm. ^31^P{^1^H} NMR (162 MHz, CD_2_Cl_2_) δ 20.7 (s, major), 20.5 (s, minor) ppm. IR (cm^−1^): 3393, 2876, 1457, 1438, 1114, 1016, 900, 750. HRMS (ESI+) *m*/*z* calc. for [M]^+^ C_27_H_24_OP^+^ 395.1559, found 395.1561.

(10-Hydroxy-9,10-dihydroanthr-9-yl)methyldiphenylphosphonium hexafluorophosphate (**14**PF_6_). White solid, mp: 102–104 °C, yield: 83%. Isomer ratio *trans*/*cis*: 1.4:1. ^1^H NMR (CD_2_Cl_2_) δ 7.97–7.88 (m, 2H, major, minor), 7.88–7.60 (m, 18H, major, minor), 7.51 (dd, J = 7.1, 7.1, 1H, major), 7.44 (dd, *J* = 7.6, 7.6 Hz, 1H, major), 7.41–7.33 (m, 6H, major, minor), 7.28 (dd, *J* = 8.0, 8.0 Hz, 2H, major, minor), 7.26 (d, *J* = 11.1 Hz, 1H, PCH, major), 7.24–7.16 (m, 3H, major, minor), 7.04 (dd, *J* = 9.1, 2.8 Hz, 1H, PCH minor), 7.01 (d, *J* = 8.6 Hz, 2H, major), 6.89 (d, *J* = 7.0 Hz, 2H, minor), 6.76 (d, *J* = 7.9 Hz, 1H, minor), 6.46 (dd, *J* = 11.1, 2.5 Hz, 1H. C*H*(OH), minor), 5.74 (dd, *J* = 7.1, 2.7 Hz, 1H, C*H*(OH), major), 2.57 (d, *J* = 13.3 Hz, 3H, PCH_3_, major), 2.42 (d, *J* = 13.4 Hz, 3H, PCH_3_, minor) ppm. ^13^C{^1^H} NMR (CD_2_Cl_2_) δ 142.7 (d, *J* = 4.7 Hz), 140.8 (d, *J* = 6.7 Hz), 139.7 (d, *J* = 6.3 Hz), 137.9, 135.8 (d, *J* = 3.2 Hz), 135.9 (d, *J* = 3.0 Hz), 135.5 (d, *J* = 3.6 Hz), 135.4 (d, *J* = 3.5 Hz), 133.6 (d, *J* = 8.7 Hz), 133.4 (d, *J* = 9.1 Hz), 133.3 (d, *J* = 9.9 Hz), 133.2 (d, *J* = 9.5 Hz), 132. 4 (d, *J* = 2.6 Hz), 132.0 (d, *J* = 2.8 Hz), 130.8, 130.69 (d, *J* = 19.8 Hz), 130.4 (d, *J* = 2.8 Hz), 130.4 (d, *J* = 16.6 Hz), 130.3 (d, *J* = 17.0 Hz), 129.2 (d, *J* = 13.4 Hz), 129.2 (d, *J* = 3.2 Hz), 128.8, 128.0, 127.2, 124.1 (d, *J* = 2.4 Hz), 123.4 (d, *J* = 3.2 Hz), 122.4 (d, *J* = 3.3 Hz), 122.0, 117.2 (d, *J* = 80.2 Hz, PC), 117.3 (d, *J* = 81.4 Hz, PC), 114.7 (d, *J* = 83.0 Hz, PC), 115.3 (d, *J* = 81.8 Hz, PC), 88.8 (CHOH, major), 88.4 (d, *J* = 2.7 Hz, CHOH, minor), 80.4 (d, *J* = 59.7 Hz, PCH, major), 79.0 (d, *J* = 64.8 Hz, PCH, minor), 5.01 (d, *J* = 55.3 Hz, PCH_3_, minor), 4.8 (d, *J* = 53.0 Hz, PCH_3_, major) ppm. ^31^P{^1^H} NMR (CD_2_Cl_2_) δ 20.2 (s, major), 19.7 (s, minor), −144.4 (hept, *J* = 711.2 Hz) ppm. ^19^F{^1^H} NMR (376 MHz, CD_2_Cl_2_) δ −72.59 (d, *J* = 711.2 Hz) ppm. IR (cm^−1^): 3424, 2924, 1439, 1118, 1023, 896, 839, 746, 689, 557. HRMS (ESI+) *m*/*z* calc. for [M]^+^ C_27_H_24_OP^+^ 395.1559, found 395.1559.

(10-Hydroxy-2,3,4-trimethoxy-9,10-dihydroanthr-9-yl)methyldiphenylphosphonium tetrafluoroborate (**15**BF_4_). Yellowish amorphous solid, yield: 73%. Isomer ratio *trans*/*cis*: 3.1:1. ^1^H NMR (500 MHz, CD_2_Cl_2_) δ 7.91–7.83 (m, 2H, C*H*_Ph_, major + minor), 7.83–7.78 (m, 2H, C*H*_Ph_, major + minor), 7.78–7.61 (m, 14H C*H*_Ph_, 2H C*H*_An_, major + minor), 7.58 (ddd, *J*_HH_ = 7.9, 7.9 Hz, *J*_PH_ = 3.6 Hz 2H, C*H*_Ph_, minor), 7.50 (dd, *J*_HH_ = 7.5, 7.5 Hz, 1H, C*H*_An_, minor), 7.40 (dd, *J*_HH_ = 7.5, 7.5 Hz, 1H, C*H*_An_, major), 7.35 (dd, *J*_HH_ = 7.6, 7.6 Hz, 1H, C*H*_An_, minor), 7.33 (dd, *J*_HH_ = 8.1, 8.1 Hz, 1H, C*H*_An_, major), 7.27 (d, *J* = 7.7 Hz, 1H, C*H*_An_, minor), 7.19 (dd, ^2^*J*_PH_ = 9.8, *J*_HH_ = 2.8 Hz, 1H, C*H*P, major), 7.01 (d, ^3^*J*_HH_ = 7.6 Hz, 1H, C*H*_An_, major), 6.93 (dd, ^3^*J*_HH_ = 7.7, ^4^*J*_PH_ = 1.2 Hz, 1H, C*H*_An_, major), 6.88 (dd, ^2^*J*_PH_ = 8.7, *J*_HH_ = 2.8 Hz, 1H, C*H*P, minor), 6.70 (d, ^3^*J*_HH_ = 7.8 Hz, 1H, C*H*_An_, minor), 6.42 (s, 2H, major), 6.41 (dd, ^5^*J*_PH_ = 7.1, *J*_HH_ = 2.8 Hz, 1H, C*H*OH, minor), 6.29 (s, 2H, minor), 5.67 (dd, ^5^*J*_PH_ = 7.0, ^3^*J*_HH_ = 2.8 Hz, 1H, C*H*OH, major), 3.77 (s, 3H, minor), 3.75 (s, 6H, major), 3.71 (s, 3H, major), 3.68 (s, 6H, minor), 2.50 (d, *J* = 13.3 Hz, 3H, major), 2.34 (d, *J* = 13.4 Hz, 3H, minor), 1.87 (br s, 1H, OH) ppm. ^13^C{^1^H} NMR (101 MHz, CD_2_Cl_2_) δ 153.6 (C), 153.5 (C), 142.6 (d, *J* = 4.9 Hz, C), 140.3 (d, *J* = 6.7 Hz, C), 138.7 (C), 138.4 (C), 135.7 (d, J = 3.1 Hz, C), 135.6 (d, *J* = 3.1 Hz, CH), 135.5 (d, *J* = 6.4 Hz, CH), 135.4 (d, *J* = 3.1 Hz, CH), 135.3 (d, *J* = 3.1 Hz, CH), 133.5 (d, *J* = 9.1 Hz, CH), 133.4 (d, J = 9.0 Hz, CH), 133.2 (d, *J* = 9.6 Hz, CH), 133.2 (*J* = 9.5 Hz, CH), 132.7 (C), 131.0 (C), 130.6 (d, *J* = 3.5 Hz, CH), 130.4 (d, *J* = 11.9 Hz, CH), 130.3 (d, *J* = 10.2 Hz, CH), 130.2 (d, *J* = 10.2 Hz, CH), 130.2 (d, *J* = 12.4 Hz, CH), 129.4 (d, *J* = 3.0 Hz, CH), 129.2 (d, *J* = 3.1 Hz, CH), 124.1 (d, *J* = 2.4 Hz, CH), 123.2 (d, *J* = 3.0 Hz, CH), 122.5 (d, *J* = 3.1 Hz, CH), 122.2 (d, *J* = 2.3 Hz, CH), 117.5 (d, *J* = 81.9 Hz, PC_Ph_, minor), 117.4 (d, *J* = 80.3 Hz, PC_Ph_, major), 115.5 (d, *J* = 81.6 Hz, PC_Ph_, major), 115.4 (d, *J* = 82.3 Hz, PC_Ph_, minor), 105.3 (CH), 104.1 (CH), 88.9 (CHOH, major), 88.2 (d, *J* = 2.4 Hz, CHOH, minor), 80.2 (d, *J* = 59.4 Hz, PCH, major), 78.7 (d, *J* = 64.2 Hz, PCH, minor), 60.4 (OMe), 60.3 (OMe), 56.1 (OMe), 56.1 (OMe), 4.6 (d, *J* = 53.2 Hz, PMe), 4.4 (d, *J* = 55.2 Hz, PMe) ppm. ^31^P{^1^H} NMR (162 MHz, CD_2_Cl_2_) δ 20.3 (major), 19.6 (minor) ppm. ^19^F{^1^H} NMR (CD_2_Cl_2_) δ −151.38, −151.43 ppm. HRMS (ESI+) *m*/*z* calc. for [M^+^] C_30_H_30_O_4_P^+^ 485.1882, found 485.1900.

(10-Hydroxy-9,10-dihydroanthr-9-yl)dimethylphenylphosphonium tetrafluoroborate (**16**BF_4_). White amorphous solid, yield: 75%. Isomer ratio *trans*/*cis*: 1.2:1. ^1^H NMR (CD_2_Cl_2_) δ 7.89–7.76 (m, 2H), 7.69–7.55 (m, 7H), 7.53–7.29 (m, 11H), 7.24–7.14 (m, 4H), 7.03–6.95 (m, 3H), 6.66 (dd, *J* = 9.6, 2.8 Hz, 1H, major), 6.50–6.38 (m, 2H), 5.74 (dd, *J* = 7.0, 2.8 Hz, 1H, minor), 2.36 (d, *J* = 12.8 Hz, 3H, major), 2.35 (d, *J* = 13.9 Hz, 3H, major), 2.28 (d, *J* = 13.8 Hz, 3H, minor), 2.16 (d, *J* = 13.8 Hz, 3H, minor) ppm. ^13^C{^1^H} NMR (101 MHz, CD_2_Cl_2_) δ 139.9, 138.2, 135.2 (d, *J* = 9.6 Hz), 132.8, 132.2 (d, *J* = 9.0 Hz), 132.0 (d, *J* = 9.0 Hz), 131.2, 130.5, 130.1, 130.0, 130.0, 129.9, 129.23, 129.0, 128.8, 128.7, 128.1, 127.1, 123.6 (d, *J* = 82.4 Hz), 122.2, 121.8, 115.9, 88.5, 88.0, 80.3 (d, *J* = 60.1 Hz), 79.1 (d, *J* = 64.4 Hz), 5.5 (d, *J* = 52.3 Hz), 4.9 (d, *J* = 50.3 Hz), 3.6 (d, *J* = 53.8 Hz), 3.4 (d, 54.9 Hz) ppm. ^31^P{^1^H} NMR (CD_2_Cl_2_) δ 23.9 (major), 23.1 (minor) ppm. ^11^B{^1^H} NMR (CD_2_Cl_2_) δ −1.02 ppm. HRMS (ESI+) *m*/*z* calc. for [M]^+^ C_22_H_22_OP^+^ 333.1403, found 333.1405.

#### 3.2.3. General Procedure for Replacing Chloride Anion with PF_6_^−^ or BPh_4_^−^: Synthesis of Phosphonium Salts *trans*-**13**PF_6_ and **14**BPh_4_

To a solution of the corresponding chloride *trans*-**13**Cl or **14**Cl (1 mmol) in DCM (3 mL), 1 equiv KPF_6_ or KBPh_4_ was added and stirred for 3 h at room temperature. The resulting KCl was separated, and the filtrate was concentrated to a white precipitate that was dried using a vacuum pump.

*Trans*-(10-hydroxy-2,3,4-trimethoxy-9,10-dihydroanthr-9-yl)triphenylphosphonium hexafluorophosphate (*trans*-**13**PF_6_). White amorphous solid, yield: 83%. ^1^H NMR (CDCl_3_) δ 7.89–7.80 (m, 3H), 7.70–7.52 (m, 12H, Ar, PCH), 7.40–7.27 (m, 3H), 7.20 (dd, *J* = 7.4 Hz, 1H), 6.93 (d, *J* = 7.7 Hz, 1H), 6.64 (dd, *J* = 7.8, 2.0 Hz, 1H), 5.18 (dd, ^5^*J*_PH_ = 7.3, ^5^*J*_HH_ = 2.7 Hz, 1H, CHOH), 3.78 (s, 9H, OMe) ppm. ^13^C{^1^H} NMR (methanol-*d*_4_) δ 153.4, 143.6 (d, *J* = 5.0 Hz), 137.9, 137.1 (d, *J* = 10.8 Hz), 136.2 (d, *J* = 6.9 Hz), 135.5 (d, *J* = 3.3 Hz), 134.6, 134.4 (d, *J* = 9.2 Hz), 133.3 (d, *J* = 19.7 Hz), 131.2, 130.6, 130.2 (d, *J* = 12.1 Hz), 128.7, 128.6, 128.3 (d, *J* = 6.9 Hz), 127.9, 123.0 (d, *J* = 4.0 Hz), 122.7 (d, *J* = 9.0 Hz), 122.3 (d, *J* = 3.0 Hz), 121.8 (d, *J* = 4.0 Hz) 116.2 (d, *J* = 81.6 Hz), 103.9, 103.7, 88.7 (CHOH), 81.2 (d, *J* = 57.7 Hz. CHP), 59.7, 55.3 ppm. ^31^P{^1^H} NMR (methanol-*d*_4_) δ 18.03, −144.29 (hept, *J* = 713.0 Hz) ppm. ^19^F{^1^H} NMR (methanol-*d*_4_) δ −74.00 (d, *J* = 707.9 Hz) ppm. HRMS (ESI+) *m*/*z* calc. for [M]^+^ C_35_H_30_O_3_P 529.1933, found 529.1925.

(10-Hydroxy-9,10-dihydroanthr-9-yl)methyldiphenylphosphonium tetraphenylborate (**14**BPh_4_). White solid, mp: 74–76 °C, yield: 88%. Isomer ratio *trans*/*cis*: 1.5:1. ^1^H NMR (CD_2_Cl_2_) δ 7.90 (dd, *J* = 8.3, 8.3 Hz, 2H, major, minor), 7.83 (dd, *J* = 8.3, 8.3 Hz, 2H, major, minor), 7.73–7.65 (m, 4H, major, minor), 7.63–7.37 (m, 32H, major, minor), 7.33 (dd, *J* = 8.2, 8.2 Hz, 1H, major), 7.26–7.15 (m, 1H major, 2H minor), 7.06 (d, *J* = 7.7 Hz, 1H, major), 7.00 (t, *J* = 7.3 Hz, 22H, major, minor), 6.95 (d, *J* = 7.8 Hz, 1H, major), 6.89–6.81 (m, 8H, major, minor), 6.59 (d, *J* = 7.4 Hz, 1H major PCH, 1H minor), 6.49 (d, *J* = 7.9 Hz, 1H, minor), 6.43 (d, *J* = 11.0 Hz, 1H, minor, C*H*OH), 6.38 (d, *J* = 9.7 Hz, 1H, minor, PCH), 5.78 (dd, *J* = 7.4, 3.1 Hz, 1H, major, C*H*OH), 1.63 (d, *J* = 12.9 Hz, 3H, major), 1.61 (d, *J* = 13.0 Hz, 3H, minor) ppm. ^13^C{^1^H} NMR (CD_2_Cl_2_) δ 164.8, 164.3, 163.8, 163.3, 142.6 (d, *J* = 6.0 Hz), 140.8 (d, *J* = 6.0 Hz), 139.4 (d, *J* = 6.0 Hz), 137.7, 136.0, 135.6 (d, *J* = 3.2 Hz), 135.5 (d, *J* = 3.2 Hz), 133.5 (d, *J* = 8.9 Hz), 133.3 (d, *J* = 9.1 Hz), 132.0 (d, *J* = 9.5 Hz), 133.0 (d, *J* = 9.5 Hz), 131.1 (d, *J* = 3.2 Hz), 130.6 (d, *J* = 5.1 Hz), 130.5 (d, *J* = 4.7 Hz), 130.4 (d, *J* = 4.7 Hz), 130.3 (d, *J* = 4.7 Hz), 129.4 (d, *J* = 9.1 Hz), 129.3 (d, *J* = 3.2 Hz), 128.9, 128.4 (d, *J* = 6.6 Hz), 128.1, 127.2, 125.8 (d, *J* = 2.8 Hz), 125.7 (d, *J* = 2.8 Hz), 124.2, 123.6 (d, *J* = 2.8 Hz), 122.1 (d, *J* = 3.3 Hz), 121.8, 117.5 (d, *J* = 24.6 Hz), 116.6 (d, *J* = 23.7 Hz), 114.7 (d, *J* = 81.0 Hz), 114.2 (d, J = 81.0 Hz), 89.0, 88.5, 80.3 (d, *J* = 60.6 Hz), 79.0 (d, *J* = 65.2 Hz), 4.1 (d, *J* = 55.0 Hz), 3.5 (d, *J* = 53.0 Hz) ppm. ^31^P{^1^H} NMR (CD_2_Cl_2_) δ 19.7 (s, major), 19.1 (s, minor) ppm. ^11^B{^1^H} NMR (CD_2_Cl_2_) δ −6.54 ppm. IR (cm^−1^): 3446, 3054, 1579, 1437, 1117, 1030, 1018, 893, 734, 705, 611. HRMS (ESI+) *m*/*z* calc. for [M]^+^ C_27_H_24_OP^+^ 395.1559, found 395.1560.

### 3.3. Synthesis of Phosphonium Salt ***18***I

To a solution of 9-bromoanthracene (0.62 g, 2.4 mmol) in dry THF (10 mL), n-butyllithium (0.9 mL, 2.7 M solution in hexane) was added dropwise at −78 °C and stirred for 30 min under argon. Then, a solution of chlorodiphenylphosphine (0.532 g, 2.4 mmol) in dry THF (7 mL) was added. The mixture was left at room temperature overnight, and then the solvent was evaporated and DMF (15 mL) and methyl iodide (2.2 mL, 3.6 mmol) were added. The homogeneous solution was heated under argon overnight at 90 °C. The solvent and excess methyl iodide were evaporated using a vacuum line. The obtained yellow precipitate was purified using a column filled with neutral aluminum oxide, eluting with a mixture of ethyl acetate/methanol (1:1 *v*/*v*).

Anthr-9-yl-methyldiphenylphosphonium iodide (**18**I). Yellow solid, mp: 138–140 °C, yield: 74%. ^1^H NMR (CD_2_Cl_2_) δ 9.11 (s, 1H), 8.34–8.26 (m, 2H), 7.95–7.86 (m, 2H), 7.85–7.71 (m, 8H), 7.67–7.59 (m, 4H), 7.52–7.44 (m, 2H), 3.17 (d, *J* = 12.4 Hz, 3H) ppm. ^13^C{^1^H} NMR (CD_2_Cl_2_) δ 140.2, 139.0, 135.5, 135.12, 132.6 (d, *J* = 10.9 Hz), 131.6, 130.9 (d, *J* = 13.0 Hz), 130.8, 129.5, 126.1, 124.6 (d, *J* = 9.1 Hz), 122.3 (d, *J* = 85.8 Hz), 16.8 (d, *J* = 59.7 Hz) ppm. ^31^P{^1^H} NMR (CD_2_Cl_2_) *δ* 17.4 ppm. HRMS (ESI+) *m*/*z* calc. for [M]^+^ C_27_H_22_P^+^ 377.1459, found 377.1462.

## 4. Conclusions

In summary, we performed a study in which we attempted to obtain anthryl phosphonium salts that, due to the presence of a positively charged phosphonium group, would exhibit a strong donor–acceptor effect. However, the Friedel–Crafts–Bradsher reaction usually used for this purpose, after the cyclization step, stopped at the stage of the intermediate anthrol, which did not undergo the further the expected Bradsher dehydration reaction, leading to a fully aromatic system, which is the first example of such behavior of diarylmethyl derivatives in the literature. Quaternary phosphonium salts are extensively used in organic synthesis as Lewis acid organocatalysts [21,29,30], reagents [23,31] and ionic liquids [32], and they also demonstrate a strong antimicrobial activity [33,34]. Therefore, the results obtained provide stimulus for further research into this phenomenon, especially in terms of studying the intramolecular interactions involving various anions and other 1,2-disubstituted anthrols.

## Figures and Tables

**Table 1:** ^1^H and ^13^C{^1^H} NMR data for compounds **11**A–**16**A ^1^.

Compound ^2^	^1^H NMR				^13^C{^1^H} NMR			Isomer Ratio ^4^
	*δ*PC*H* (ppm)	^2^*J*_PH_ (Hz)	*δ*C*H*OH (ppm)	^5^*J*_PH_ (Hz)	*δ*P*C*H (ppm)	^1^*J*_PC_ (Hz)	*δC*HOH(^4^*J*_CP_) (ppm)	*trans*:*cis* (Isolated Yield%)
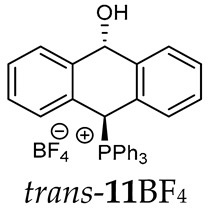	- ^3^	-	5.15	7.5	81.6	57.0	88.7	3.6:1 (79)
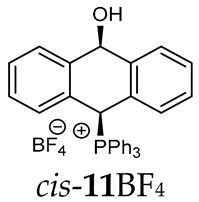	7.40	8.0	6.36	11.3	79.9	56.0	88.4	
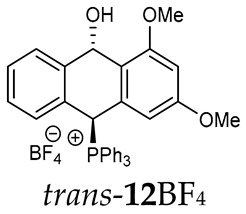	7.47	11.0	5.03	7.4	81.9	58.2	88.6	3.9:1 (54)
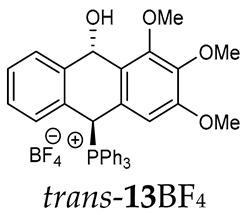	8.15	10.1	5.49	6.7	80.1	57.1	87.6	5:1 (57)
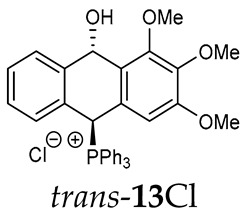	8.41	10.2	5.25	7.1	81.9	55.7	88.8	12.3:1 (18)
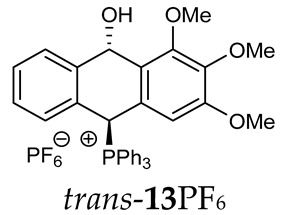	6.64	7.8	5.18	7.3	81.2	57.7	88.7	(83)
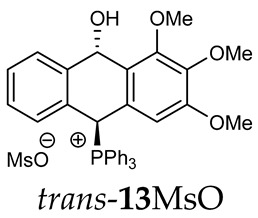	8.20	10.2	5.09	7.3	81.6	55.8	88.8	9:1 (38)
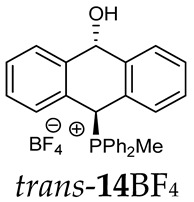	7.15	9.8	5.71	7.1	80.3	59.5	88.8	1.4:1 (95)
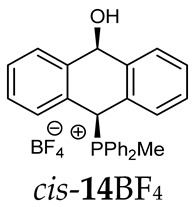	- ^3^	-	6.46	11.0	78.9	65.0	88.3 (2.7)	
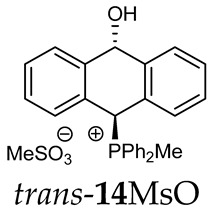	8.20	10.2	5.09	7.3	81.6	55.8	88.8	1.2:1 (83)
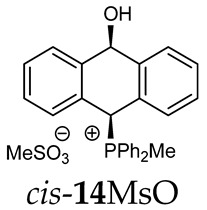	7.28	8.3	6.42	11.0	78.9	64.1	88.1 (2.7)	
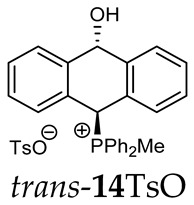	7.74	7.6	5.59	7.2	80.4	57.9	88.4	1.3:1 (85)
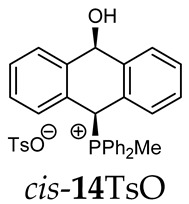	7.44	7.5	6.27	10.7	78.7	64.0	87.9	
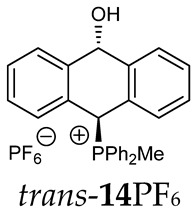	7.26	8.1	5.74	7.1	80.4	59.7	88.8	1.4:1 (83)
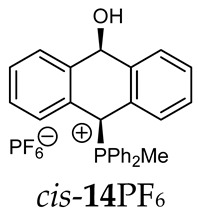	7.03	9.1	6.46	11.1	79.1	64.4	88.3 (2.4)	
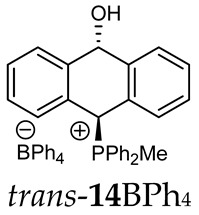	6.59	7.4	5.78	7.3	80.3	60.9	89.0	1.5:1 (88)
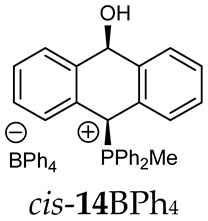	6.39	9.6	6.4	10.9	79.0	65.6	88.5 (2.3)	
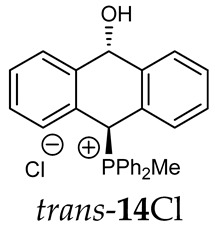	8.32	7.8	5.5	7.5	80.9	57.3	88.3	1.4:1 (82)
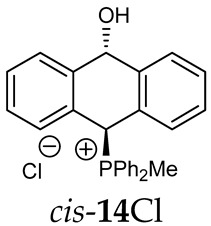	8.09	8.0	6.3	11.3	79.4	62.5	87.9 (2.8)	
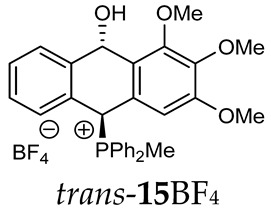	7.19	9.8	5.7	7.0	80.2	59.4	88.9	3.1:1 (73)
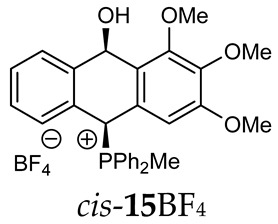	6.88	8.7	6.4	7.1	78.7	64.2	88.2 (2.4)	
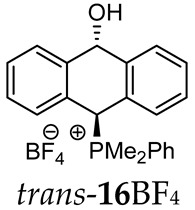	- ^3^	-	5.7	7.0	80.3	60.1	88.5	1.2:1 (75)
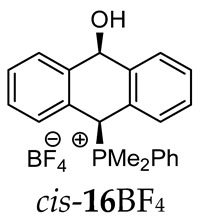	- ^3^	-	6.7	9.6	79.1	64.4	88.0 (2.0)	

^1^ Procedure for **11**A–**16**A: phosphine (1 equiv.), acid (1 equiv.) and o-acetalaryl(aryl)methanol (1 equiv.) **2**, **3** or **4** in DCM were reacted for 1 h at room temperature followed by addition of water, extraction with DCM and drying. For **14**A–**16**A, evaporation of DCM and washing with ethyl acetate gave pure products. Purification of **11**A–**13**A required flash chromatography of crude mixture over neutral Al_2_O_3_. Procedure for *trans*-**13**PF_6_ and **14**BPh_4_: *trans*-**13**Cl or **14**Cl (1 equiv.) and KPF_6_ or KBPh_4_ (1 equiv.) were reacted in DCM for 3 h at room temperature. Pure products were obtained after KCl separation and DCM evaporation. ^2^ Due to the instability of the minor isomer of compounds **12** and **13**, only the major isomer was characterized. The minor and major isomers were not separated and were characterized as a mixture. The measurements were recorded in CD_2_Cl_2_ except for the compound *trans*-**13**PF_6_, which was measured in MeOD. ^3^ Signals overlapped with the aromatic system. ^4^ Ratio determined for a crude mixture.

**Table 2:** FT-IR characteristic experimental and theoretical vibrations for the compounds **14**Cl.

Assignment	Observed Frequencies (cm^−1^)	Calculated Frequencies (cm^−1^)	
	14Cl	14Cl *trans*-A	16Cl *cis*-C
υ(O-H)	3393	3376	2760
υ(C-H)^equatorial^	2876	-	2916
υ(C-H)^axial^	2819	2859	-
δ(O-H)	1372	1386	1390
δ(O-H)	1304	1329	1330
υ(C-O)^equatorial^	1115	1049	-
υ(C-O)^axial^	1016	-	1017

υ—stretching, δ—bending.

## Data Availability

Data is contained within the article and supplementary material.

## Supplementary Materials

The following supporting information can be downloaded at: https://www.mdpi.com/article/10.3390/ijms25031741/s1.
