# Supplementary material for: The First Example of the Friedel–Crafts Cyclization Leading to (10-Hydroxy-9,10-dihydroanthr-9-yl)phosphonium Salts without the Expected Bradsher Dehydration
Source: Int J Mol Sci. 2024 Feb 1;25(3):1741. doi: 10.3390/ijms25031741 (PMC10855353; doi:10.3390/ijms25031741)
Supplement: Supplementary file 1 [file ijms-25-01741-s001.zip › ijms-2802383-supplementary.pdf]

Supplementary Materials (SM)

The First Example of the Friedel–Crafts Cyclization Leading to (10-Hydroxy-9,10-dihydroanthr-9-yl)phosphonium Salts without the Expected Bradsher Dehydration

Krzysztof Owsianik [1,*], Ewa Różycka-Sokołowska [2], Marek Koprowski [1], Marika Turek [2], Łucja Knopik [1,3], Vivek Vivek [1,3], Bogdan Dudziński [1] and Piotr Bałczewski [1,2,*]

[1] Division of Organic Chemistry, Centre of Molecular and Macromolecular Studies, Polish Academy of Sciences, Sienkiewicza 112, 90-363 Łódź, Poland; marek.koprowski@cbmm.lodz.pl (M.K.); lucja.knopik@cbmm.lodz.pl (Ł.K.); vivek@cbmm.lodz.pl (V.V.); bogdan.dudzinski@cbmm.lodz.pl (B.D.)

[2] Institute of Chemistry, Faculty of Science and Technology, Jan Długosz University in Częstochowa, Armii Krajowej 13/15, 42-201 Częstochowa, Poland; e.sokolowska@ujd.edu.pl (E.R.-S.); marika.turek93@gmail.com (M.T.)

[3] The Bio-Med-Chem Doctoral School of the University of Łódź and Łódź Institutes of the Polish Academy of Sciences, University of Łódź, Matejki 21/23, 90-237 Łódź, Poland

* Correspondence: krzysztof.owsianik@cbmm.lodz.pl (K.O.); piotr.balczewski@cbmm.lodz.pl (P.B.)

Table of contents

NMR spectra	S2-S66
FT IR spectra	S67-S69

NMR Spectra

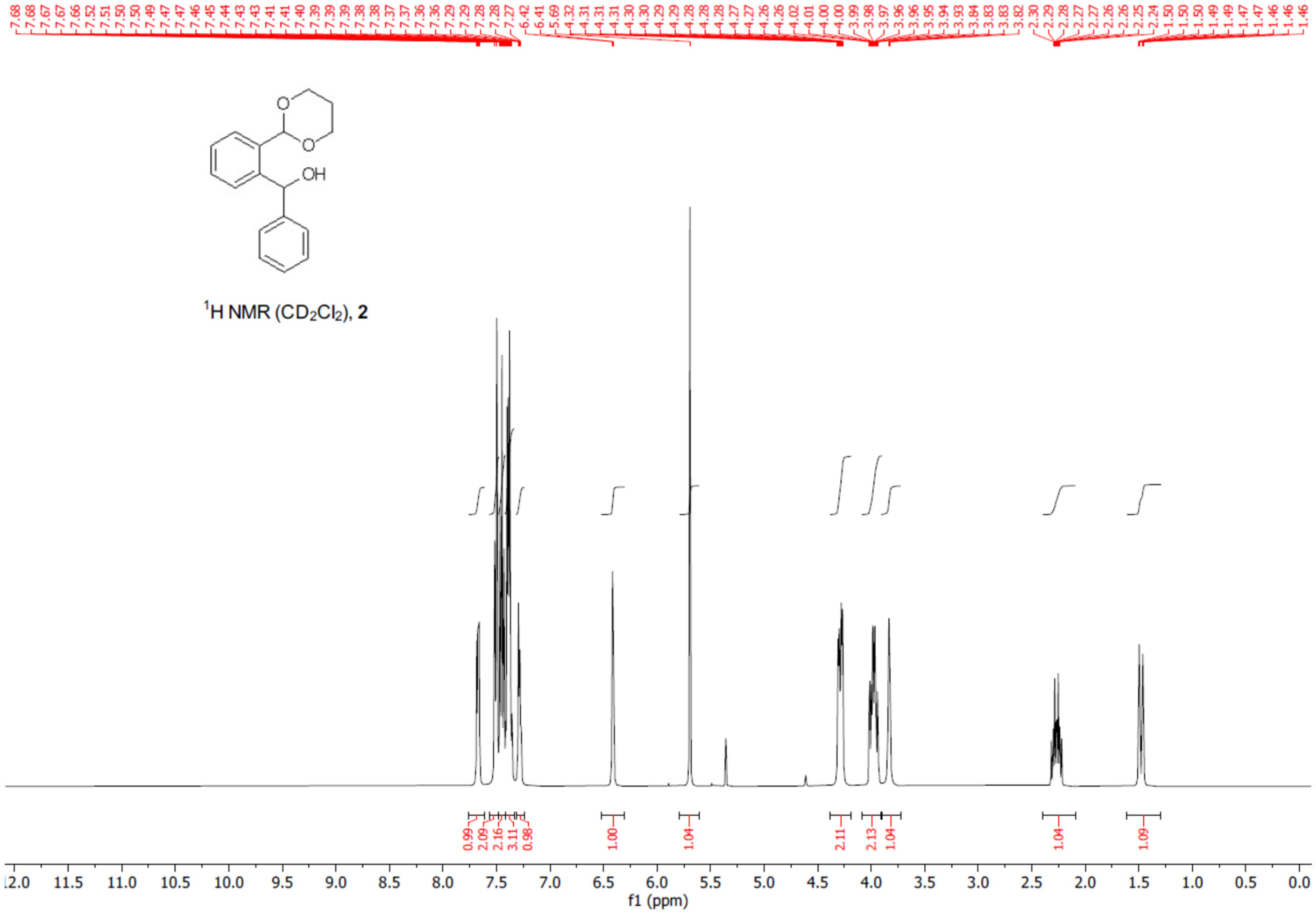
7.68 7.68 7.67 7.66 7.52 7.51 7.50 7.50 7.49 7.47 7.47 7.46 7.45 7.44 7.43 7.43 7.41 7.41 7.40 7.39 7.39 7.39 7.38 7.38 7.37 7.36 7.36 7.29 7.29 7.28 7.28 7.27 6.42 6.41 5.69 4.32 4.31 4.31 4.30 4.30 4.29 4.29 4.28 4.28 4.28 4.27 4.27 4.26 4.26 4.02 4.01 4.00 4.00 3.99 3.98 3.97 3.96 3.96 3.95 3.94 3.93 3.84 3.83 3.83 3.82 2.30 2.29 2.28 2.27 2.27 2.26 2.26 2.25 2.24 1.50 1.50 1.50 1.49 1.49 1.47 1.47 1.46 1.46 1.46
OH
O
O
1H NMR (CD2Cl2), 2
0.99 2.09 2.16 3.11 0.98 1.00 1.04 2.11 2.13 1.04 1.04 1.09
12.0 11.5 11.0 10.5 10.0 9.5 9.0 8.5 8.0 7.5 7.0 6.5 6.0 5.5 5.0 4.5 4.0 3.5 3.0 2.5 2.0 1.5 1.0 0.5 0.0
f1 (ppm)

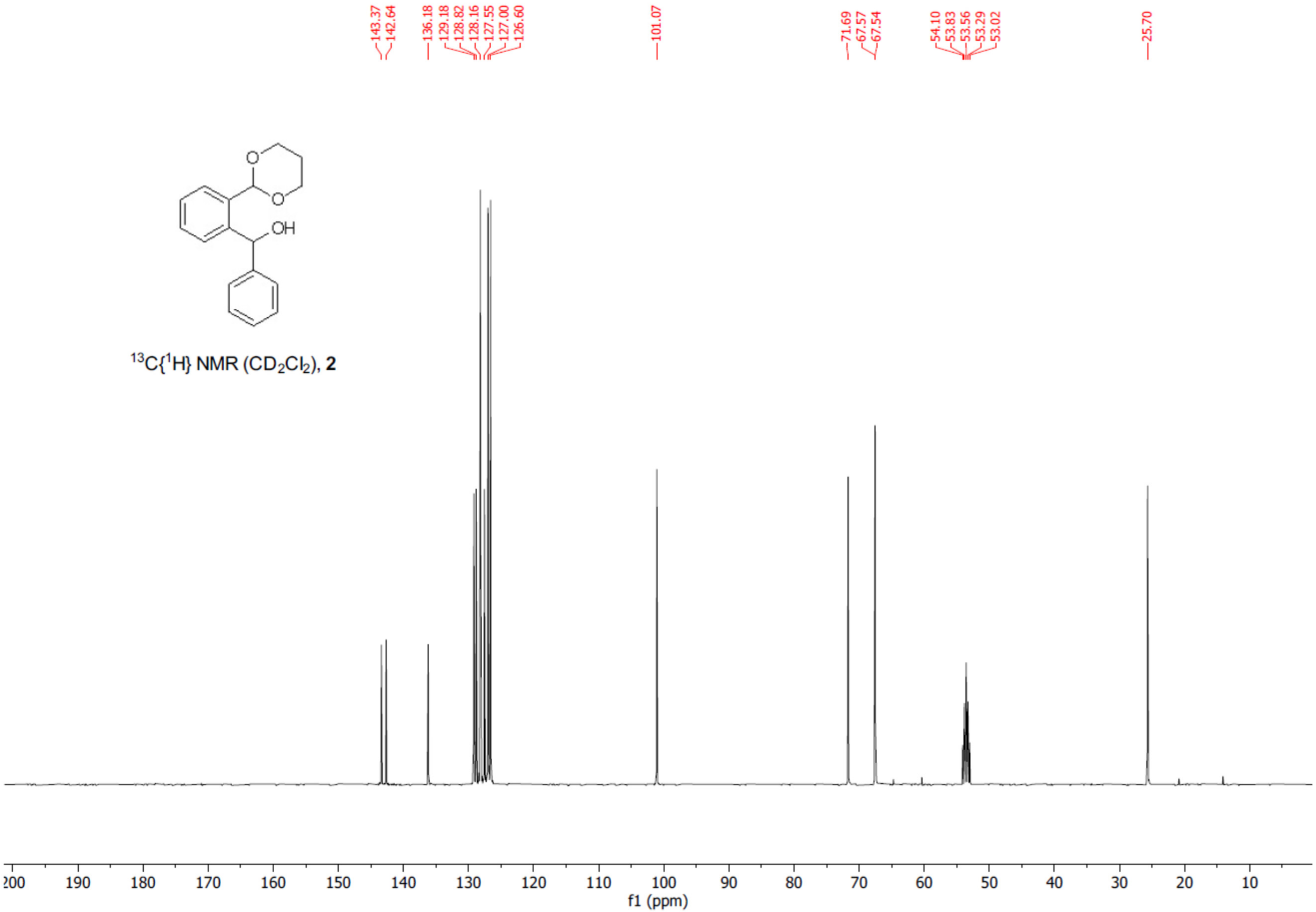
143.37
142.64
136.18
129.18
128.82
128.16
127.55
127.00
126.60
101.07
71.69
67.57
67.54
54.10
53.83
53.56
53.29
53.02
25.70
OH
O
O
$^{13}C\{^1H\}$ NMR (CD_2Cl_2), **2**
200
190
180
170
160
150
140
130
120
110
100
90
80
70
60
50
40
30
20
10
f1 (ppm)

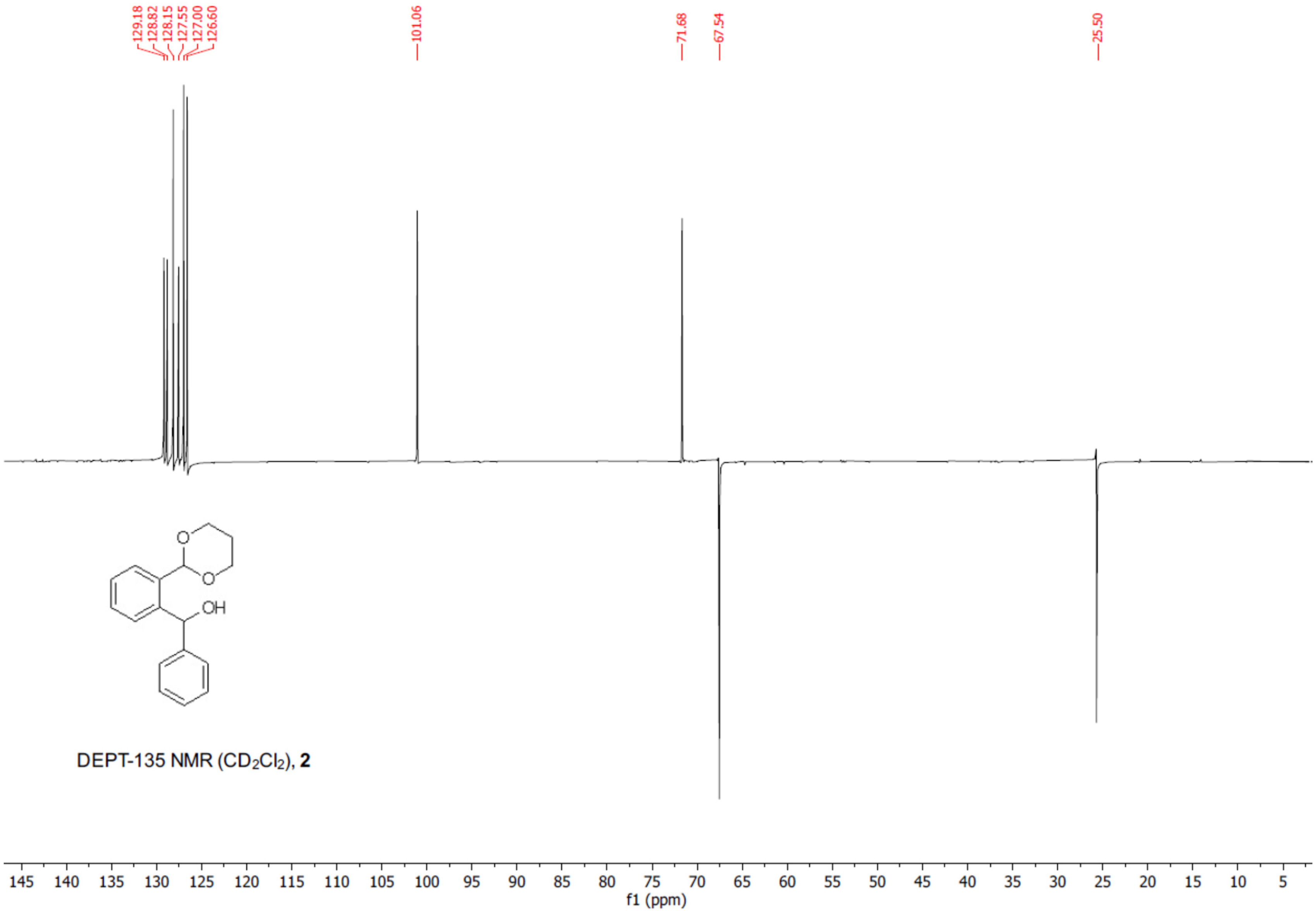

DEPT-135 NMR (CD_2Cl_2), **2**

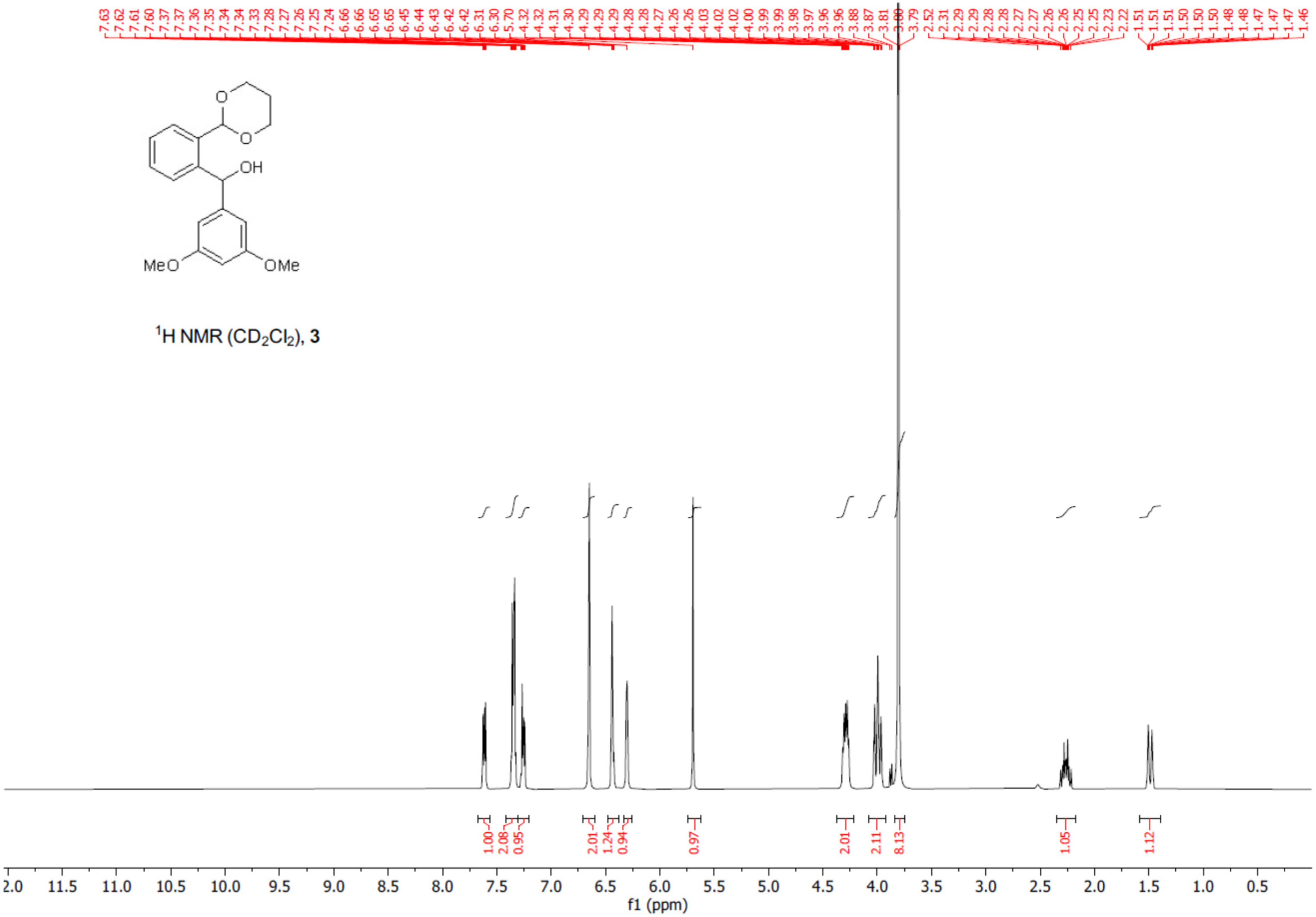
MeO
OMe
OH
^{1}H NMR (CD_2Cl_2), **3**
f1 (ppm)

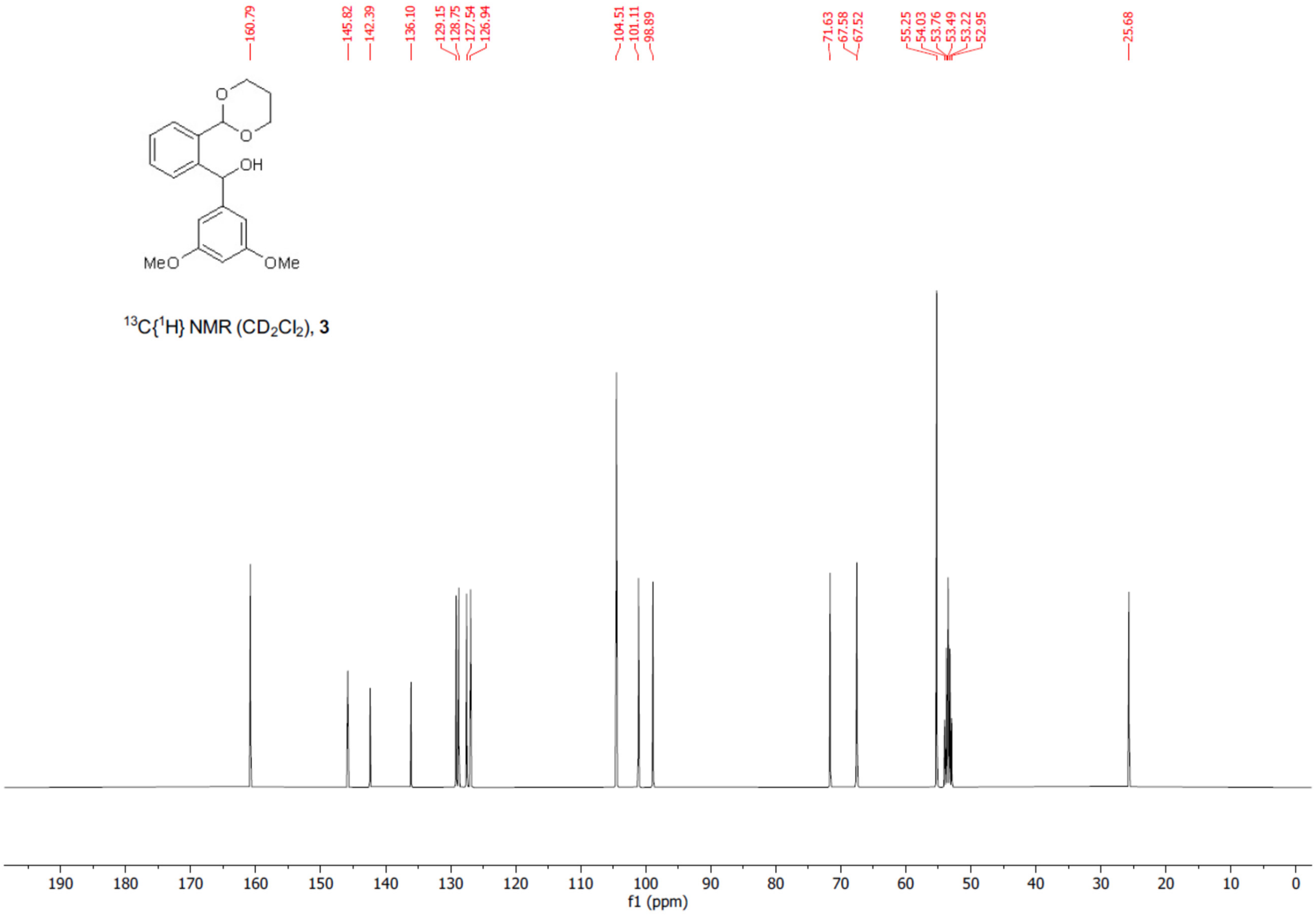

$^{13}C\{^1H\}$ NMR (CD_2Cl_2), **3**

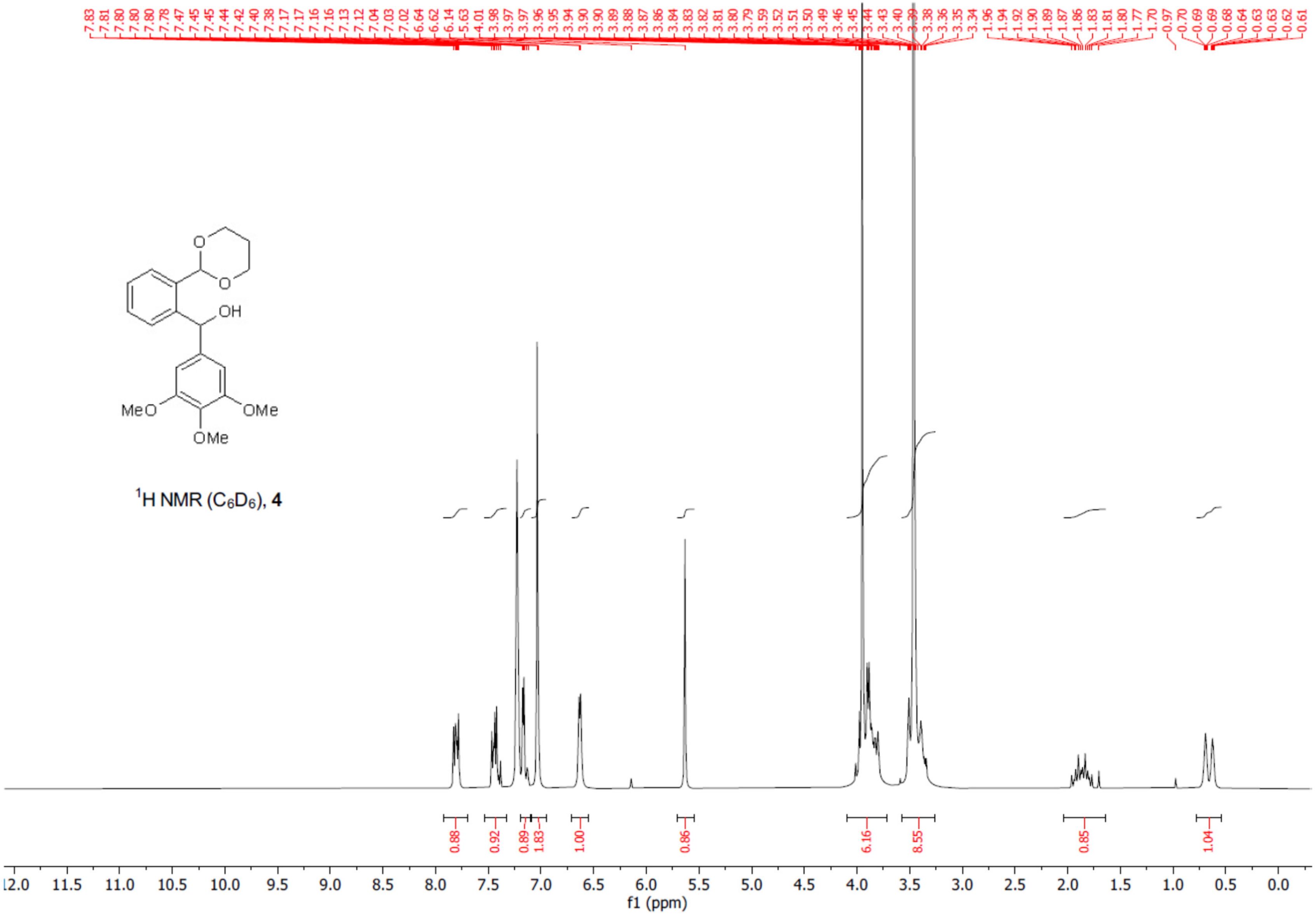
7.83 7.81 7.80 7.80 7.78 7.47 7.45 7.45 7.44 7.42 7.40 7.38 7.17 7.17 7.16 7.13 7.12 7.04 7.03 7.02 6.64 6.62 6.14 5.63 4.01 3.98 3.97 3.97 3.96 3.95 3.94 3.90 3.90 3.89 3.88 3.87 3.86 3.84 3.83 3.82 3.81 3.80 3.79 3.59 3.52 3.51 3.50 3.49 3.46 3.45 3.44 3.43 3.40 3.39 3.38 3.36 3.35 3.34 1.96 1.94 1.92 1.90 1.89 1.87 1.86 1.83 1.81 1.80 1.77 1.70 0.97 0.70 0.69 0.69 0.68 0.64 0.63 0.63 0.62 0.61
OH
MeO
OMe
OMe
O
O
^{1}H NMR (C_6D_6), **4**
0.88
0.92
0.89
1.83
1.00
0.86
6.16
8.55
0.85
1.04
12.0 11.5 11.0 10.5 10.0 9.5 9.0 8.5 8.0 7.5 7.0 6.5 6.0 5.5 5.0 4.5 4.0 3.5 3.0 2.5 2.0 1.5 1.0 0.5 0.0
f1 (ppm)

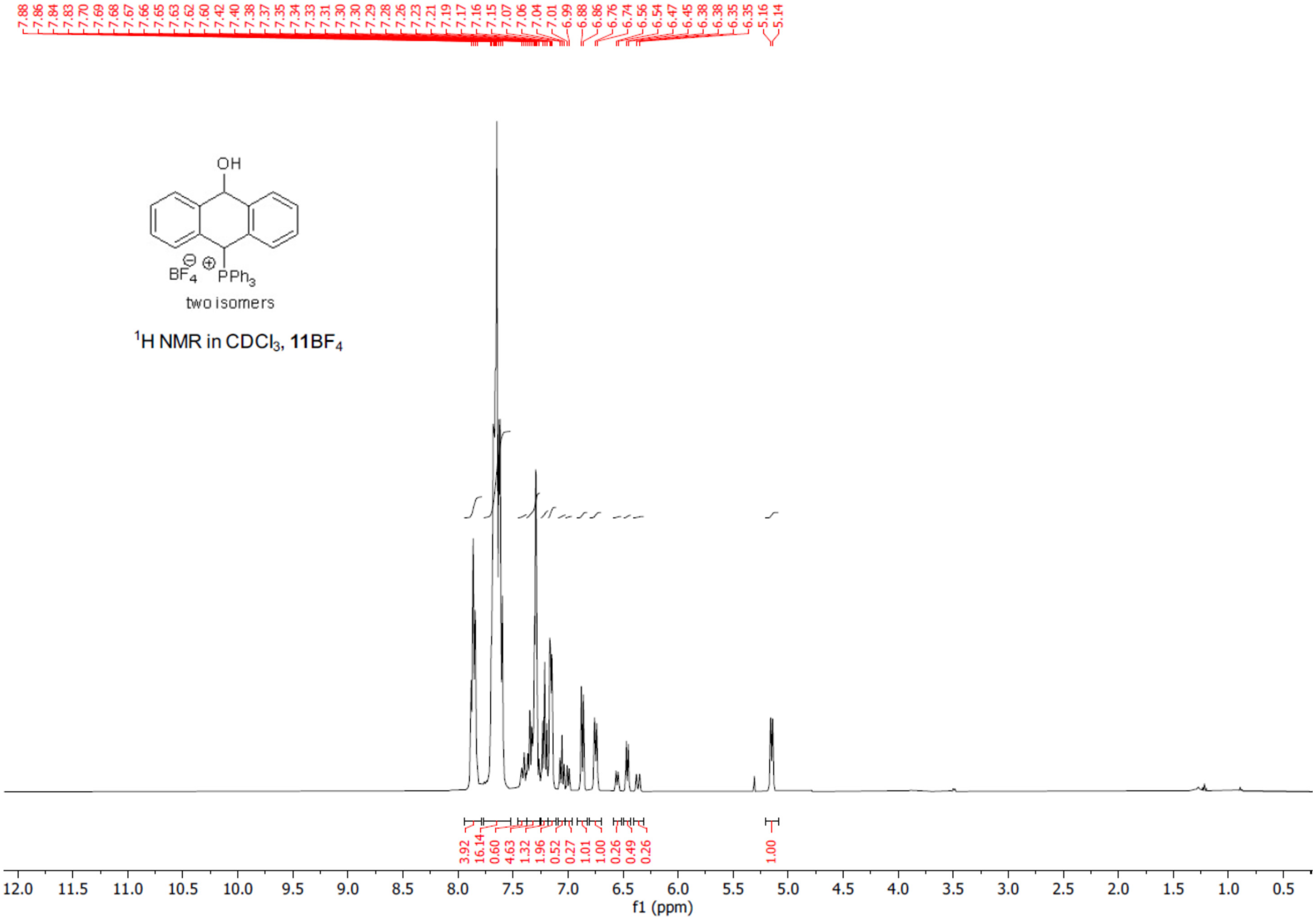

OH
BF4
PPh3
two isomers
1H NMR in CDCl3, 11BF4
f1 (ppm)

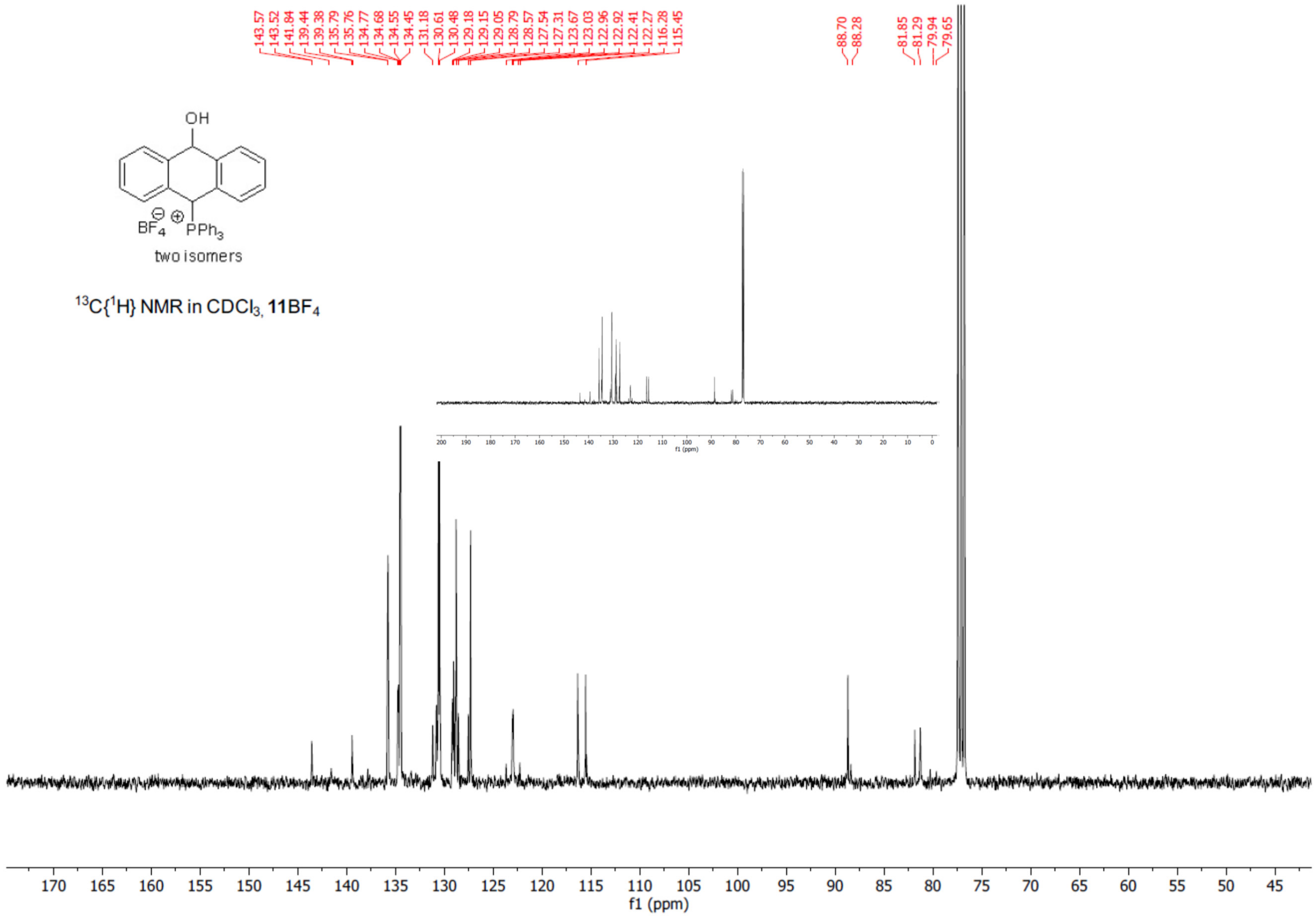
143.57
143.52
141.84
139.44
139.38
135.79
135.76
134.77
134.68
134.55
134.45
131.18
130.61
130.48
129.18
129.15
129.05
128.79
128.57
127.54
127.31
123.67
123.03
122.96
122.92
122.41
122.27
116.28
115.45
88.70
88.28
81.85
81.29
79.94
79.65
OH
BF4
PPh3
two isomers
13C{1H} NMR in CDCl3, 11BF4
f1 (ppm)

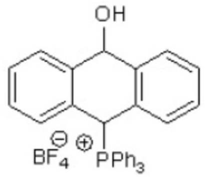

two isomers

$^{31}P\{^1H\}$ NMR in $CDCl_3$, **11BF$_4$**

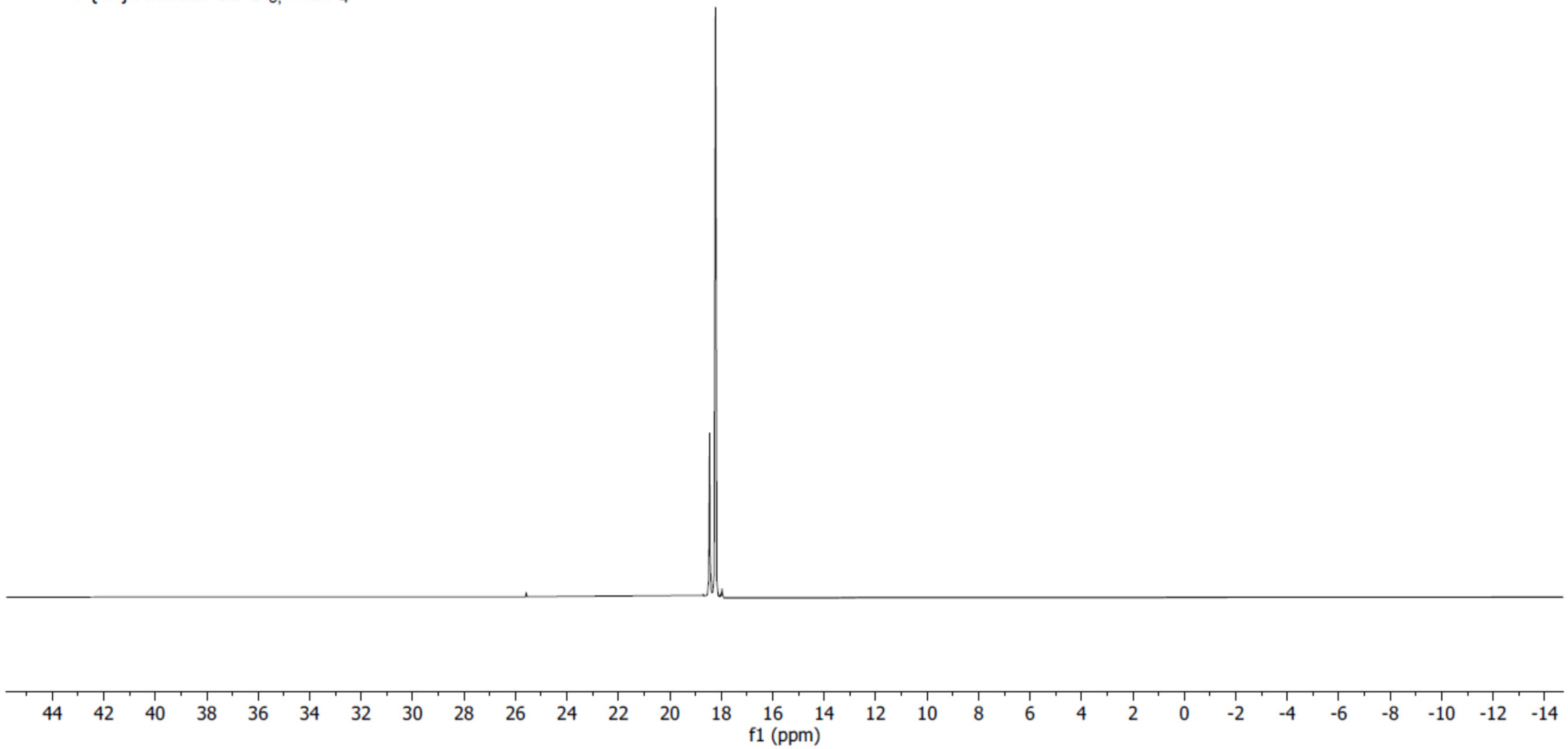

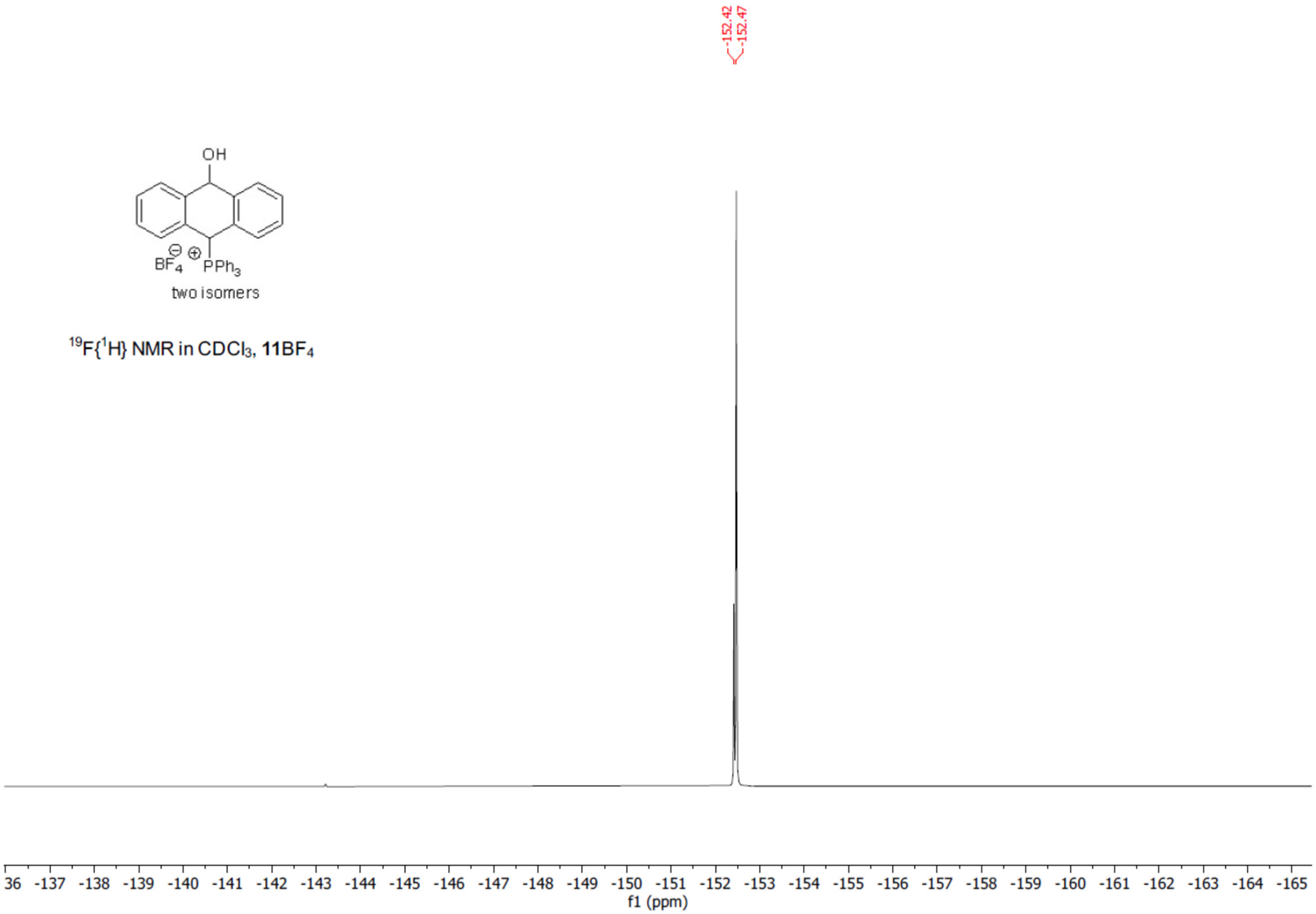
-152.42
-152.47
OH
BF4 PPh3
two isomers
19F{1H} NMR in CDCl3, 11BF4
36 -137 -138 -139 -140 -141 -142 -143 -144 -145 -146 -147 -148 -149 -150 -151 -152 -153 -154 -155 -156 -157 -158 -159 -160 -161 -162 -163 -164 -165
f1 (ppm)

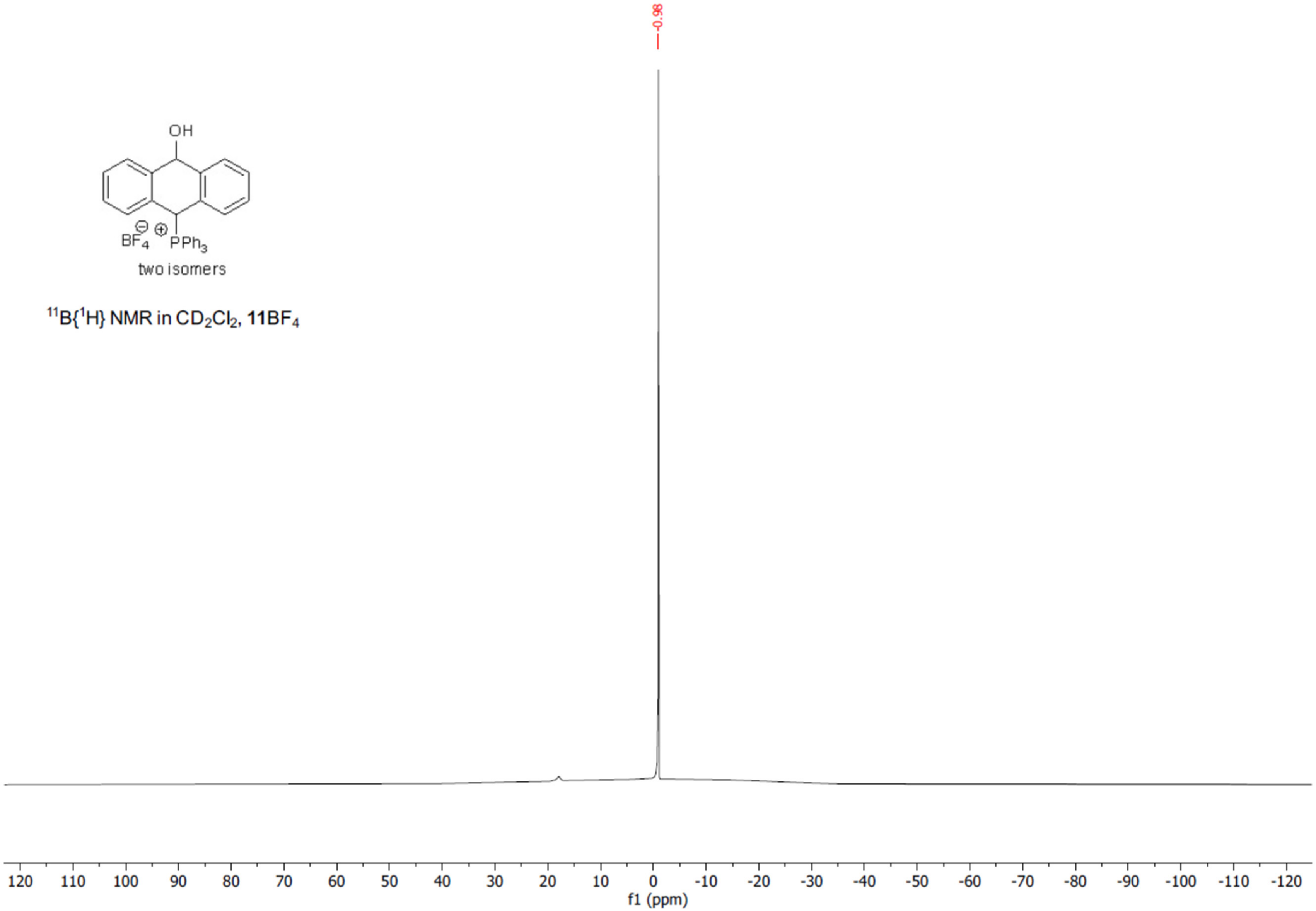

$^{11}B\{^1H\}$ NMR in CD_2Cl_2, **$11BF_4$**

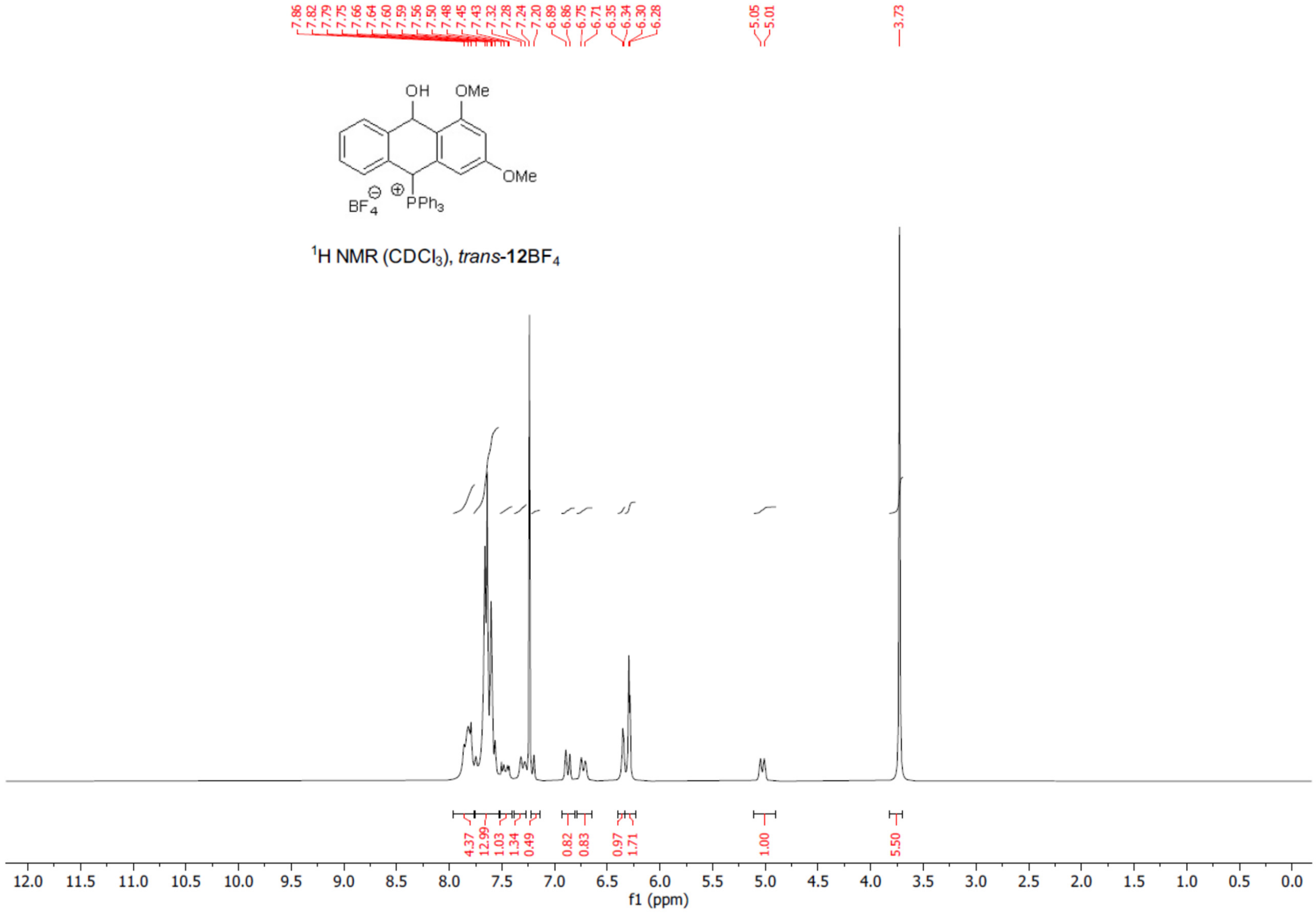
OH
OMe
OMe
BF4
PPh3
1H NMR (CDCl3), trans-12BF4
7.86
7.82
7.79
7.75
7.66
7.64
7.60
7.59
7.56
7.50
7.48
7.45
7.43
7.32
7.28
7.24
7.20
6.89
6.86
6.75
6.71
6.35
6.34
6.30
6.28
5.05
5.01
3.73
4.37
12.99
1.03
1.34
0.49
0.82
0.83
0.97
1.71
1.00
5.50
f1 (ppm)

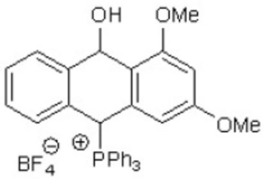

$^{13}C\{^1H\}$ NMR (CD_2Cl_2), *trans*-**12**BF_4

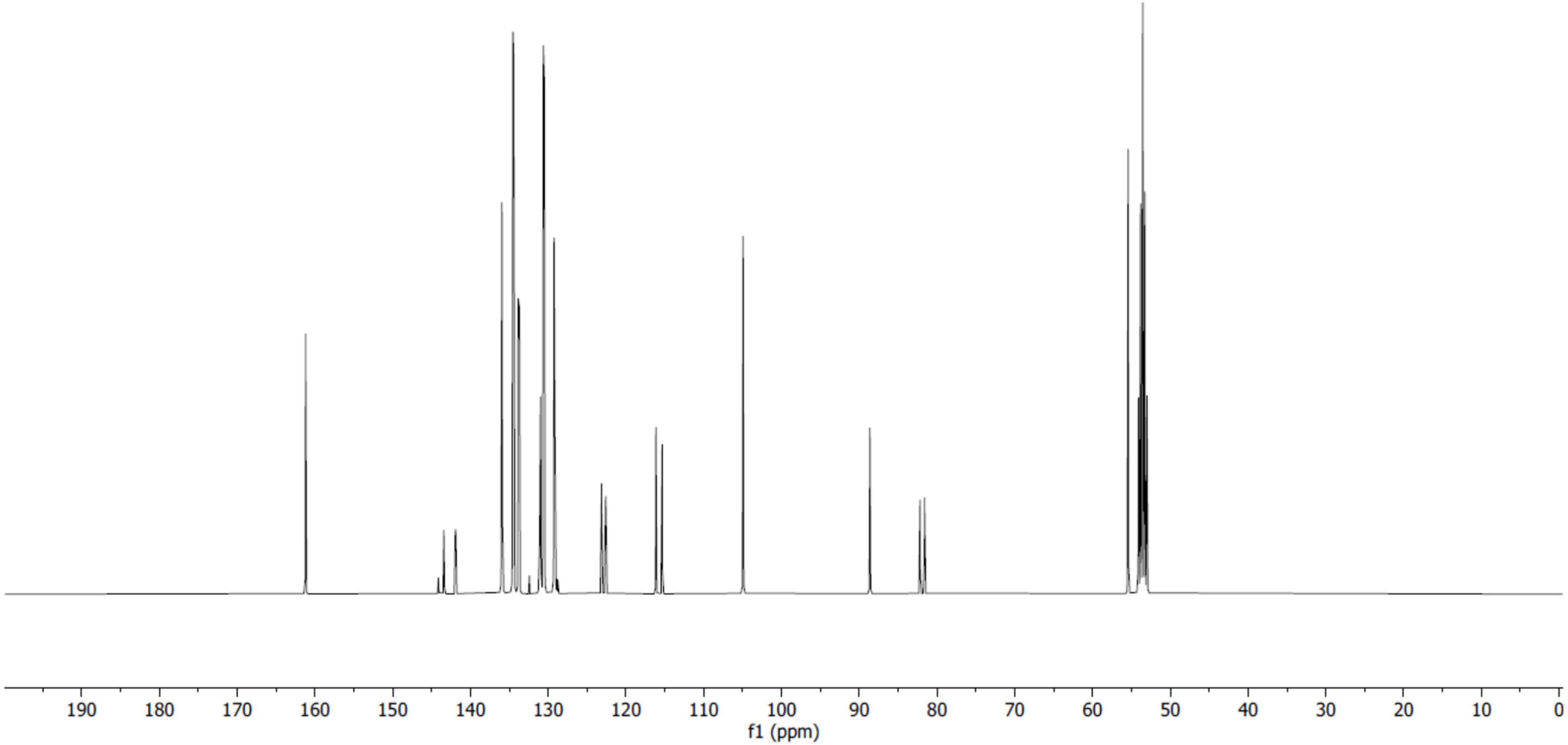

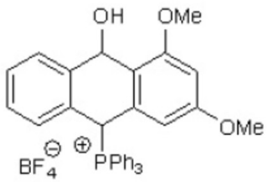

$^{31}P\{^1H\}$ NMR ($CDCl_3$), *trans*-**12**BF_4

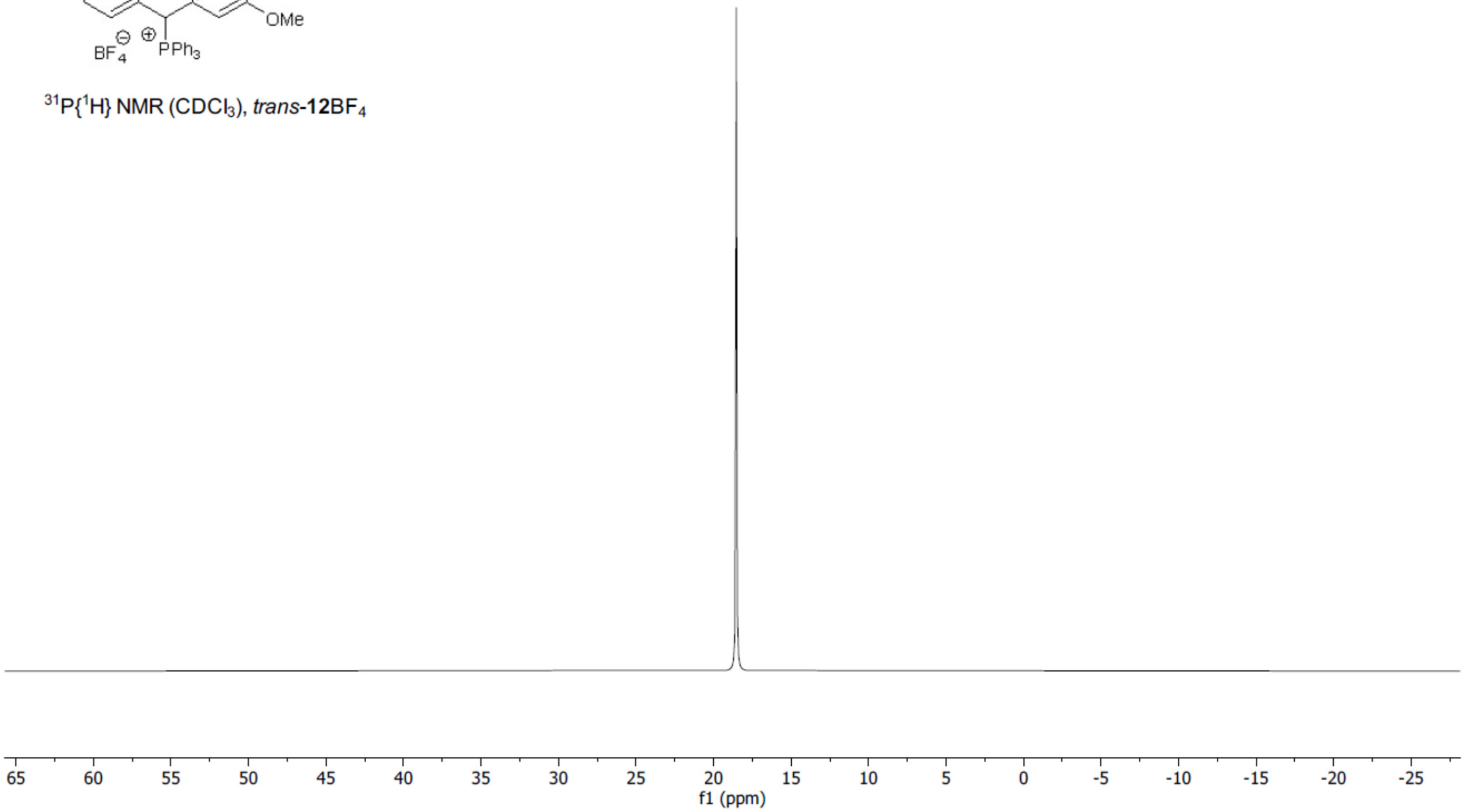

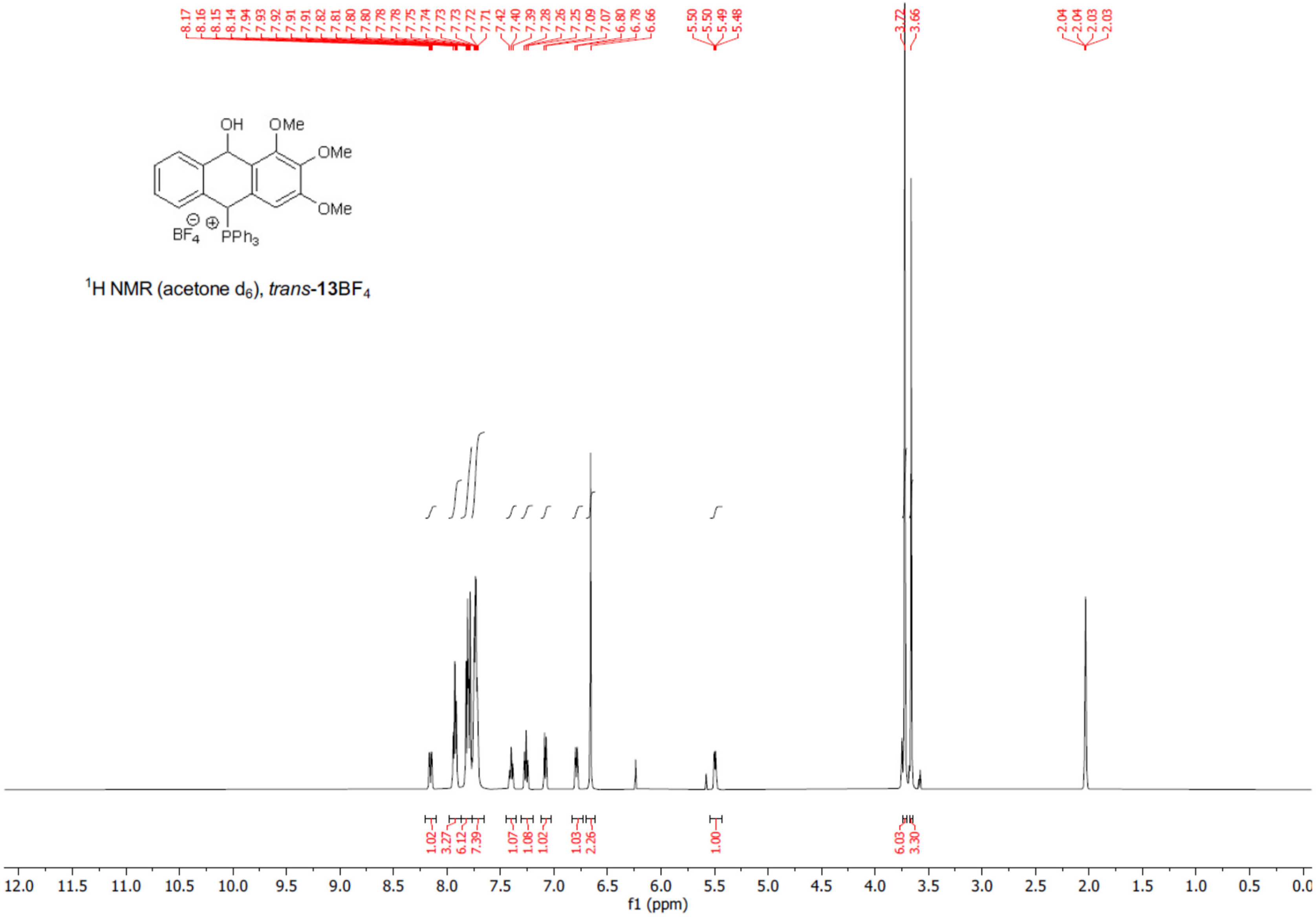
OH
OMe
OMe
OMe
BF4
PPh3
1H NMR (acetone d6), trans-13BF4
8.17 8.16 8.15 8.14 7.94 7.93 7.92 7.91 7.91 7.82 7.81 7.80 7.80 7.78 7.78 7.78 7.75 7.74 7.73 7.73 7.72 7.71 7.42 7.40 7.39 7.28 7.26 7.25 7.09 7.07 6.80 6.78 6.66
5.50 5.50 5.49 5.48
3.72 3.66
2.04 2.04 2.03 2.03
1.02 3.27 6.12 7.39 1.07 1.08 1.02 1.03 2.26 1.00 6.03 3.30
12.0 11.5 11.0 10.5 10.0 9.5 9.0 8.5 8.0 7.5 7.0 6.5 6.0 5.5 5.0 4.5 4.0 3.5 3.0 2.5 2.0 1.5 1.0 0.5 0.0
f1 (ppm)

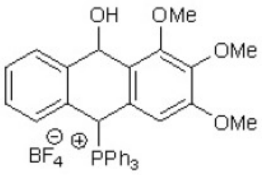

$^{13}C\{^{1}H\}$ NMR ($CDCl_3$), *trans*-**13**BF_4

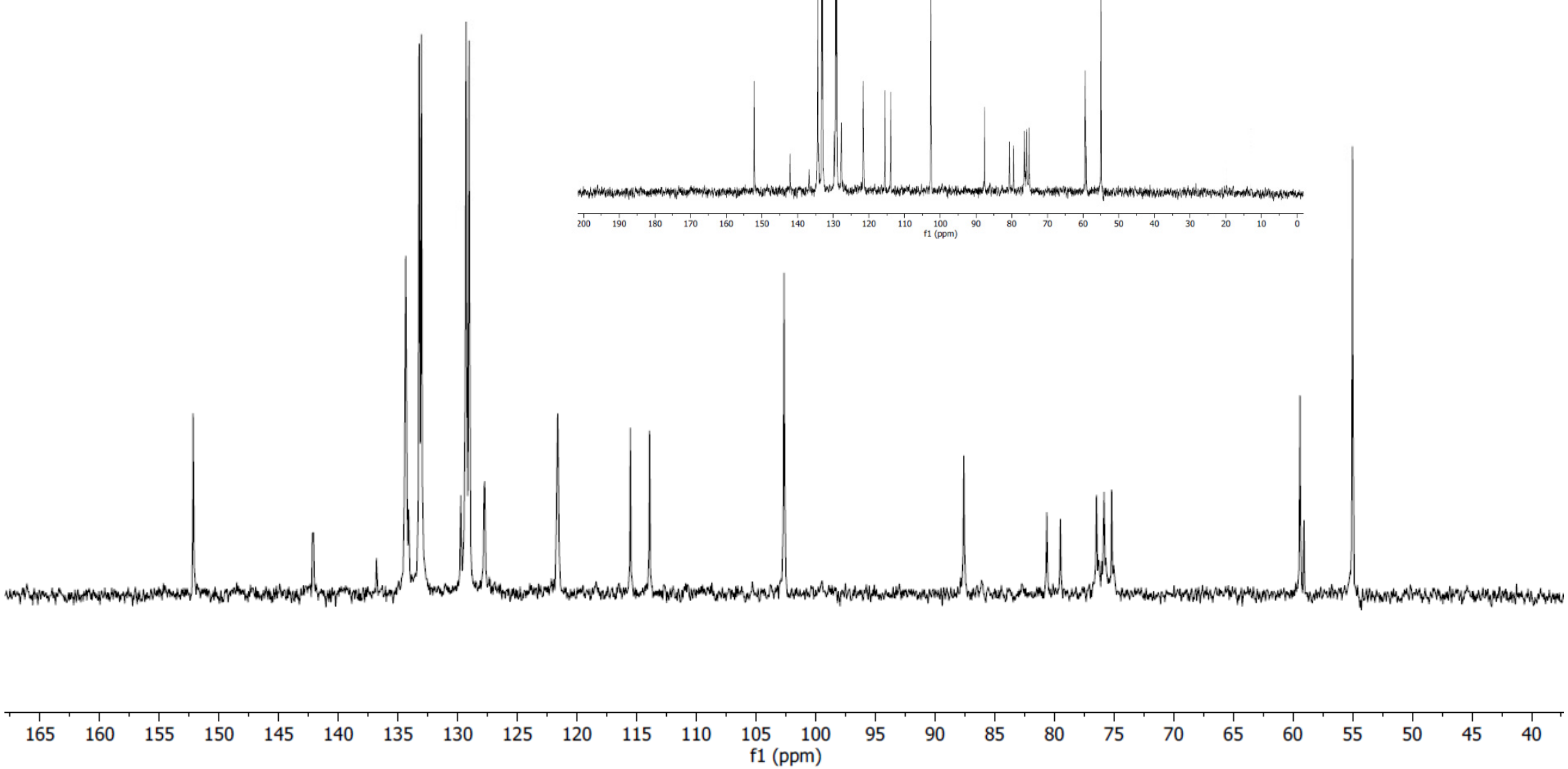

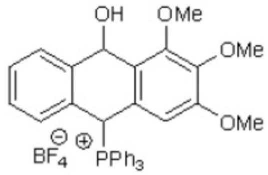

$^{31}P\{^{1}H\}$ NMR (acetone d-6) *trans*-**13**BF_4

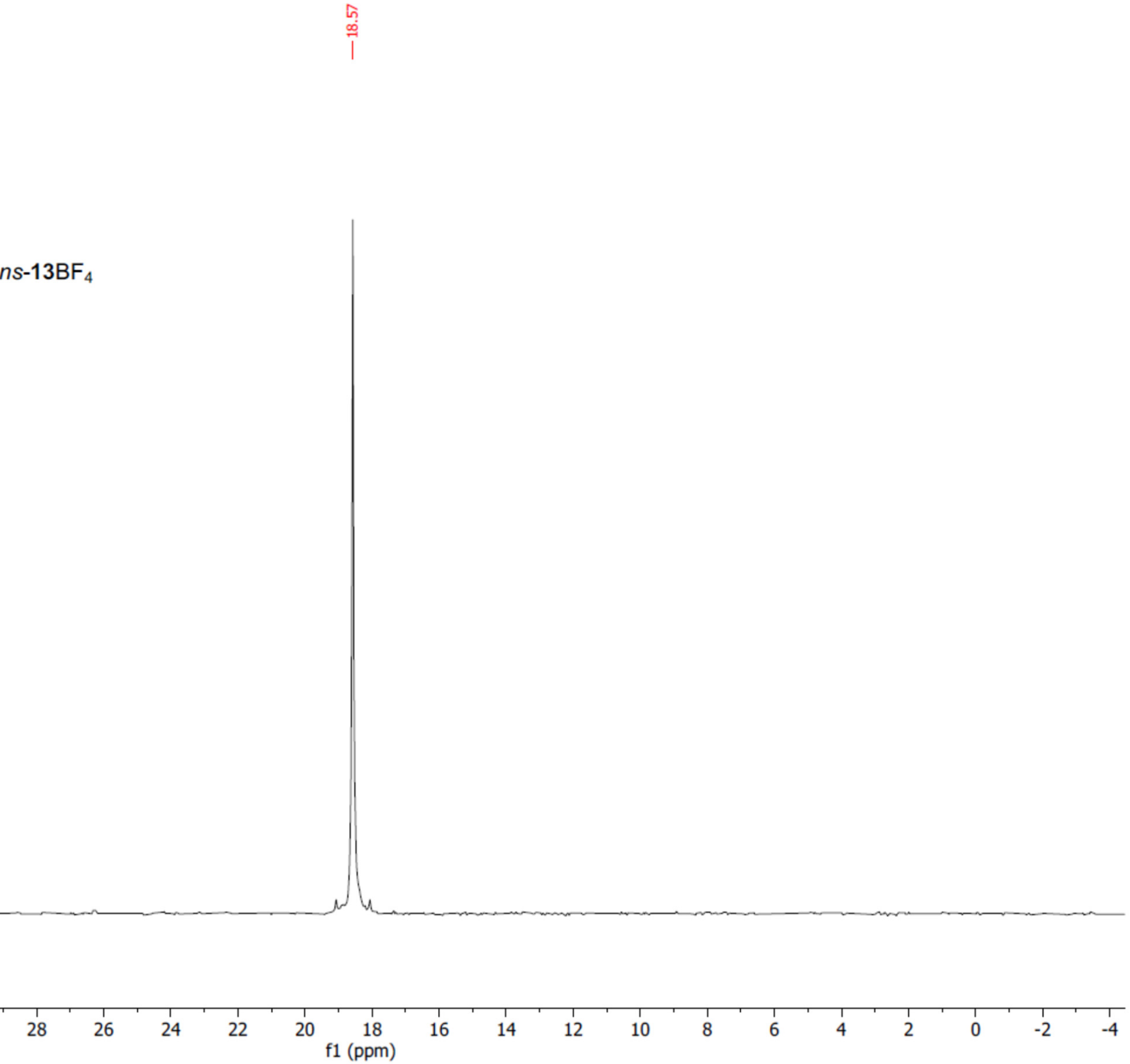

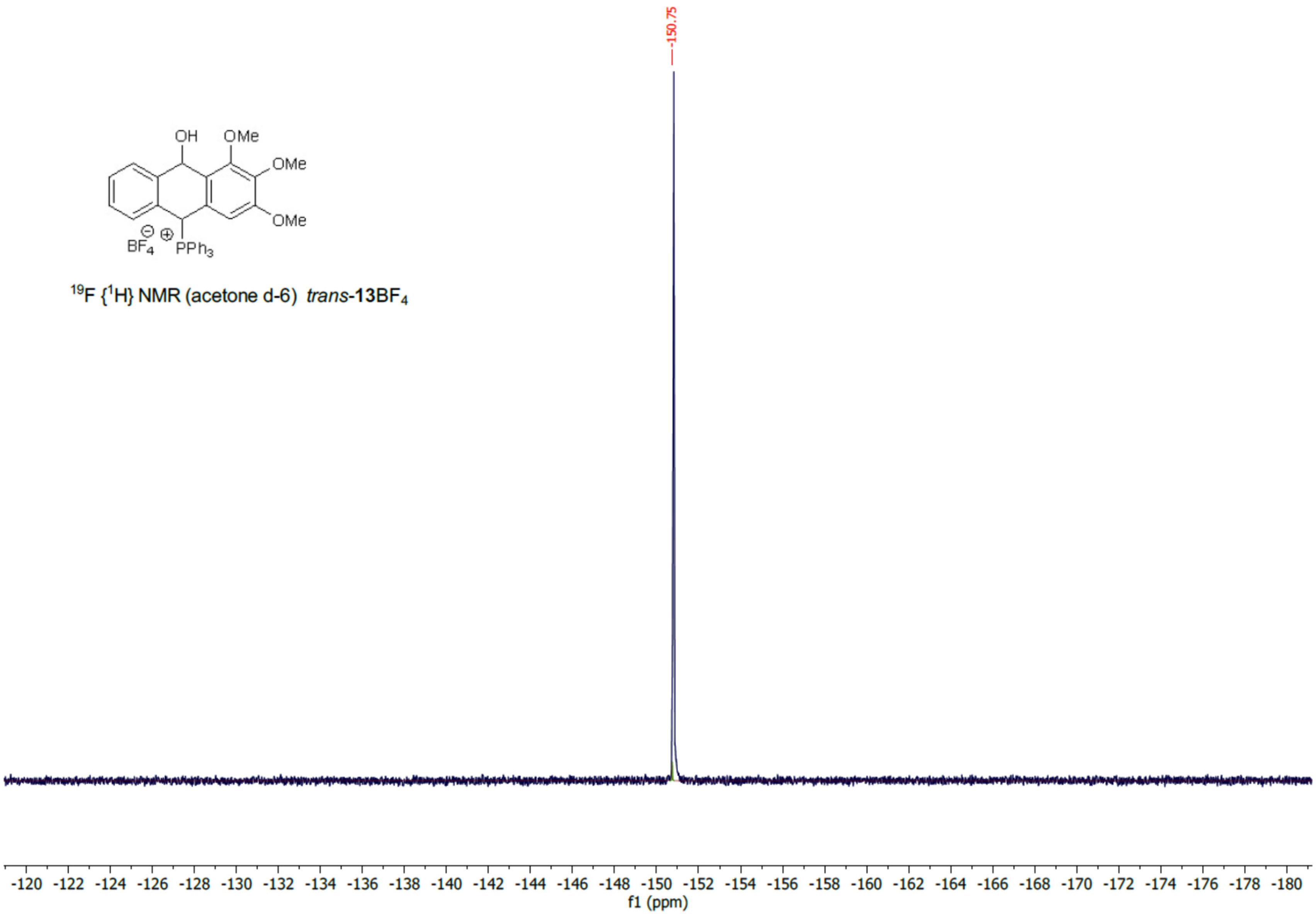

^{19}F {^{1}H} NMR (acetone d-6) *trans*-**13**BF_4

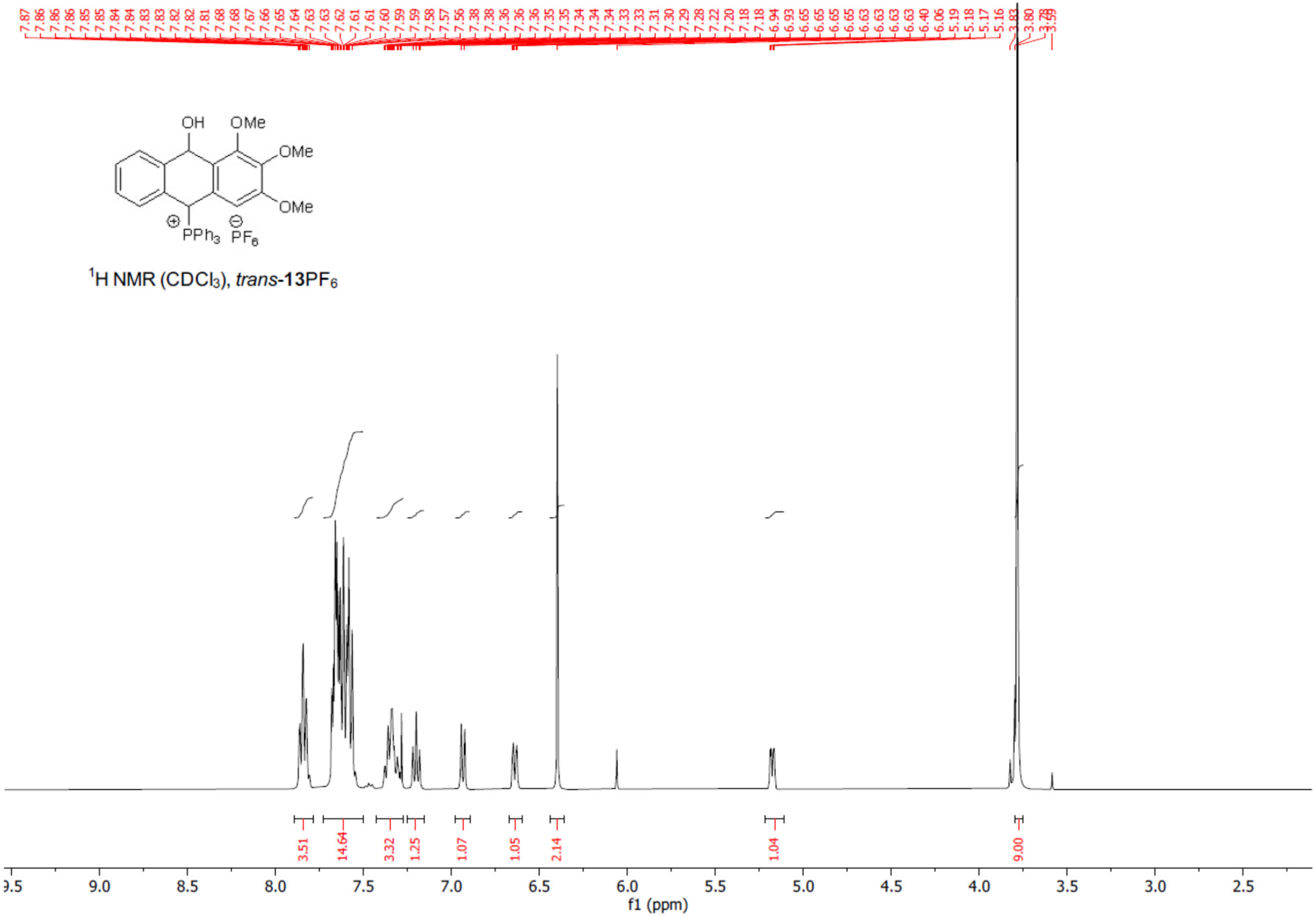
7.87 7.86 7.86 7.85 7.85 7.84 7.84 7.83 7.83 7.82 7.82 7.81 7.68 7.68 7.67 7.66 7.65 7.64 7.63 7.63 7.62 7.61 7.61 7.60 7.59 7.59 7.58 7.57 7.56 7.38 7.38 7.36 7.36 7.35 7.35 7.34 7.34 7.33 7.33 7.31 7.30 7.29 7.28 7.22 7.20 7.18 6.94 6.93 6.65 6.65 6.65 6.63 6.63 6.63 6.40 6.06 5.19 5.18 5.17 5.16 3.83 3.80 3.78 3.59
OH
OMe
OMe
OMe
PPh3
PF6
1H NMR (CDCl3), trans-13PF6
3.51
14.64
3.32
1.25
1.07
1.05
2.14
1.04
9.00
9.5 9.0 8.5 8.0 7.5 7.0 6.5 6.0 5.5 5.0 4.5 4.0 3.5 3.0 2.5
f1 (ppm)

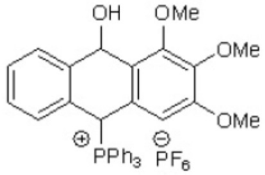

$^{13}C\{^{1}H\}$ NMR (MeOD), *trans*-**13**PF_6

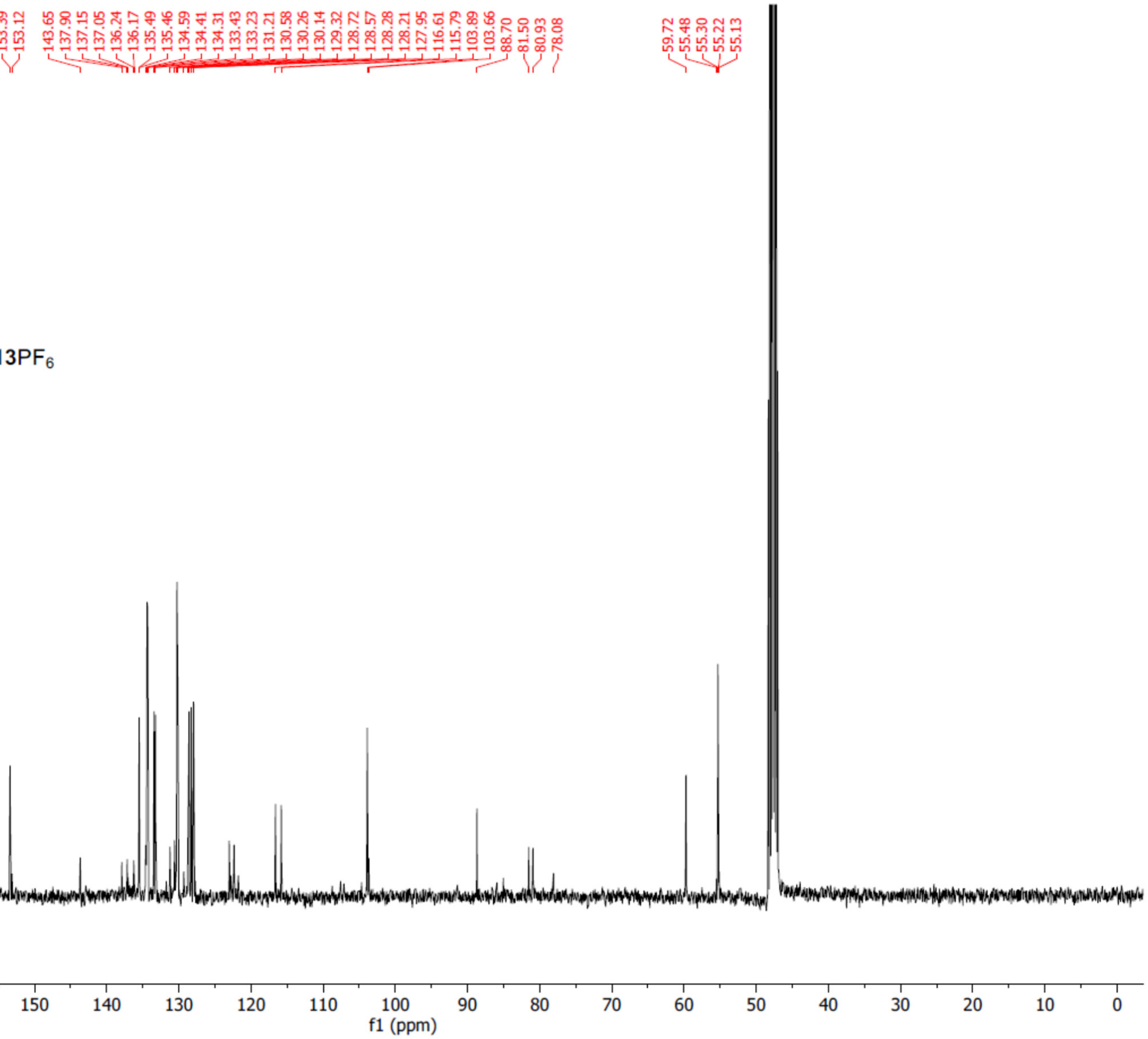

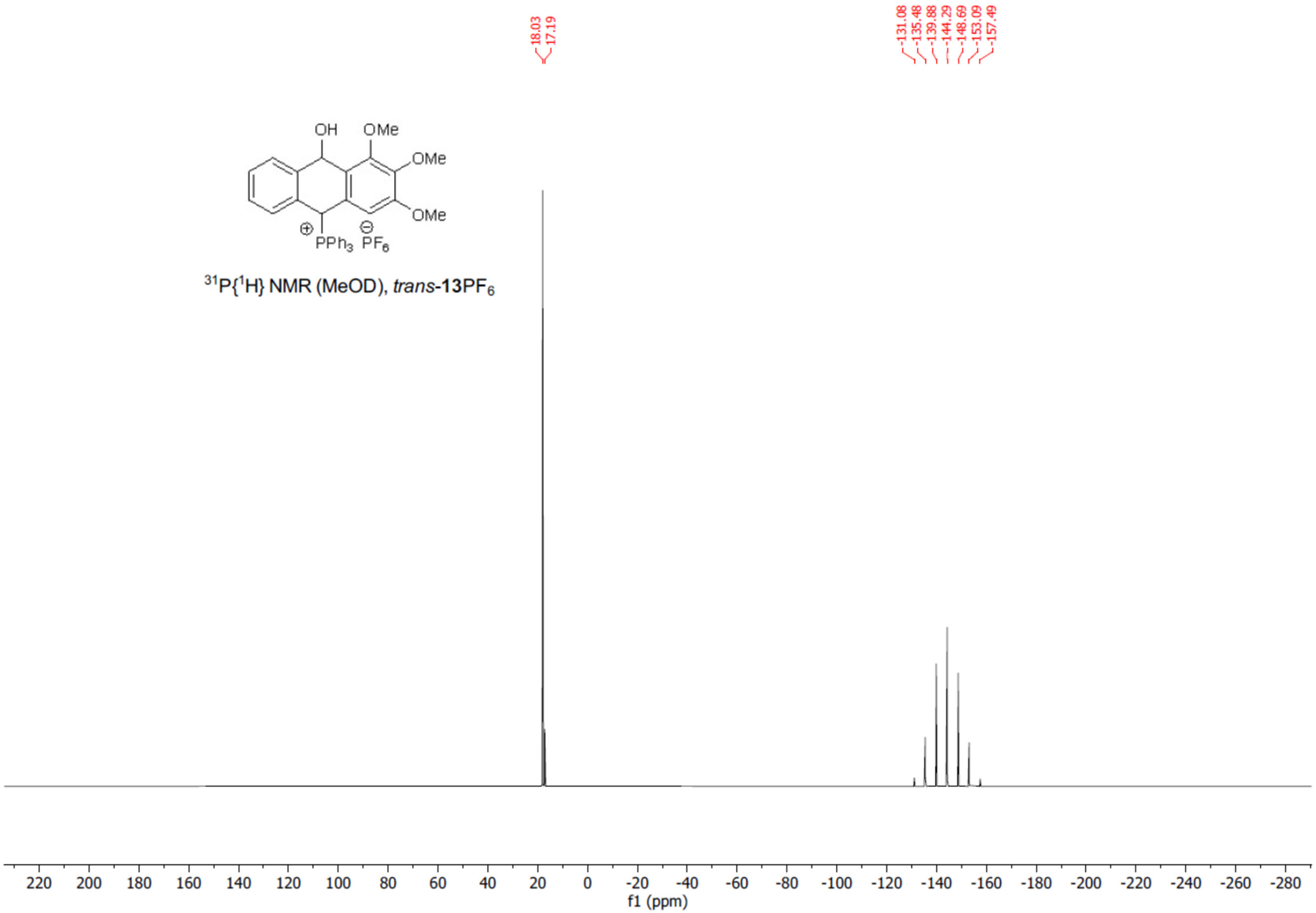
18.03
17.19
-131.08
-135.48
-139.88
-144.29
-148.69
-153.09
-157.49
OH
OMe
OMe
OMe
PPh3
PF6
$^{31}P\{^1H\}$ NMR (MeOD), *trans*-**13**PF_6
220 200 180 160 140 120 100 80 60 40 20 0 -20 -40 -60 -80 -100 -120 -140 -160 -180 -200 -220 -240 -260 -280
f1 (ppm)

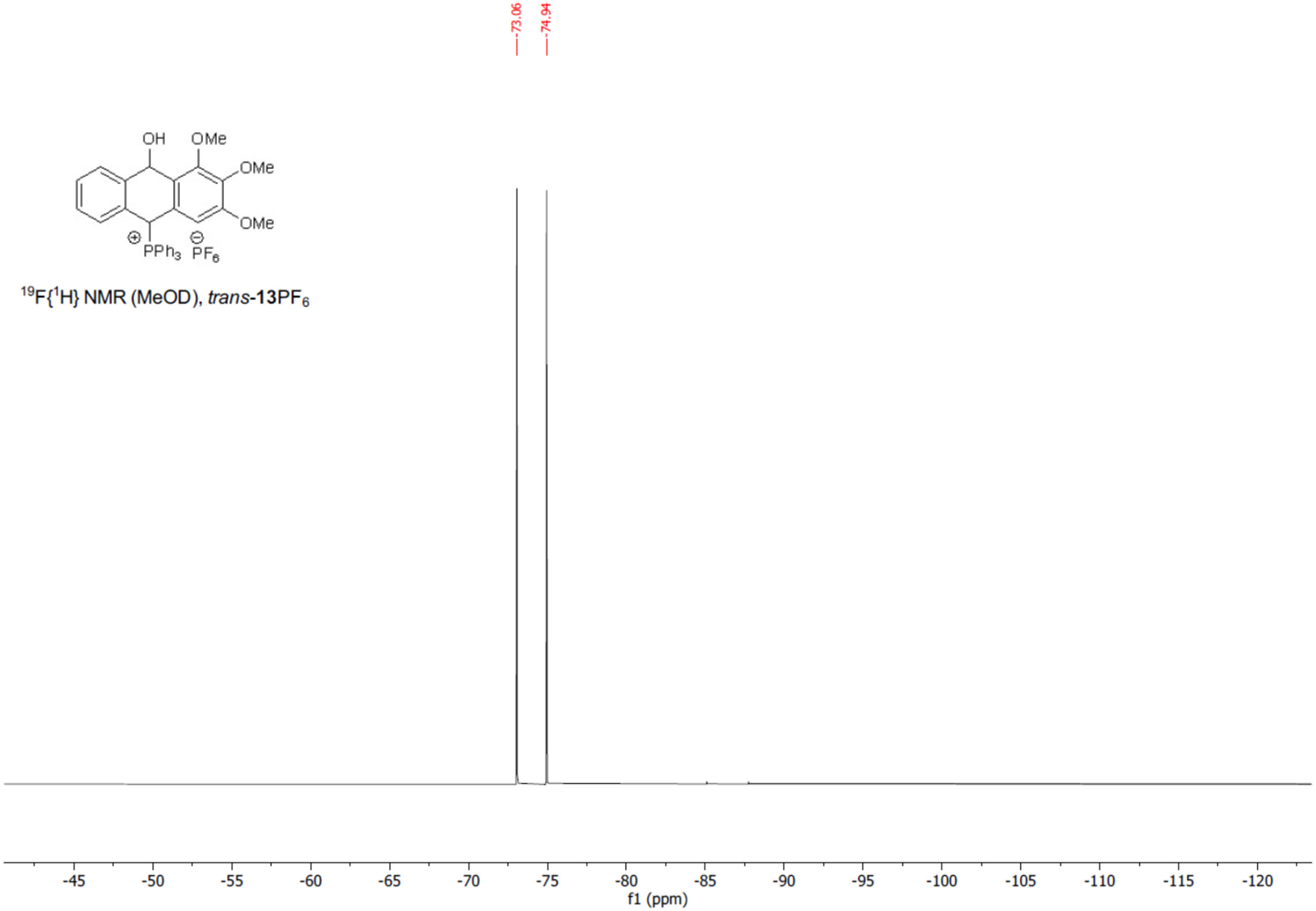

$^{19}F\{^1H\}$ NMR (MeOD), *trans*-**13**PF_6

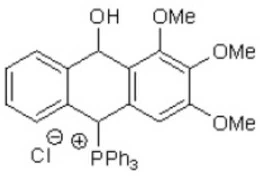

^{1}H NMR (CD_2Cl_2), *trans*-**13**Cl

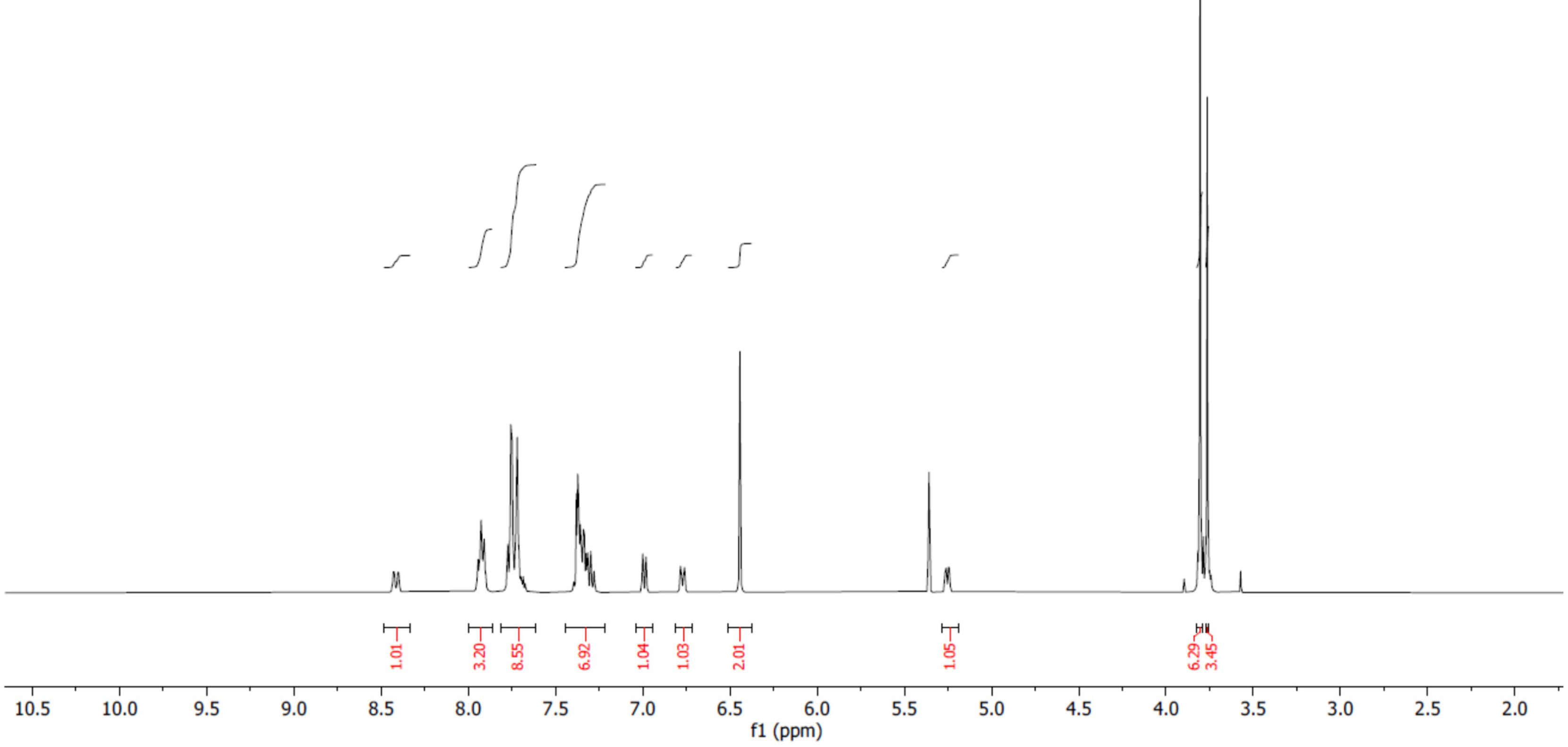

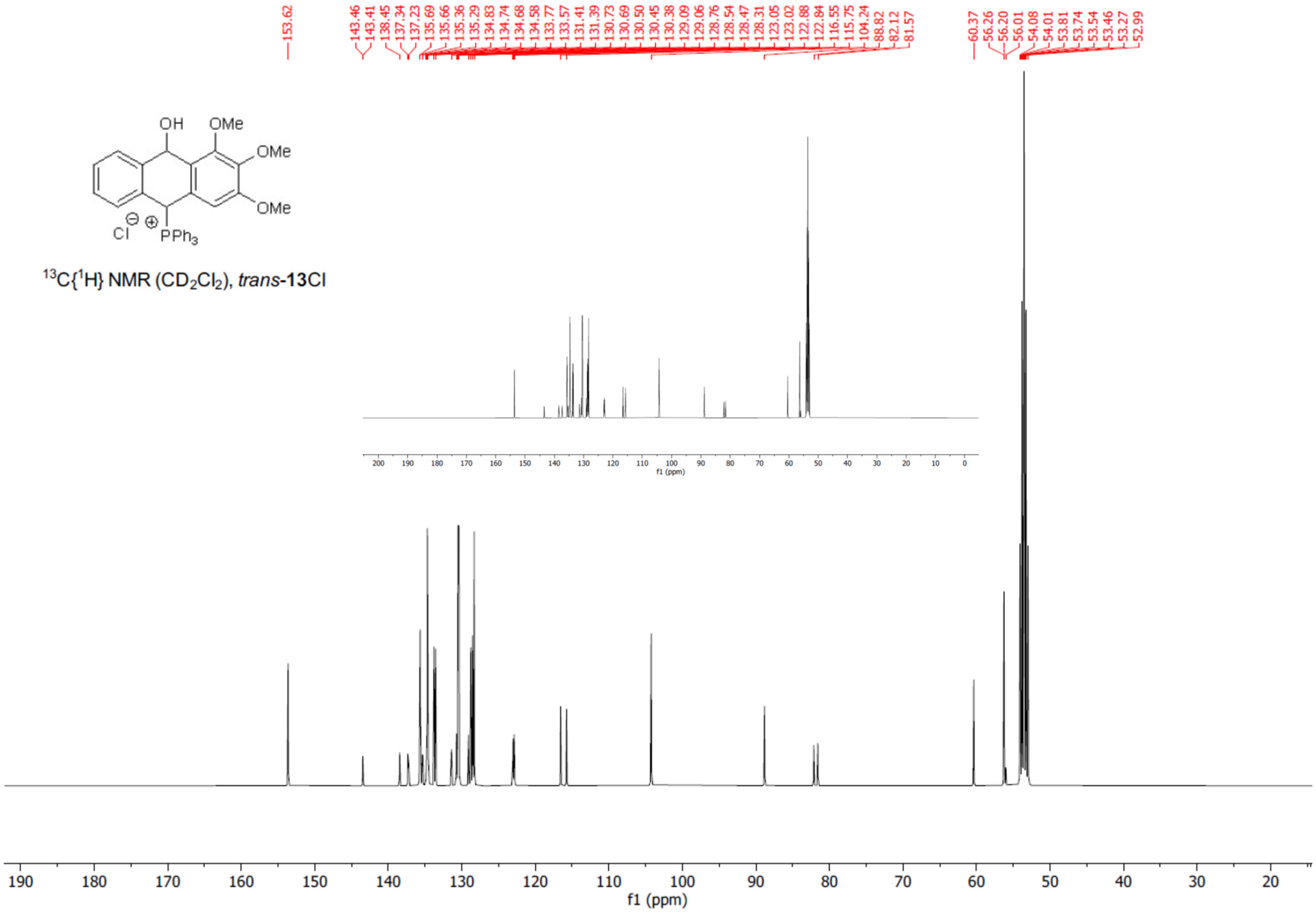

$^{13}C\{^{1}H\}$ NMR (CD_2Cl_2), *trans*-**13Cl**

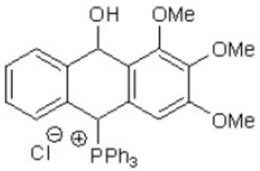

$^{31}P\{^1H\}$ NMR (CD_2Cl_2), *trans*-**13**Cl

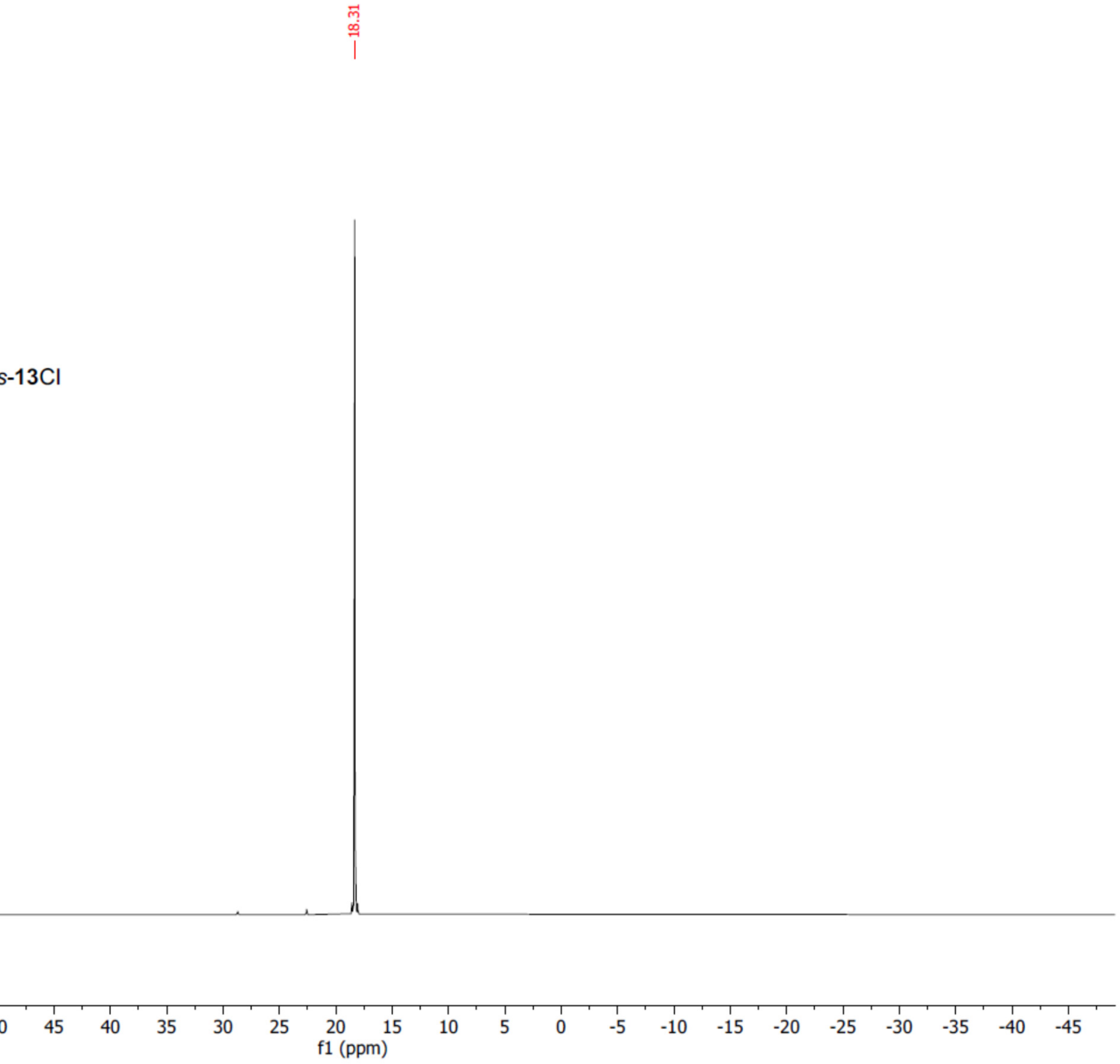

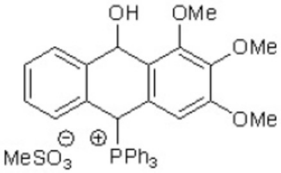

^{1}H NMR ($CDCl_3$), *trans*-**13**MsO

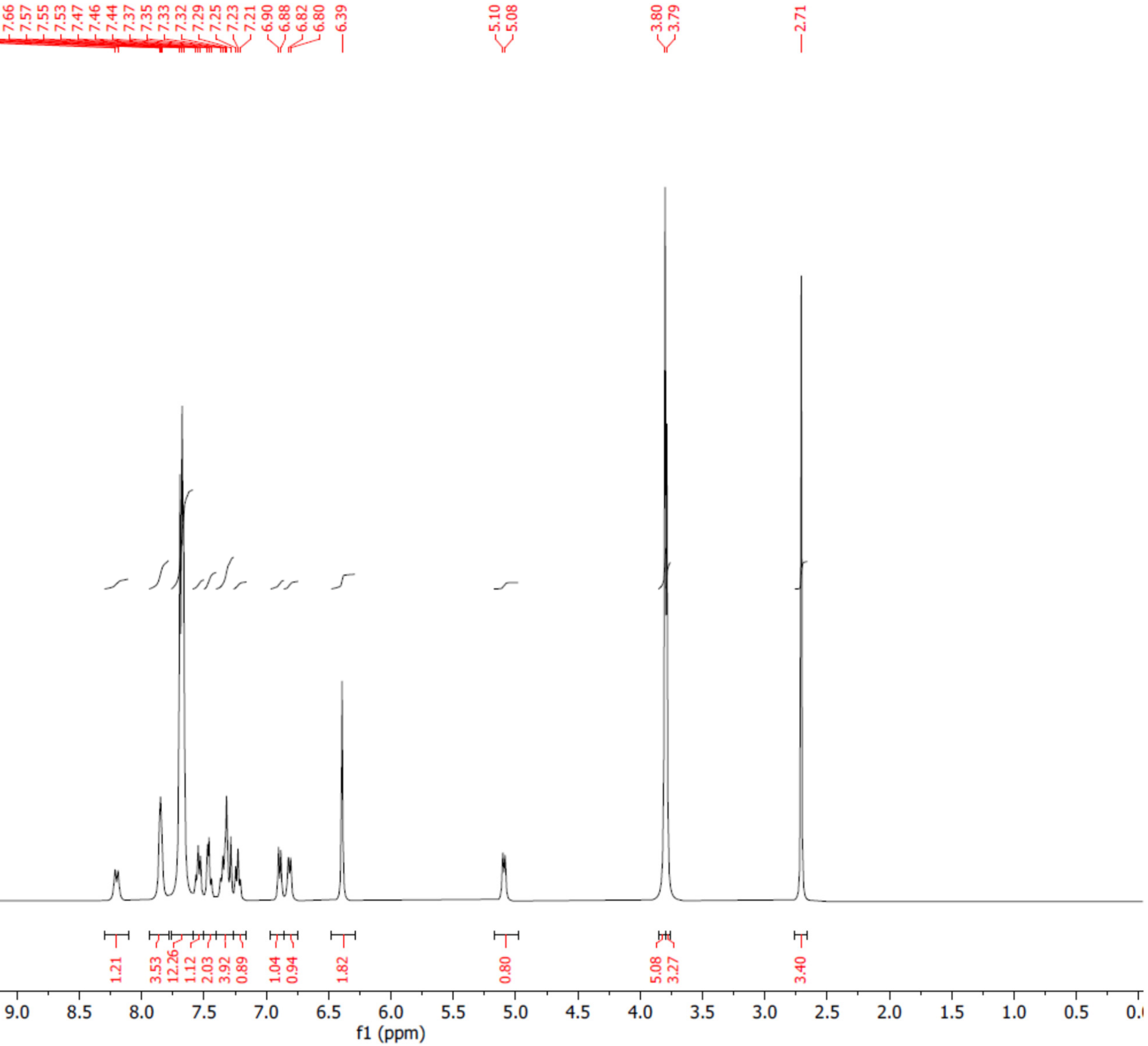

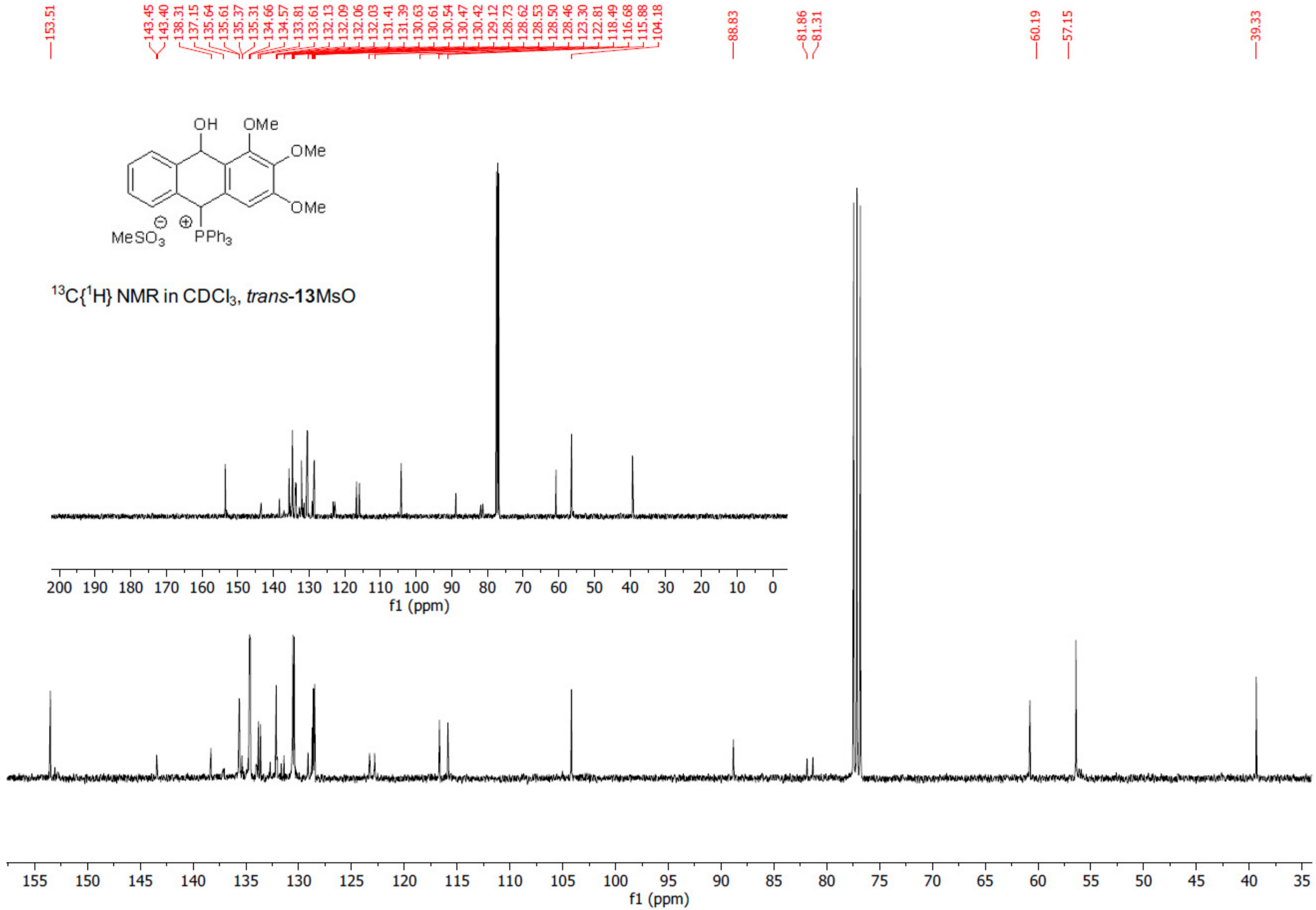
153.51
143.45
143.40
138.31
137.15
135.64
135.61
135.37
135.31
134.66
134.57
133.81
133.61
132.13
132.09
132.06
132.03
131.41
131.39
130.63
130.61
130.54
130.47
130.42
129.12
128.73
128.62
128.53
128.50
128.46
123.30
122.81
118.49
116.68
115.88
104.18
88.83
81.86
81.31
60.19
57.15
39.33
OH
OMe
OMe
OMe
MeSO3
PPh3
13C{1H} NMR in CDCl3, trans-13MsO
f1 (ppm)
f1 (ppm)

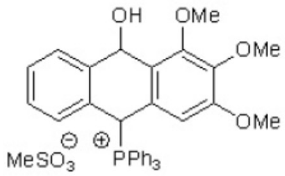

$^{31}P\{^1H\}$ NMR in $CDCl_3$, *trans*-**13**MsO

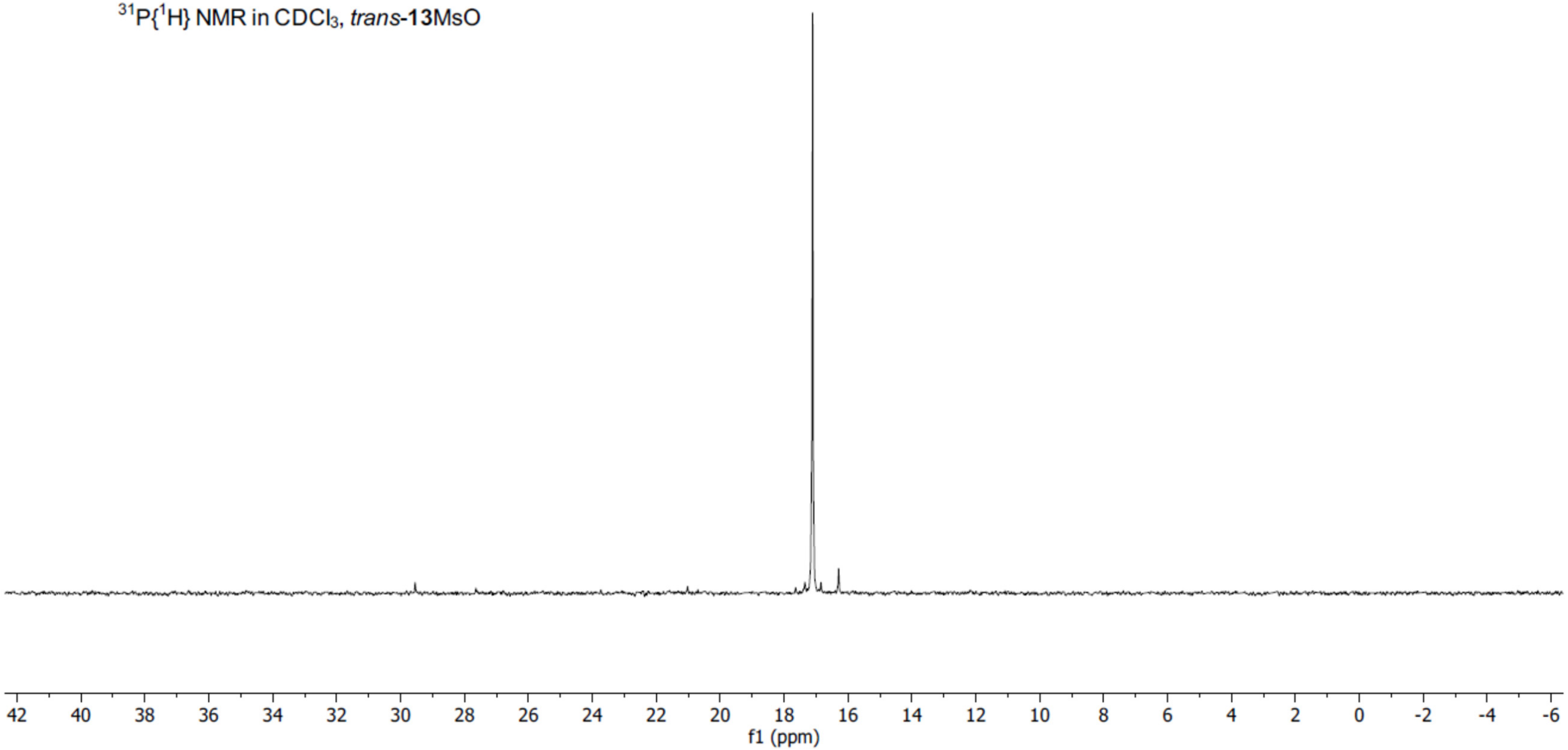

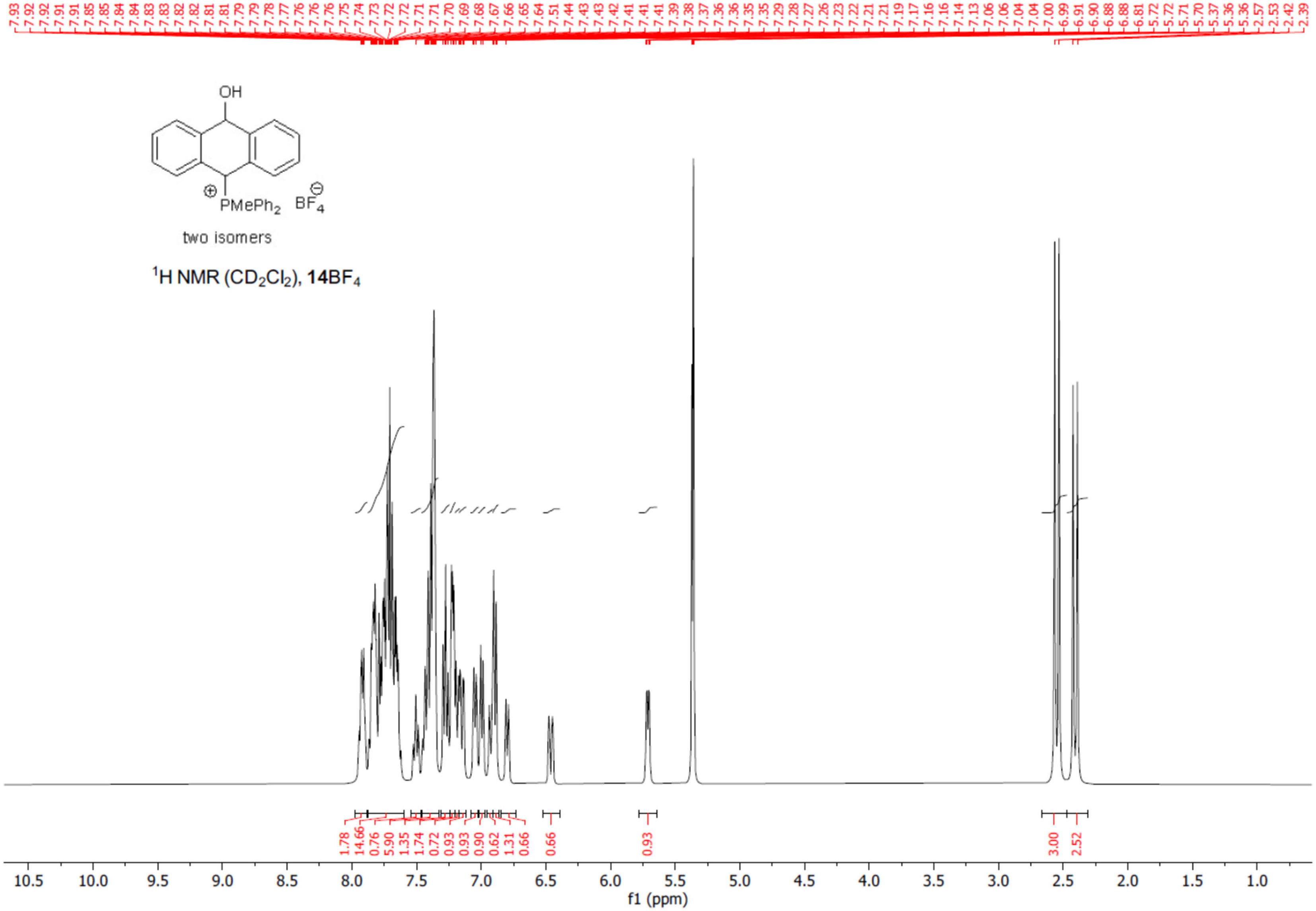
OH
PMePh2 BF4
two isomers
1H NMR (CD2Cl2), 14BF4
f1 (ppm)

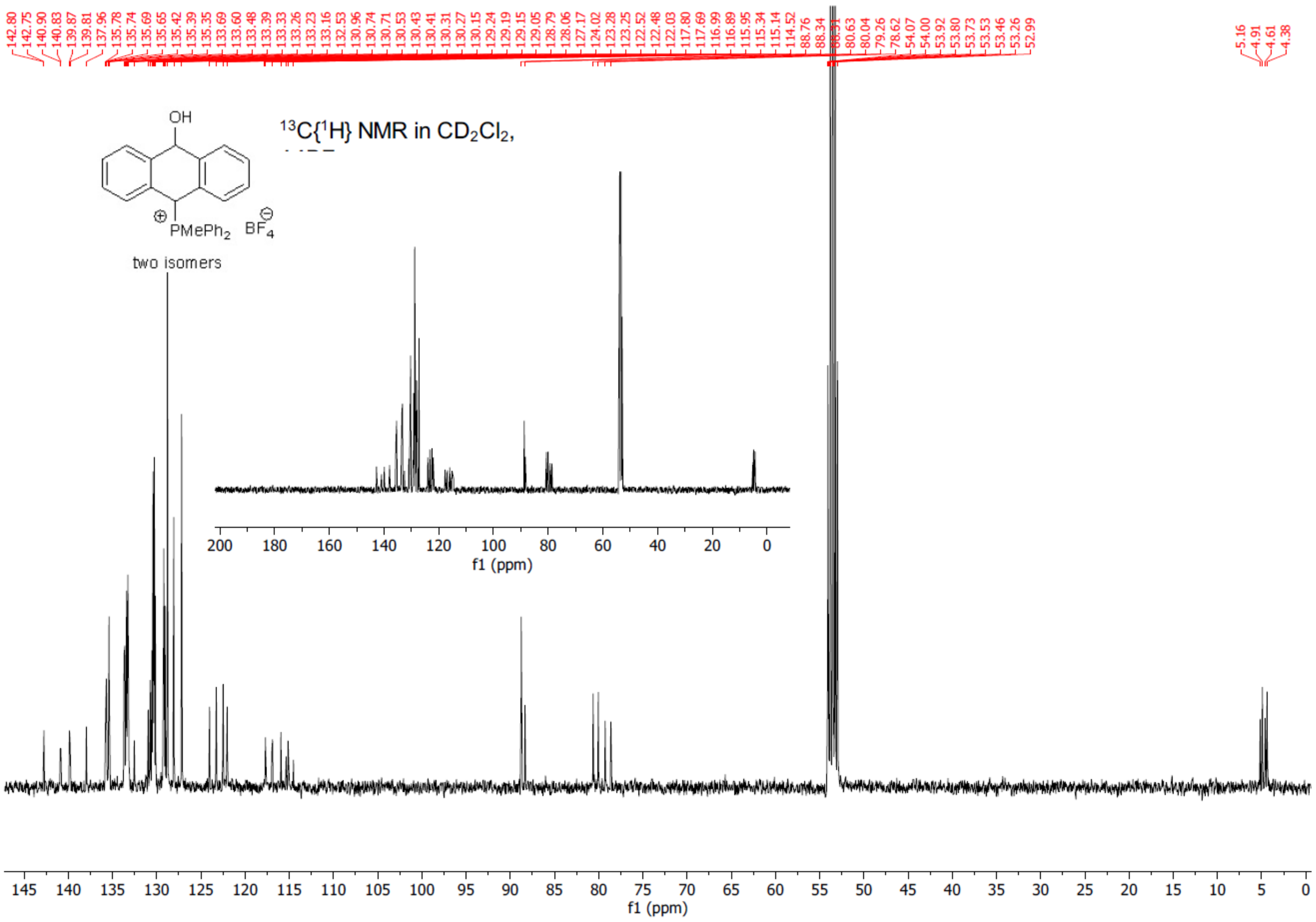
OH
PMePh2 BF4
two isomers
13C{1H} NMR in CD2Cl2,
f1 (ppm)

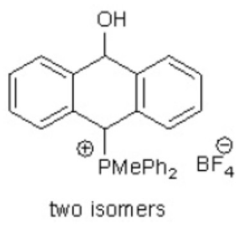

$^{31}P\{^1H\}$ NMR in CD_2Cl_2, **14**BF_4

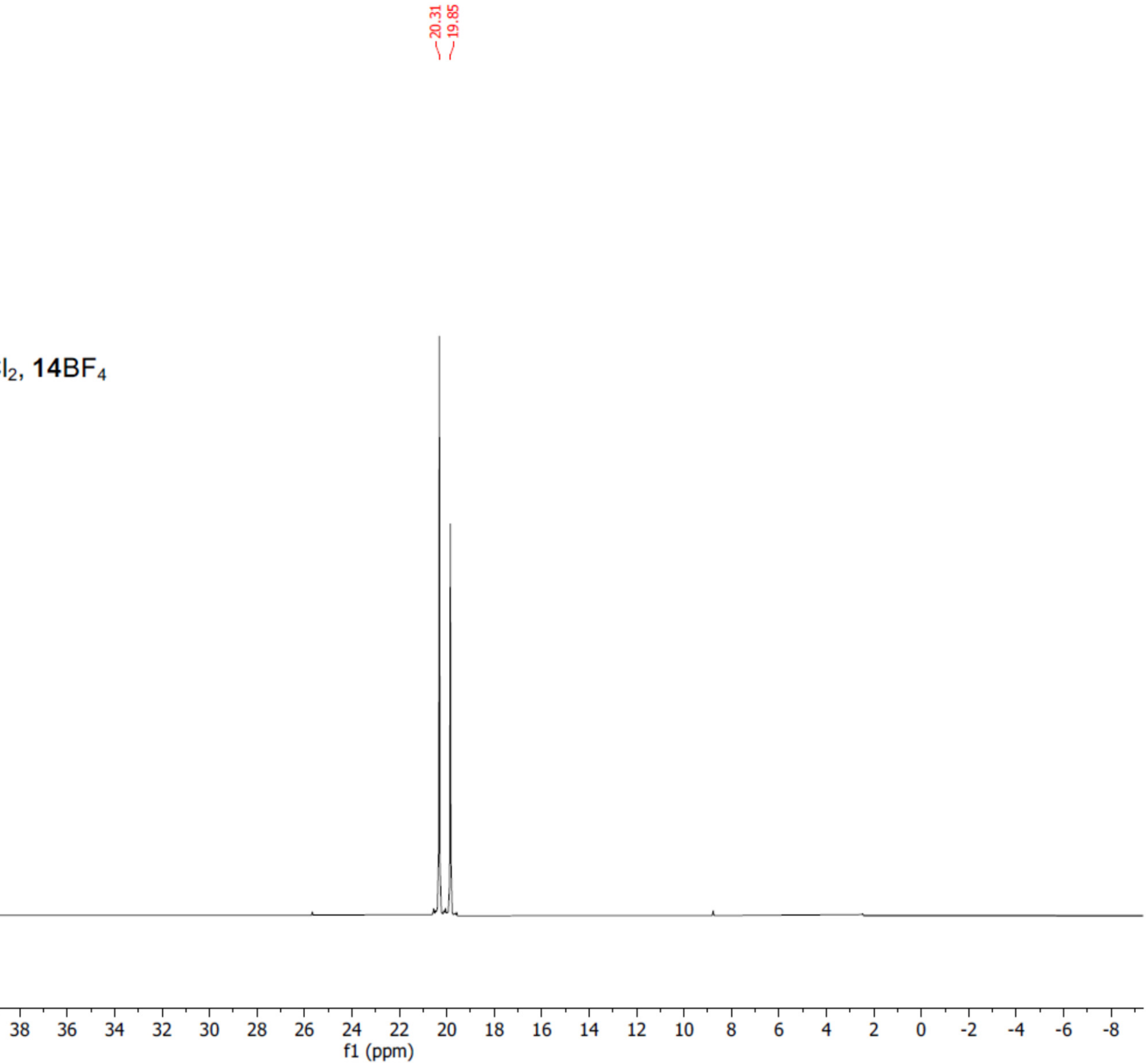

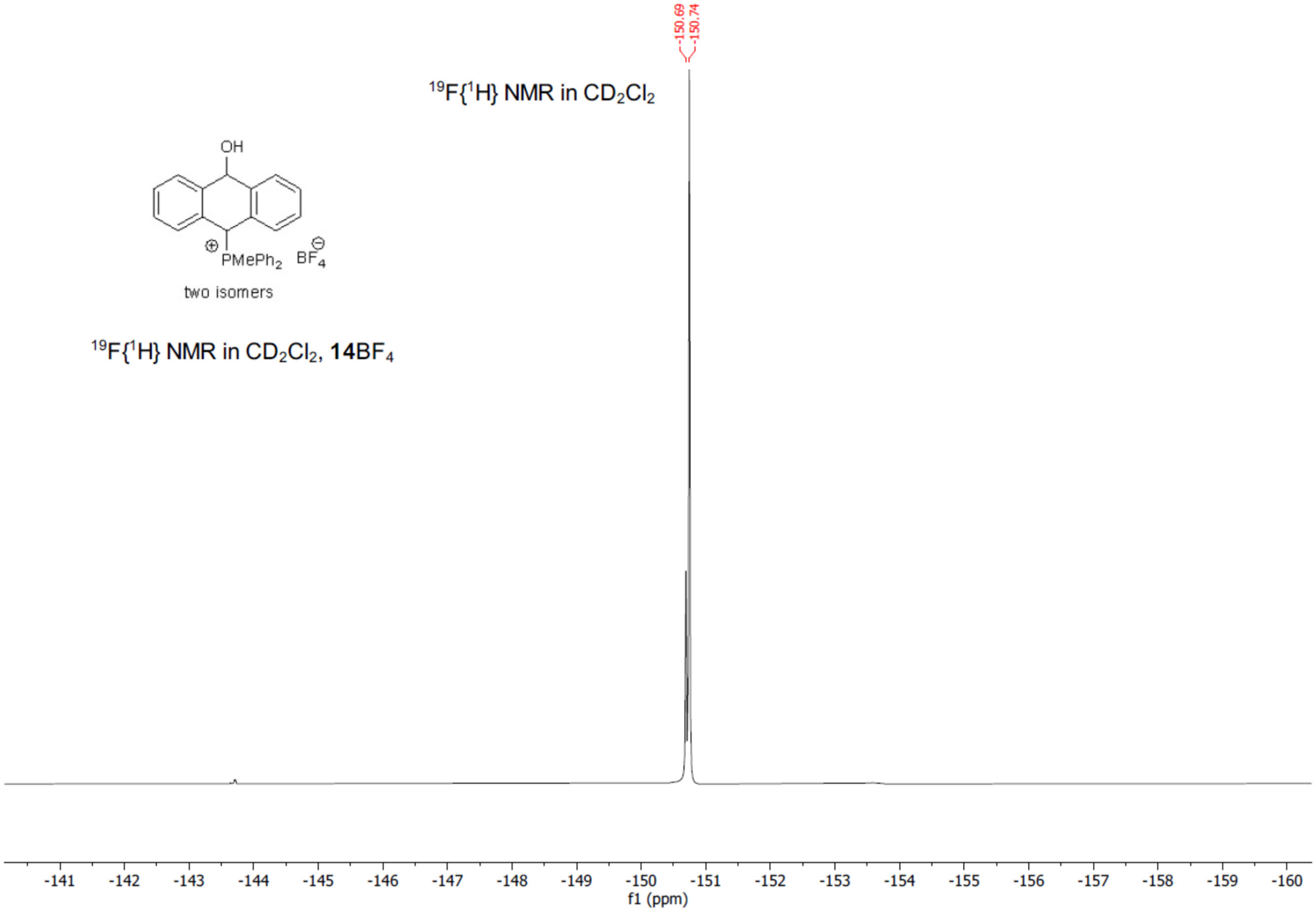
-150.69
-150.74
$^{19}F\{^1H\}$ NMR in CD_2Cl_2
OH
PMePh$_2$ BF$_4$
two isomers
$^{19}F\{^1H\}$ NMR in CD_2Cl_2, **14**BF$_4$
-141 -142 -143 -144 -145 -146 -147 -148 -149 -150 -151 -152 -153 -154 -155 -156 -157 -158 -159 -160
f1 (ppm)

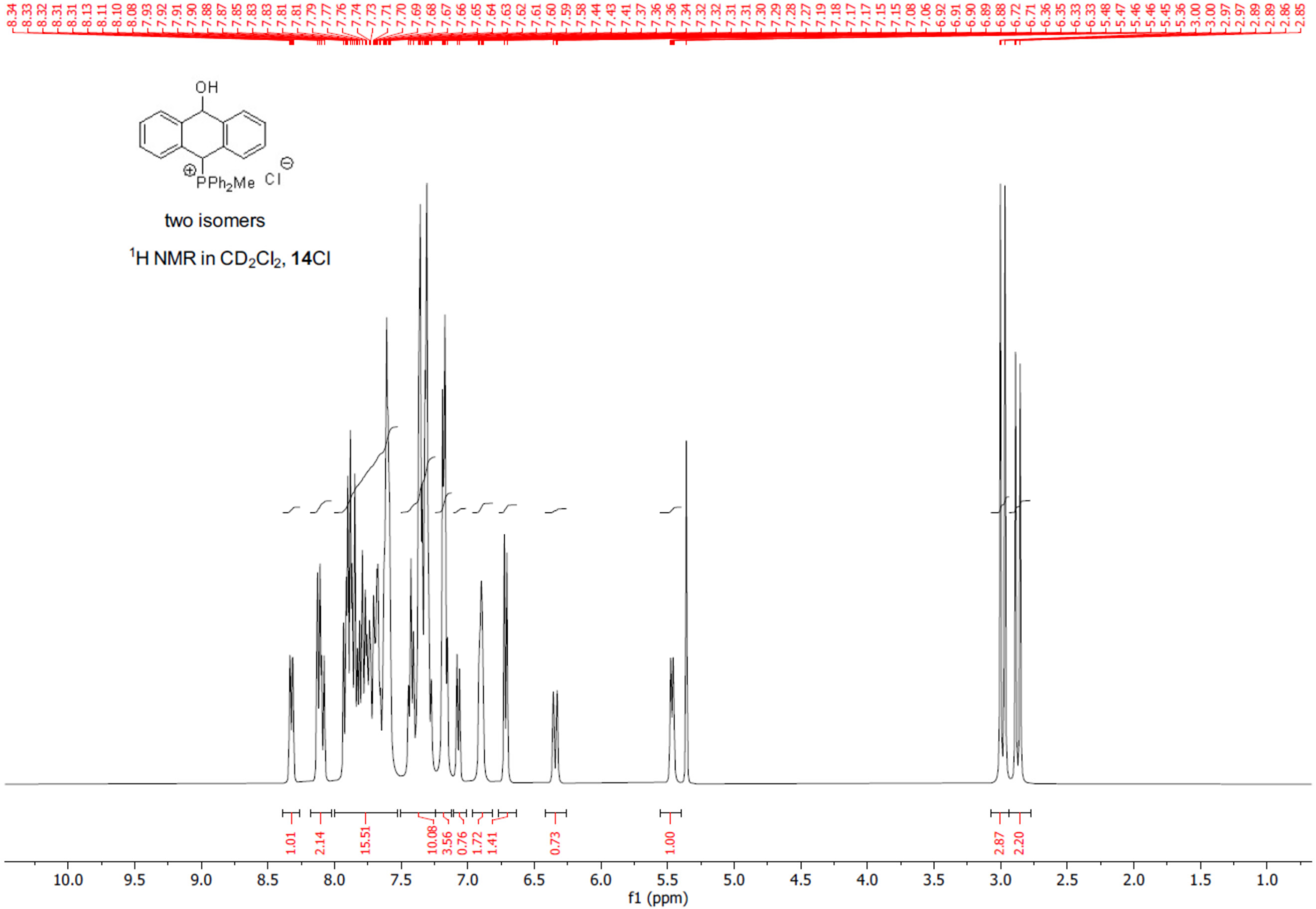
8.34 8.33 8.32 8.31 8.31 8.13 8.11 8.10 8.08 7.93 7.92 7.91 7.90 7.88 7.87 7.85 7.83 7.83 7.81 7.81 7.79 7.77 7.76 7.74 7.73 7.71 7.70 7.69 7.68 7.67 7.66 7.65 7.64 7.63 7.62 7.61 7.60 7.59 7.58 7.44 7.43 7.41 7.37 7.36 7.36 7.34 7.32 7.32 7.31 7.31 7.30 7.29 7.28 7.27 7.19 7.18 7.17 7.17 7.15 7.15 7.08 7.06 6.92 6.91 6.90 6.89 6.88 6.72 6.71 6.36 6.35 6.33 6.33 5.48 5.47 5.46 5.46 5.45 5.36 3.00 3.00 2.97 2.97 2.89 2.89 2.86 2.85
OH
PPh2Me
Cl
two isomers
1H NMR in CD2Cl2, 14Cl
1.01 2.14 15.51 10.08 3.56 0.76 1.72 1.41 0.73 1.00 2.87 2.20
10.0 9.5 9.0 8.5 8.0 7.5 7.0 6.5 6.0 5.5 5.0 4.5 4.0 3.5 3.0 2.5 2.0 1.5 1.0
f1 (ppm)

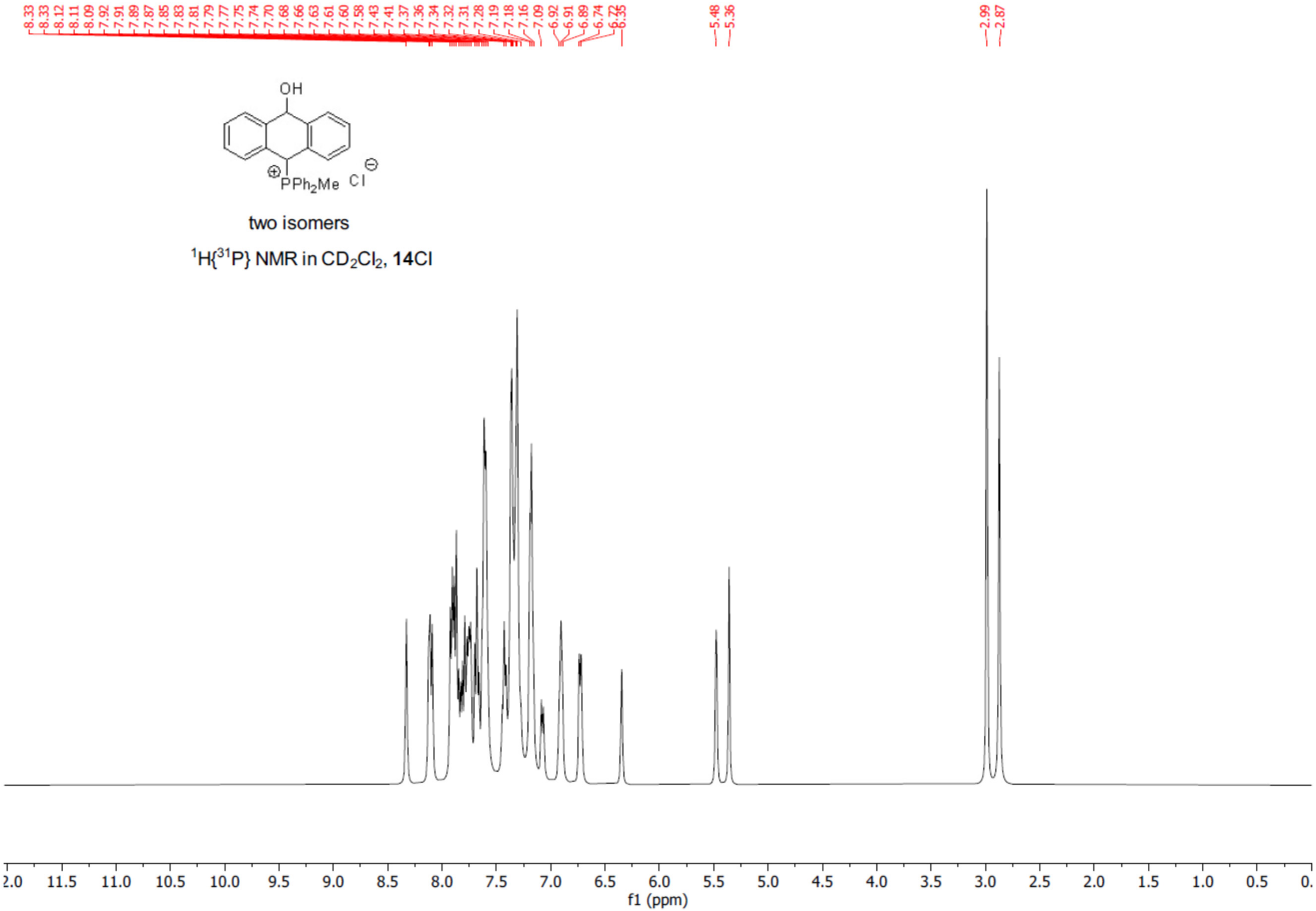
8.33
8.33
8.12
8.11
8.09
7.92
7.91
7.89
7.87
7.85
7.83
7.81
7.79
7.77
7.75
7.74
7.70
7.68
7.66
7.63
7.61
7.60
7.58
7.43
7.41
7.37
7.36
7.34
7.32
7.31
7.28
7.19
7.18
7.16
7.09
6.92
6.91
6.89
6.74
6.72
6.35
5.48
5.36
2.99
2.87
OH
PPh2Me
Cl
two isomers
1H{31P} NMR in CD2Cl2, 14Cl
f1 (ppm)

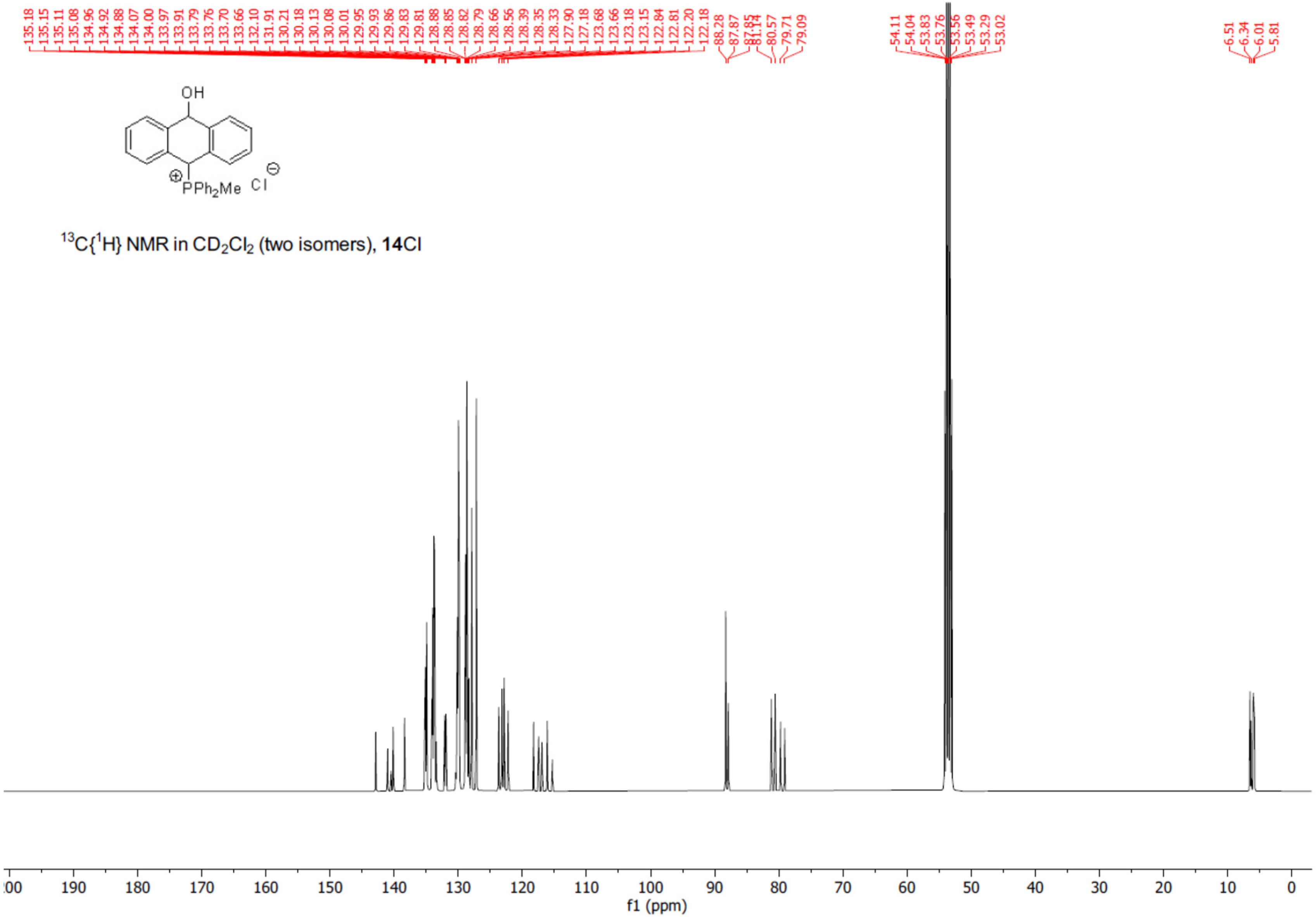
135.18 135.15 135.11 135.08 134.96 134.92 134.88 134.07 134.00 133.97 133.91 133.79 133.76 133.70 133.66 132.10 131.91 130.21 130.18 130.13 130.08 130.01 129.95 129.93 129.86 129.83 129.81 128.88 128.85 128.82 128.79 128.66 128.56 128.39 128.35 128.33 127.90 127.18 123.68 123.66 123.18 123.15 122.84 122.81 122.20 122.18 88.28 87.87 87.85 81.14 80.57 79.71 79.09
54.11 54.04 53.83 53.76 53.56 53.49 53.29 53.02
6.51 6.34 6.01 5.81
OH
PPh2Me Cl
$^{13}C\{^1H\}$ NMR in CD_2Cl_2 (two isomers), **14**Cl
200 190 180 170 160 150 140 130 120 110 100 90 80 70 60 50 40 30 20 10 0
f1 (ppm)

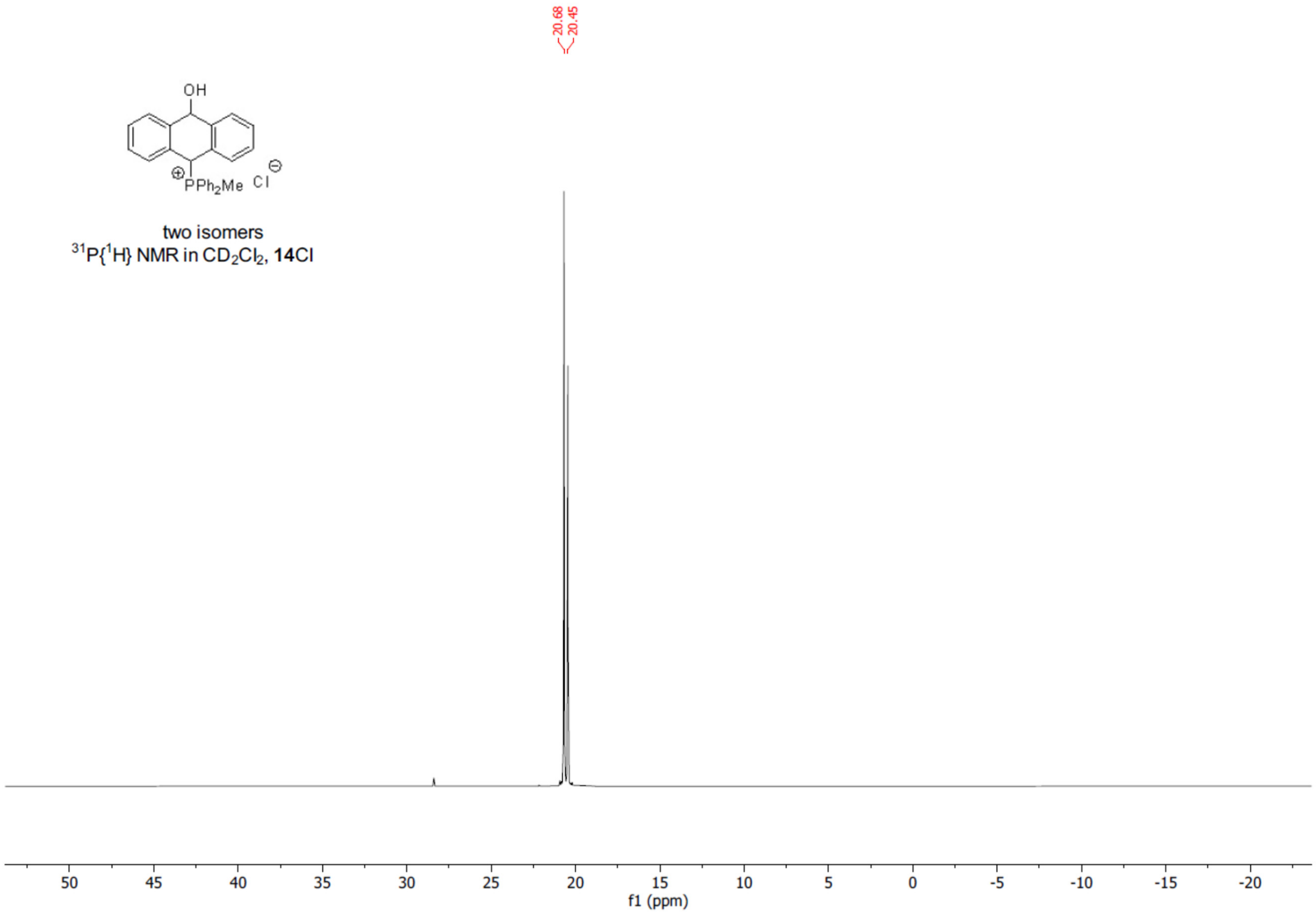
20.68
20.45
OH
PPh2Me
Cl
two isomers
31P{1H} NMR in CD2Cl2, 14Cl
50
45
40
35
30
25
20
15
10
5
0
-5
-10
-15
-20
f1 (ppm)

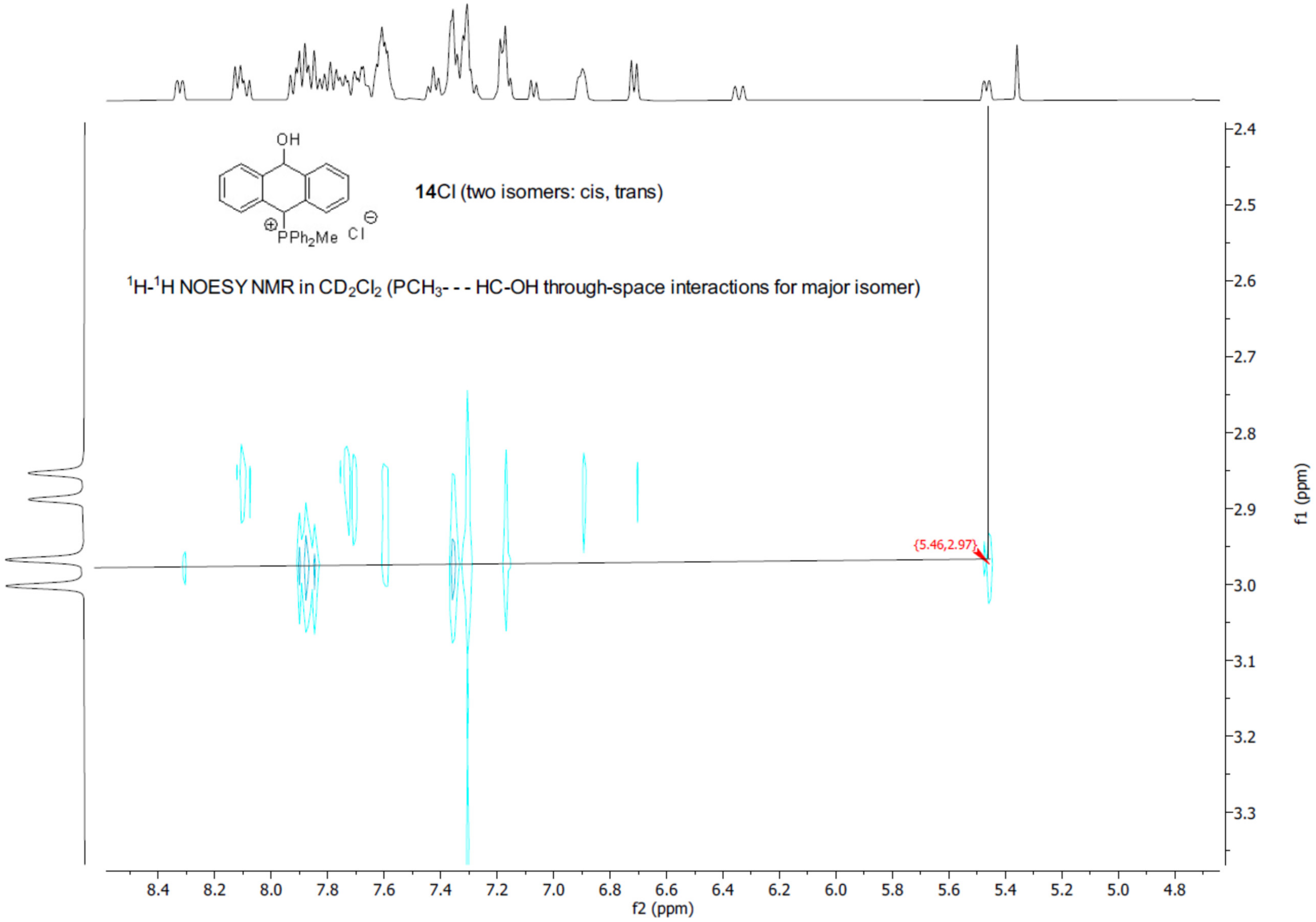
OH
PPh2Me Cl
14Cl (two isomers: cis, trans)
1H-1H NOESY NMR in CD2Cl2 (PCH3- - - HC-OH through-space interactions for major isomer)
{5.46,2.97}
f2 (ppm)
f1 (ppm)

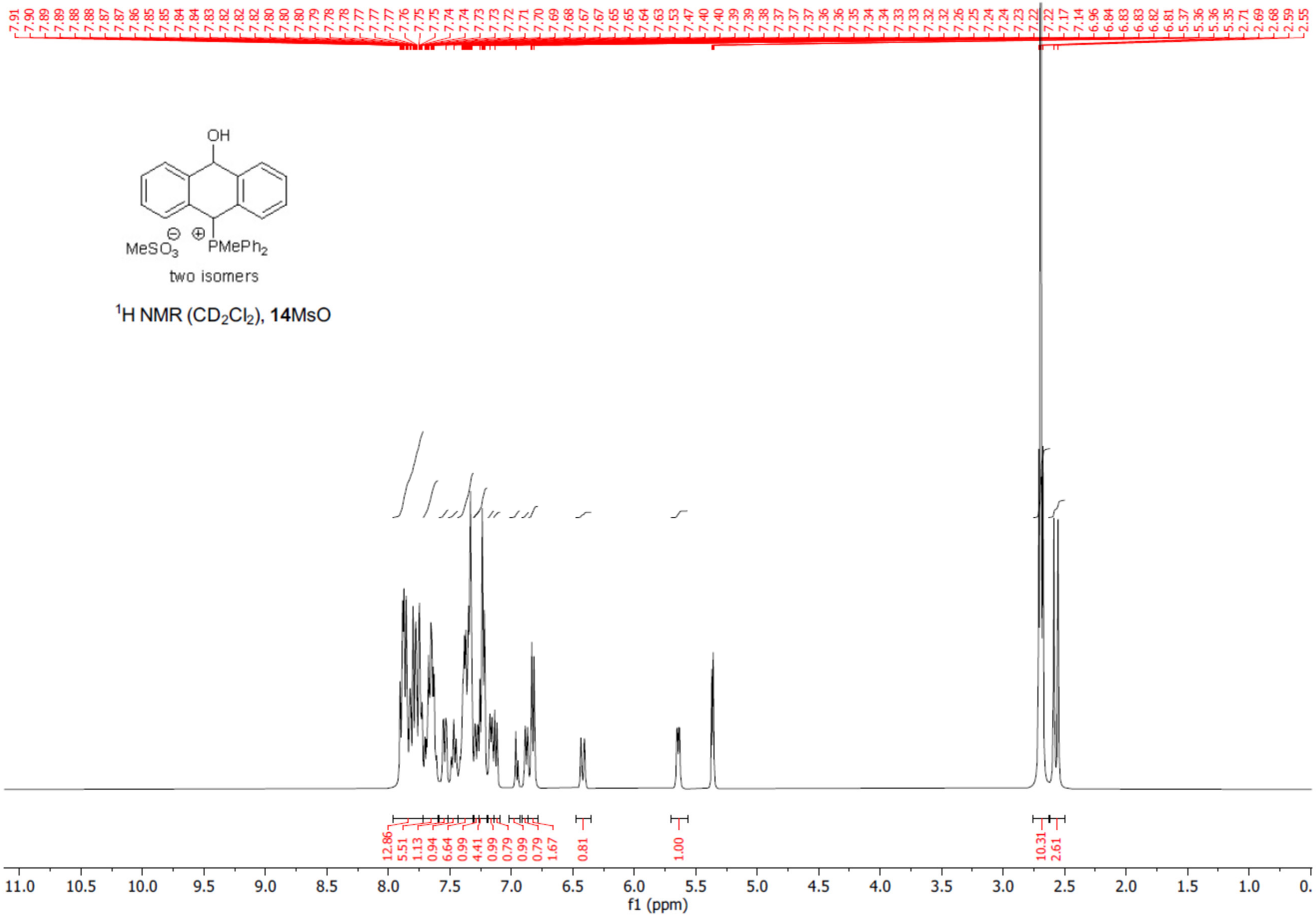
7.91 7.90 7.89 7.88 7.88 7.87 7.87 7.86 7.85 7.85 7.84 7.84 7.83 7.82 7.82 7.82 7.80 7.80 7.79 7.78 7.78 7.77 7.77 7.77 7.76 7.75 7.75 7.74 7.74 7.73 7.73 7.72 7.71 7.70 7.69 7.68 7.67 7.67 7.65 7.65 7.64 7.63 7.53 7.47 7.40 7.40 7.39 7.39 7.38 7.37 7.37 7.37 7.36 7.36 7.35 7.34 7.34 7.33 7.33 7.32 7.32 7.26 7.25 7.24 7.24 7.23 7.22 7.22 7.17 7.14 6.96 6.84 6.83 6.83 6.82 6.81 5.37 5.36 5.35 2.71 2.69 2.68 2.59 2.55
OH
MeSO3 ⊖ ⊕ PMePh2
two isomers
1H NMR (CD2Cl2), 14MsO
12.86 5.51 1.13 0.94 6.64 0.99 4.41 0.99 0.79 0.99 0.79 1.67 0.81 1.00 10.31 2.61
11.0 10.5 10.0 9.5 9.0 8.5 8.0 7.5 7.0 6.5 6.0 5.5 5.0 4.5 4.0 3.5 3.0 2.5 2.0 1.5 1.0 0.
f1 (ppm)

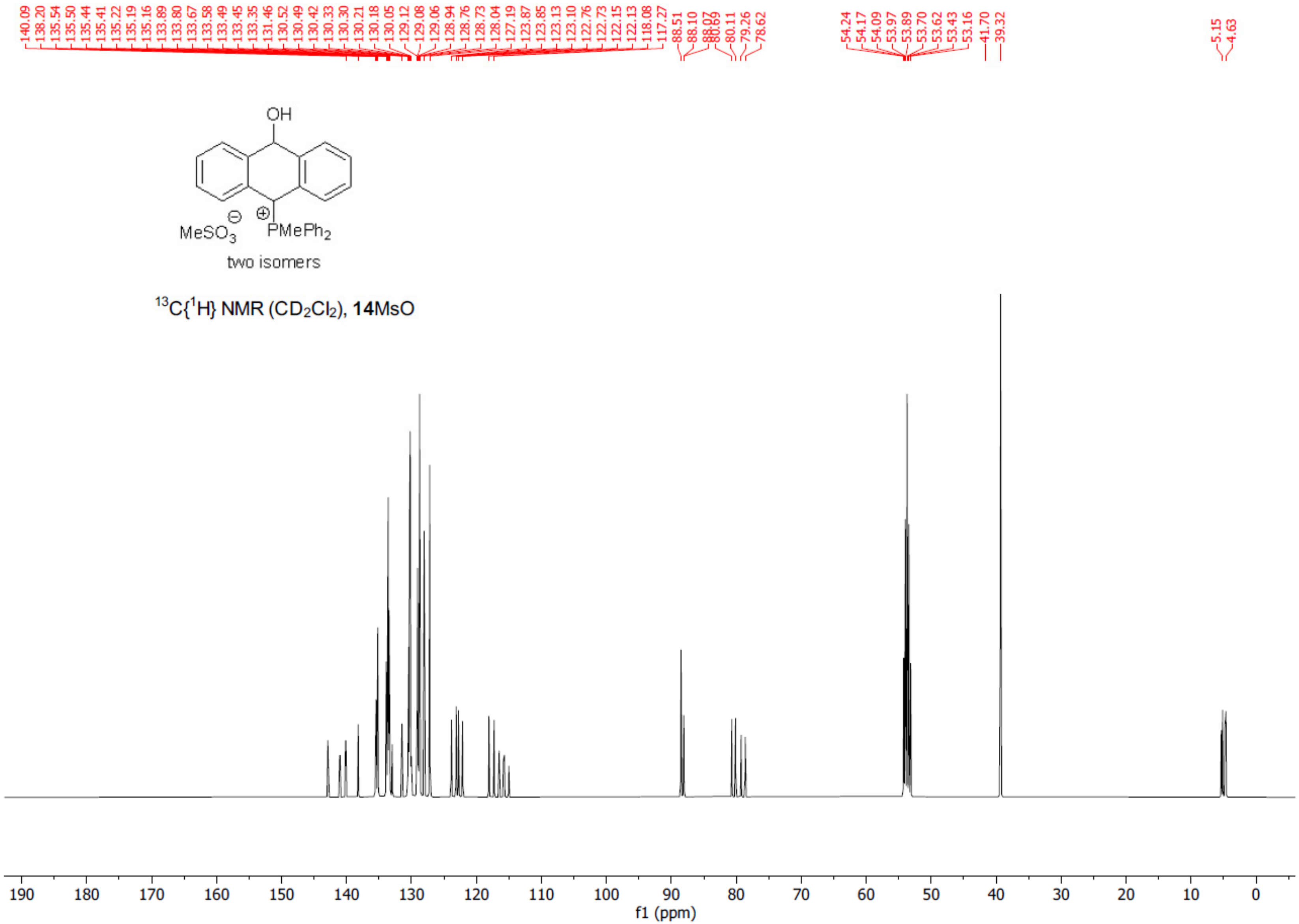
140.09
138.20
135.54
135.50
135.44
135.41
135.22
135.19
135.16
133.89
133.80
133.67
133.58
133.49
133.45
133.35
131.46
130.52
130.49
130.42
130.33
130.30
130.21
130.18
130.05
129.12
129.08
129.06
128.94
128.76
128.73
128.04
127.19
123.87
123.85
123.13
123.10
122.76
122.73
122.15
122.13
118.08
117.27
88.51
88.10
80.11
79.26
78.62
54.24
54.17
54.09
53.97
53.89
53.70
53.62
53.43
53.16
41.70
39.32
5.15
4.63
OH
MeSO3
PMePh2
two isomers
13C{1H} NMR (CD2Cl2), 14MsO
190 180 170 160 150 140 130 120 110 100 90 80 70 60 50 40 30 20 10 0
f1 (ppm)

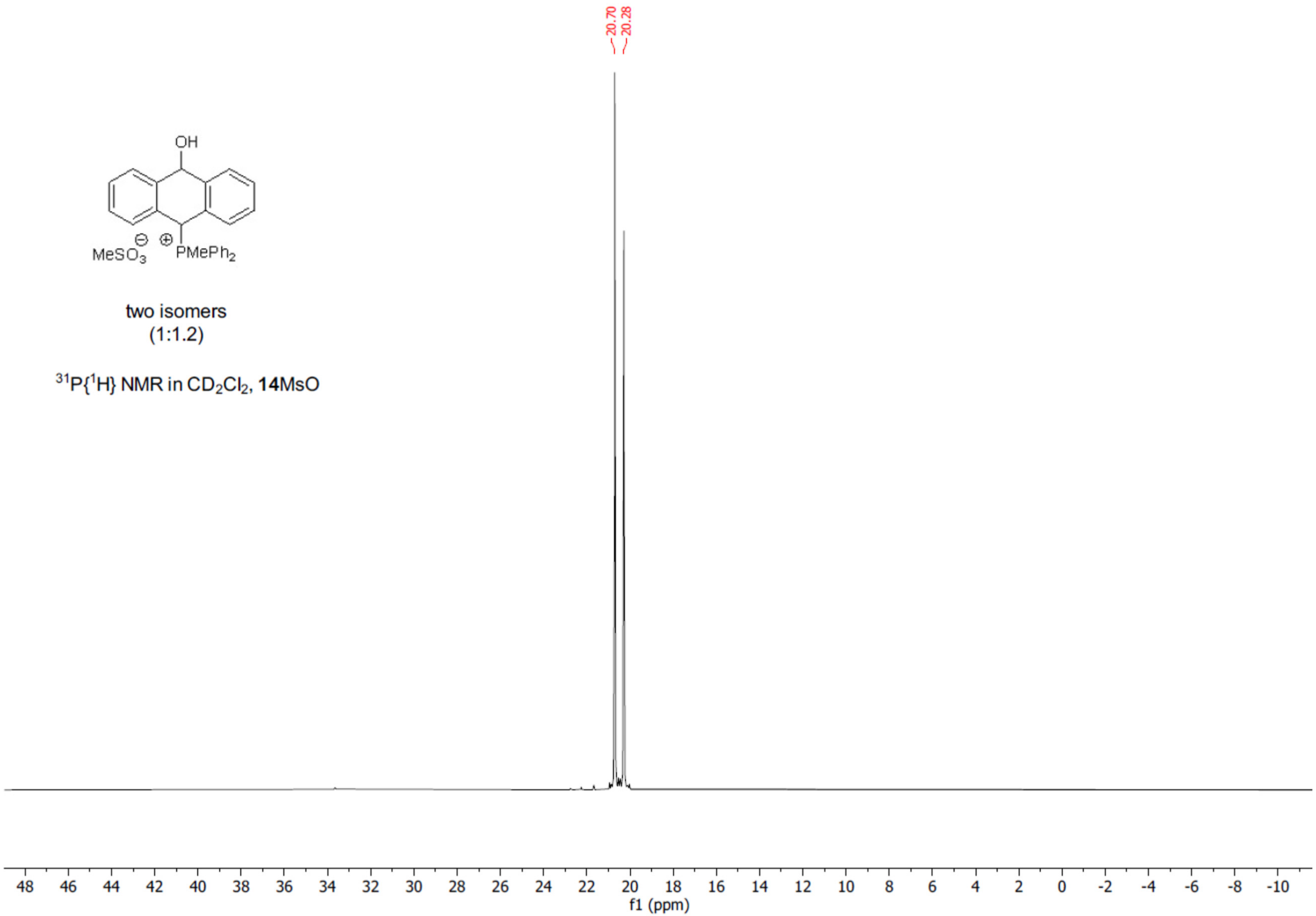
20.70
20.28
OH
MeSO3
PMePh2
two isomers
(1:1.2)
31P{1H} NMR in CD2Cl2, 14MsO
48 46 44 42 40 38 36 34 32 30 28 26 24 22 20 18 16 14 12 10 8 6 4 2 0 -2 -4 -6 -8 -10
f1 (ppm)

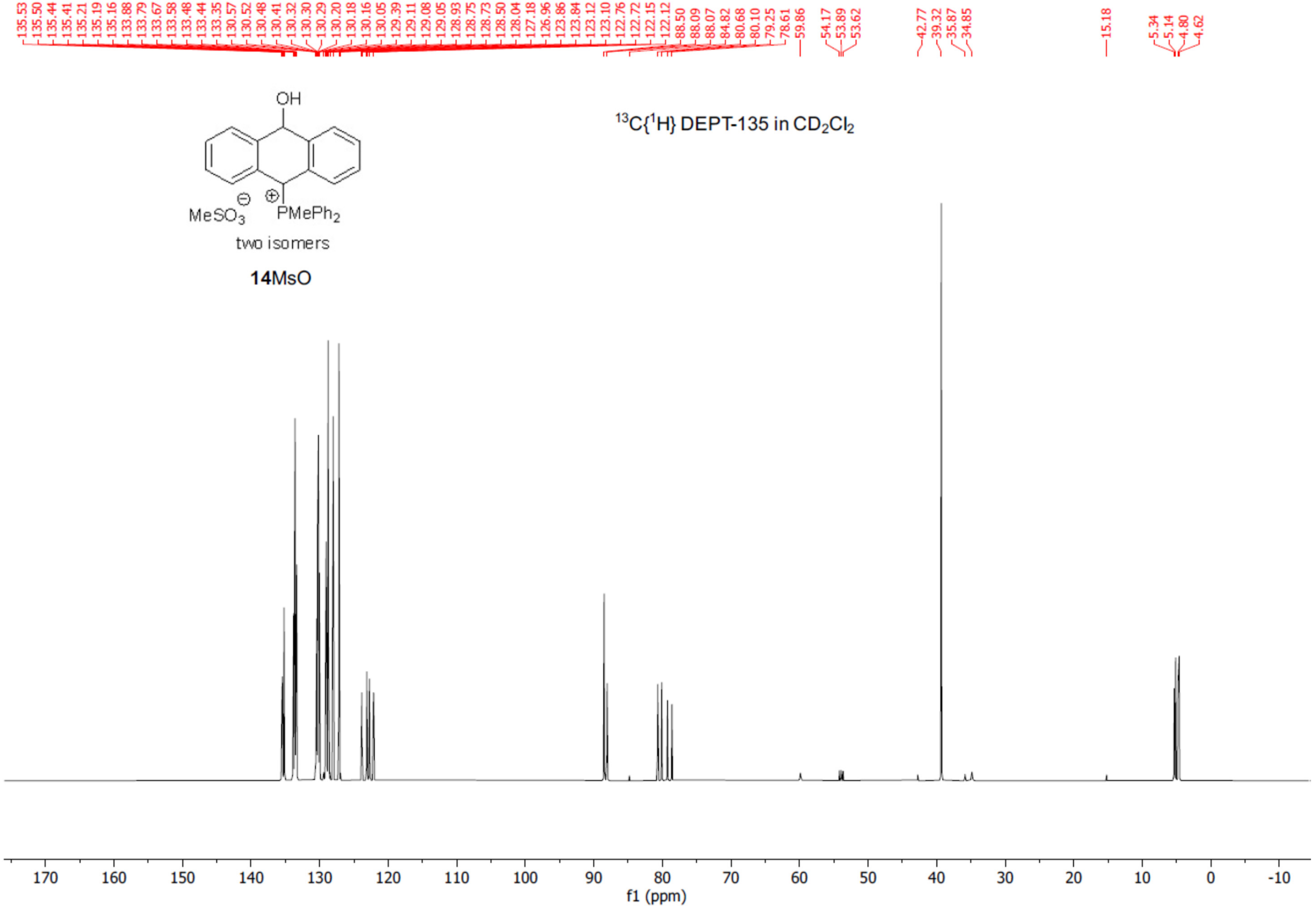
135.53
135.50
135.44
135.41
135.21
135.19
135.16
133.88
133.79
133.67
133.58
133.48
133.44
133.35
130.57
130.52
130.48
130.41
130.32
130.30
130.29
130.20
130.18
130.16
130.05
129.39
129.11
129.08
129.05
128.93
128.75
128.73
128.50
128.04
127.18
126.96
123.86
123.84
123.12
123.10
122.76
122.72
122.15
122.12
88.50
88.09
88.07
84.82
80.68
80.10
79.25
78.61
59.86
54.17
53.89
53.62
42.77
39.32
35.87
34.85
15.18
5.34
5.14
4.80
4.62
OH
MeSO3
PMePh2
two isomers
14MsO
13C{1H} DEPT-135 in CD2Cl2
170
160
150
140
130
120
110
100
90
80
70
60
50
40
30
20
10
0
-10
f1 (ppm)

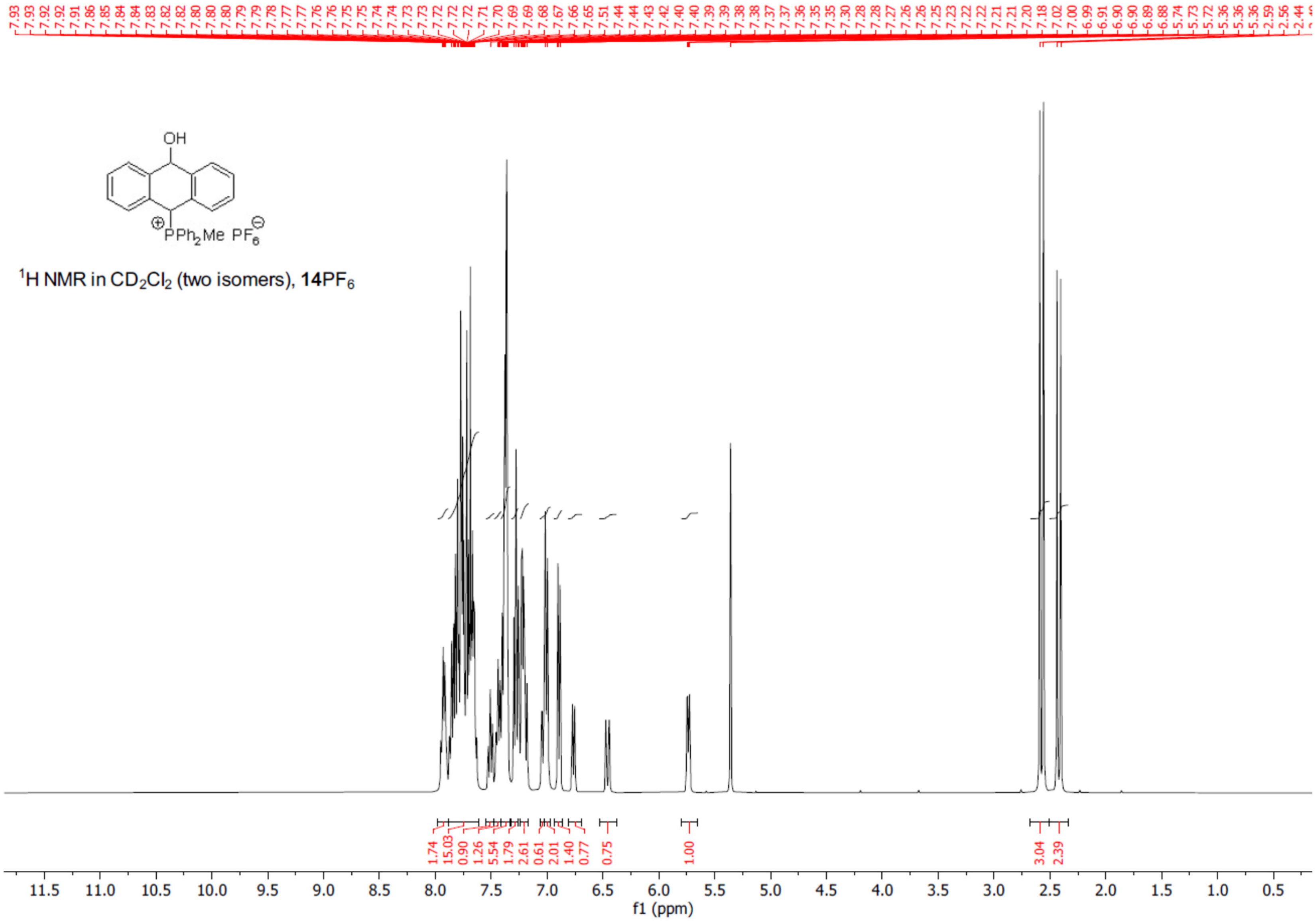

OH
PPh2Me PF6
1H NMR in CD2Cl2 (two isomers), 14PF6
f1 (ppm)

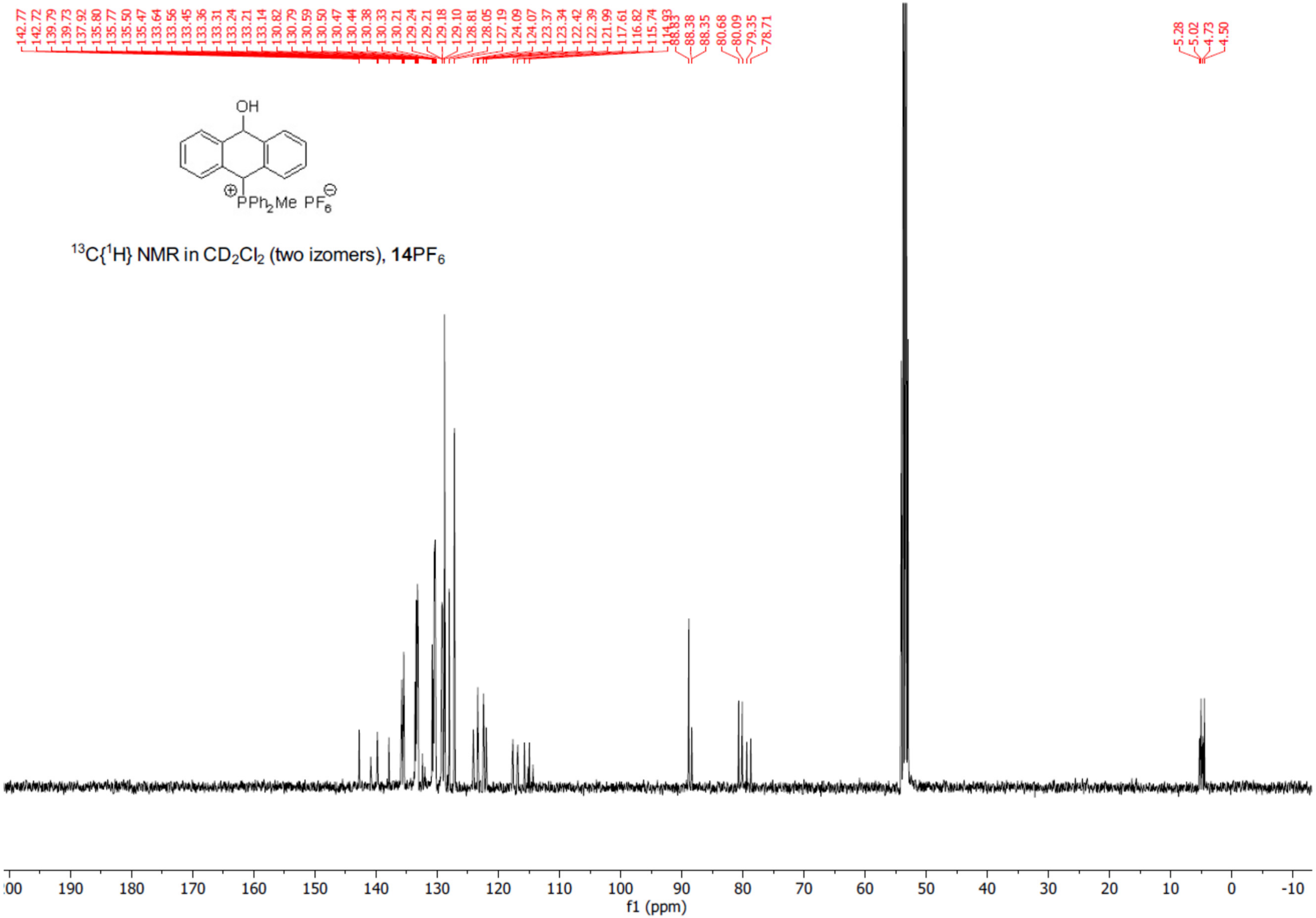

$^{13}C\{^1H\}$ NMR in CD_2Cl_2 (two izomers), **14**PF_6

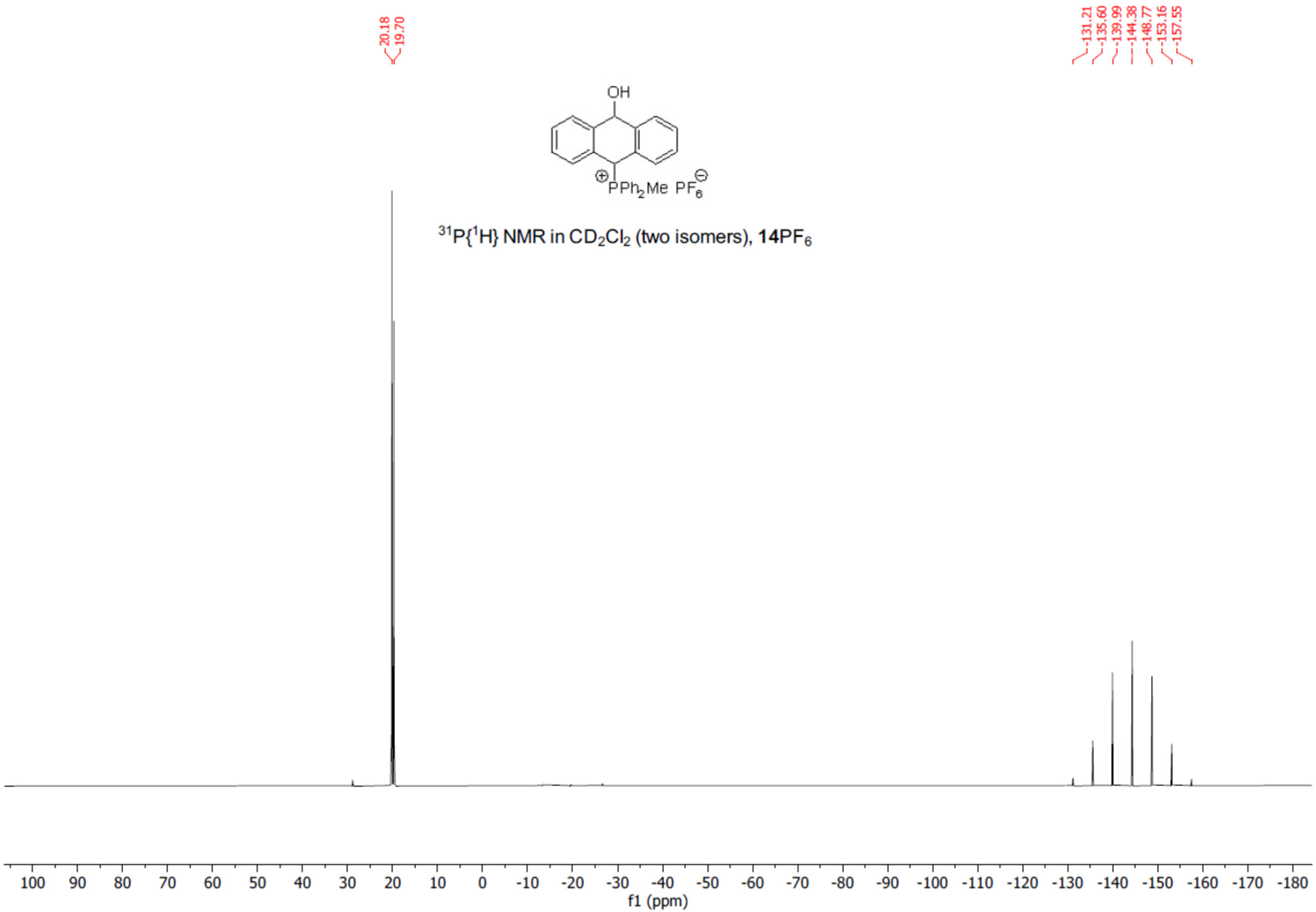

20.18
19.70
-131.21
-135.60
-139.99
-144.38
-148.77
-153.16
-157.55
OH
PPh2Me PF6
$^{31}P\{^1H\}$ NMR in CD_2Cl_2 (two isomers), **14**PF_6
100 90 80 70 60 50 40 30 20 10 0 -10 -20 -30 -40 -50 -60 -70 -80 -90 -100 -110 -120 -130 -140 -150 -160 -170 -180
f1 (ppm)

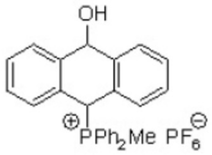

$^{19}F\{^{1}H\}$ NMR in CD_2Cl_2 (two isomers), **14**PF_6

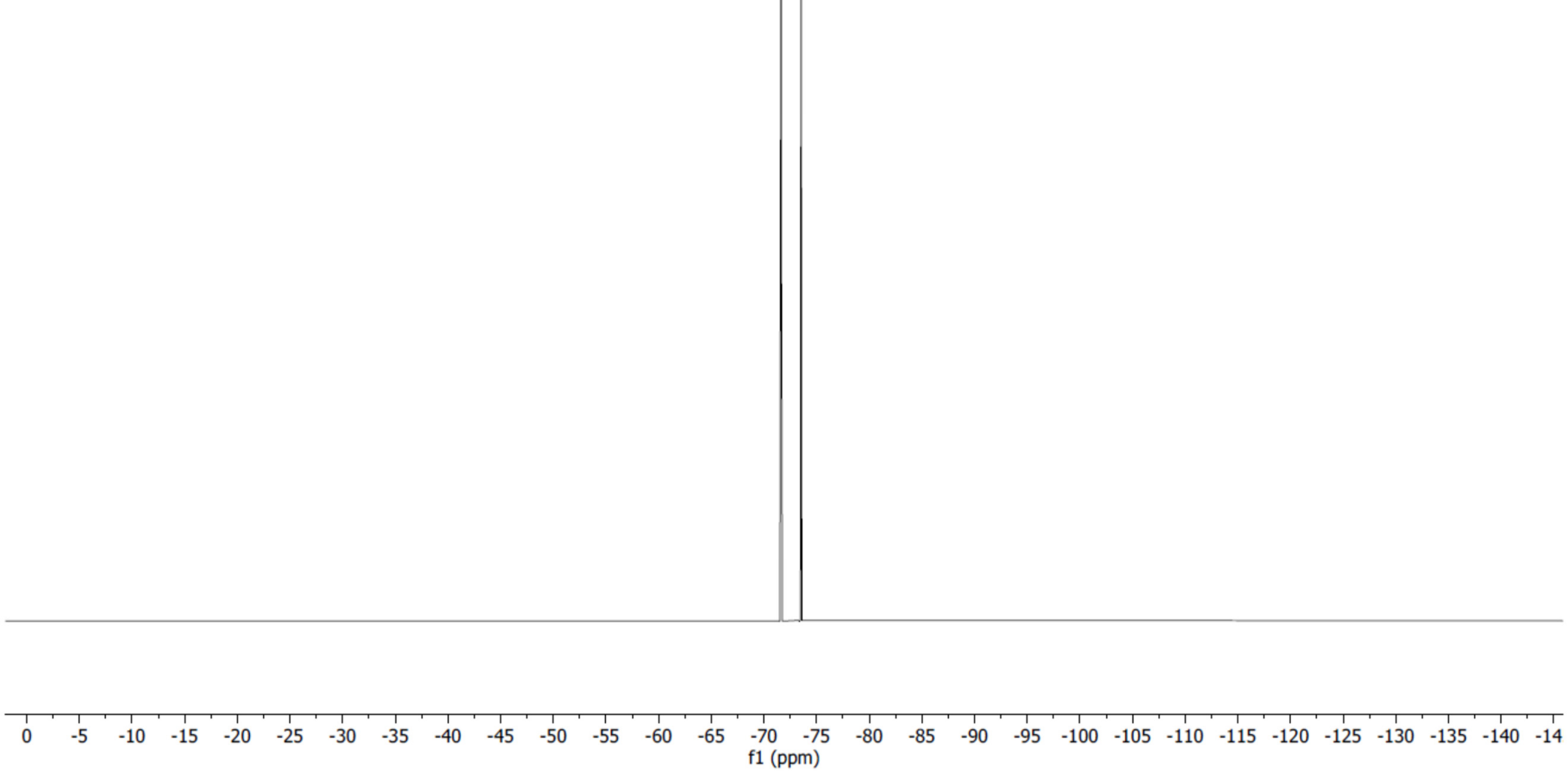

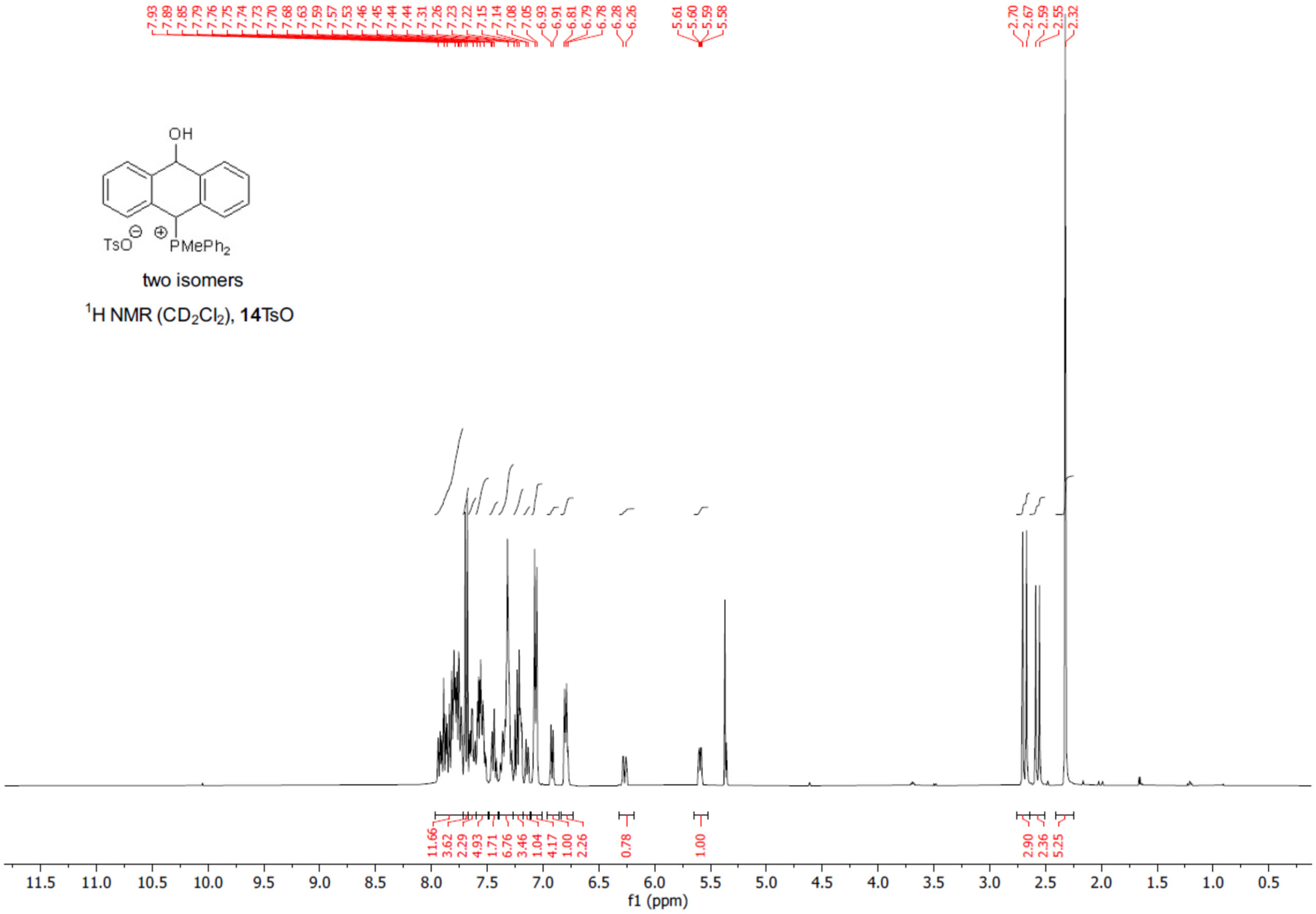

7.93 7.89 7.85 7.79 7.76 7.75 7.74 7.73 7.70 7.68 7.63 7.59 7.57 7.53 7.46 7.45 7.44 7.44 7.31 7.26 7.23 7.22 7.15 7.14 7.08 7.05 6.93 6.91 6.81 6.79 6.78 6.28 6.26
5.61 5.60 5.59 5.58
2.70 2.67 2.59 2.55 2.32
OH
TsO
PMePh2
two isomers
1H NMR (CD2Cl2), 14TsO
11.66 3.62 2.29 4.93 1.71 6.76 3.46 1.04 4.17 1.00 2.26
0.78
1.00
2.90 2.36 5.25
11.5 11.0 10.5 10.0 9.5 9.0 8.5 8.0 7.5 7.0 6.5 6.0 5.5 5.0 4.5 4.0 3.5 3.0 2.5 2.0 1.5 1.0 0.5
f1 (ppm)

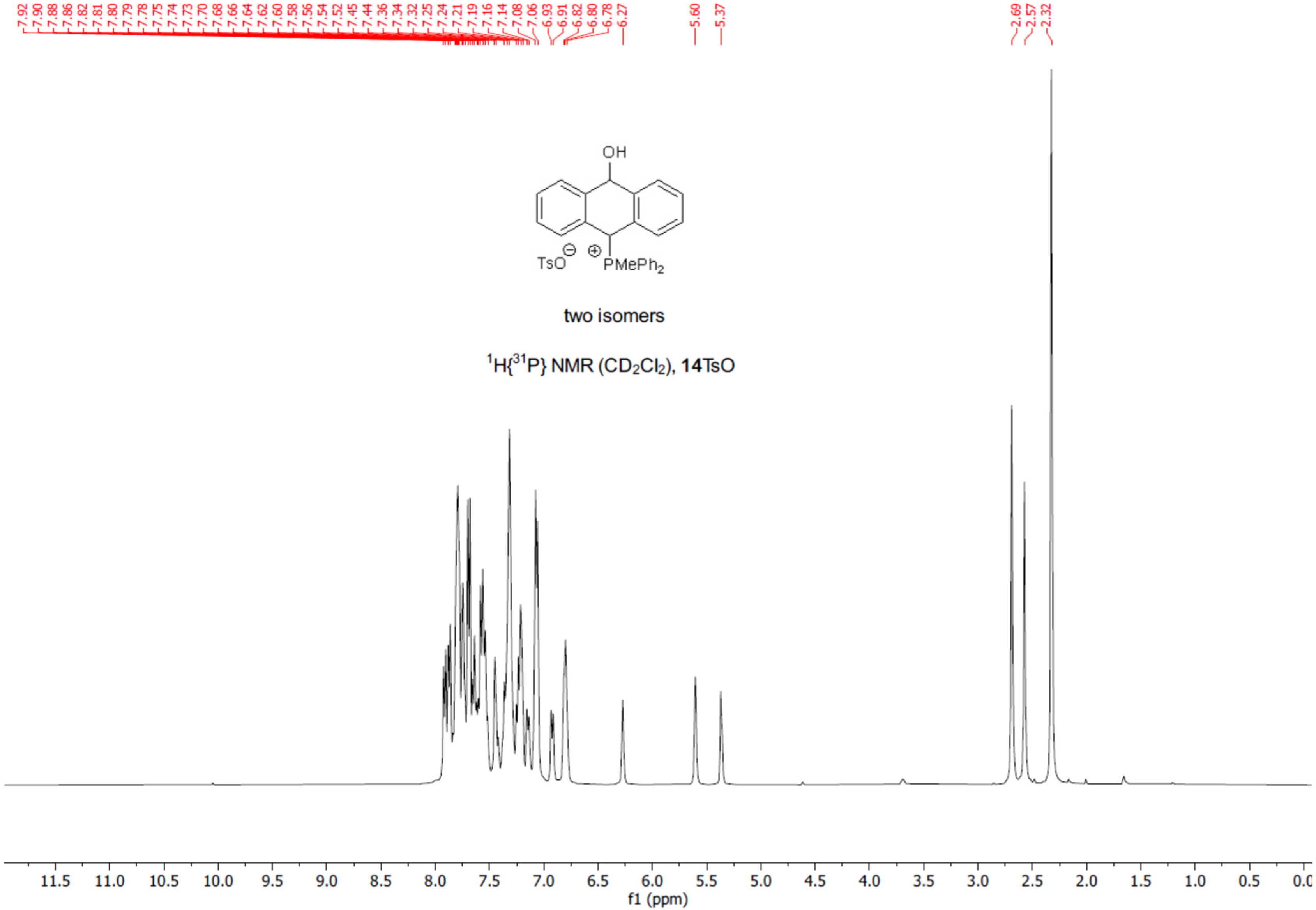
7.92 7.90 7.88 7.86 7.82 7.81 7.80 7.79 7.78 7.75 7.74 7.73 7.70 7.68 7.66 7.64 7.62 7.60 7.58 7.56 7.54 7.52 7.45 7.44 7.36 7.34 7.32 7.25 7.24 7.21 7.19 7.16 7.14 7.08 7.06 6.93 6.91 6.82 6.80 6.78 6.27
5.60 5.37
2.69 2.57 2.32
OH
TsO
PMePh2
two isomers
1H{31P} NMR (CD2Cl2), 14TsO
11.5 11.0 10.5 10.0 9.5 9.0 8.5 8.0 7.5 7.0 6.5 6.0 5.5 5.0 4.5 4.0 3.5 3.0 2.5 2.0 1.5 1.0 0.5 0.0
f1 (ppm)

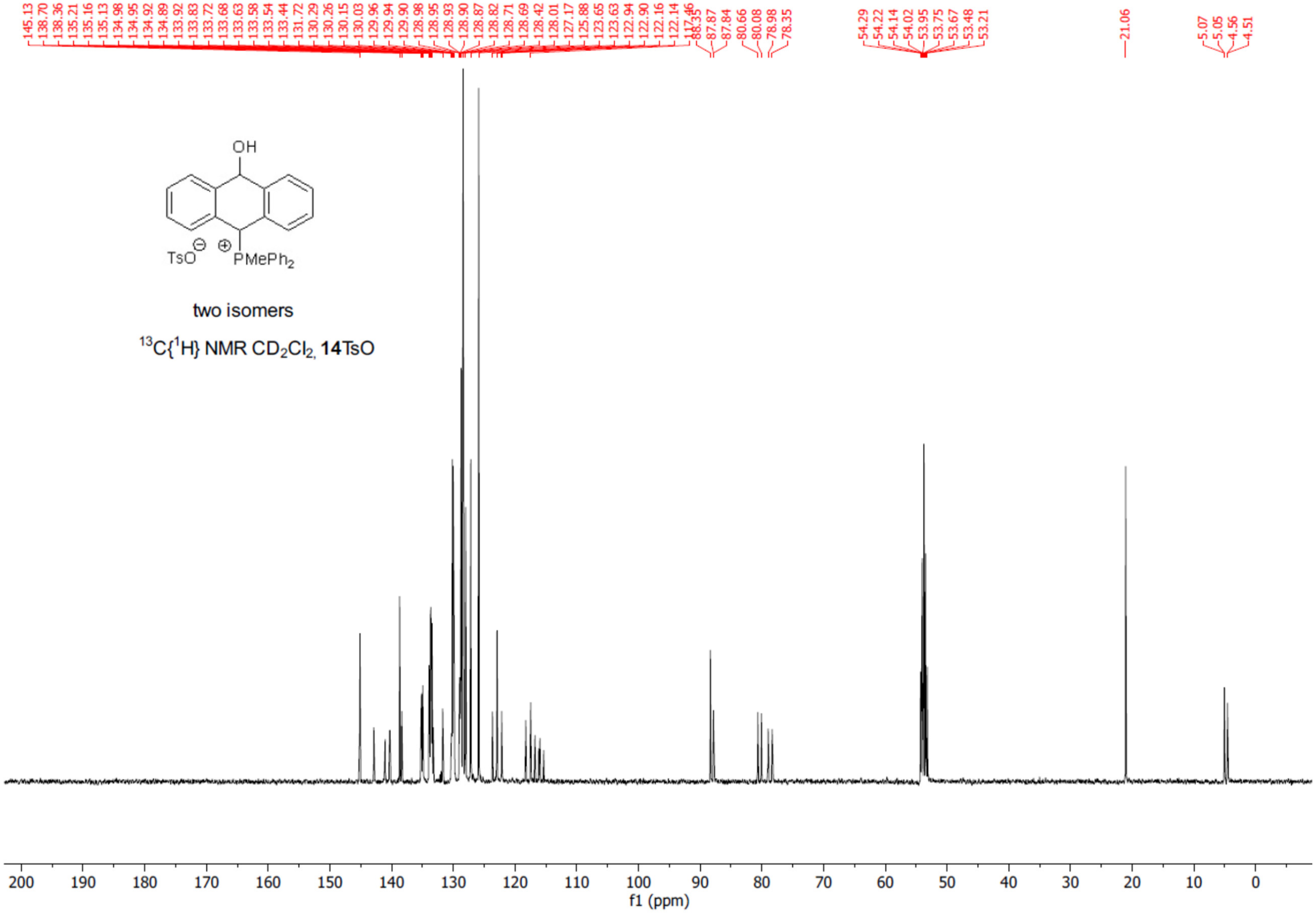
OH
TsO
PMePh2
two isomers
13C{1H} NMR CD2Cl2, 14TsO
145.13
138.70
138.36
135.21
135.16
135.13
134.98
134.95
134.92
134.89
133.92
133.83
133.72
133.68
133.63
133.58
133.54
133.44
131.72
130.29
130.26
130.15
130.03
129.96
129.94
129.90
128.98
128.95
128.93
128.90
128.87
128.82
128.71
128.69
128.42
128.01
127.17
125.88
123.65
123.63
122.94
122.90
122.16
122.14
117.46
88.35
87.87
87.84
80.66
80.08
78.98
78.35
54.29
54.22
54.14
54.02
53.95
53.75
53.67
53.48
53.21
21.06
5.07
5.05
4.56
4.51
f1 (ppm)

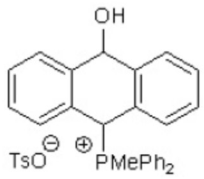

two isomers

$^{31}P\{^{1}H\}$ NMR (CD_2Cl_2), **14**TsO

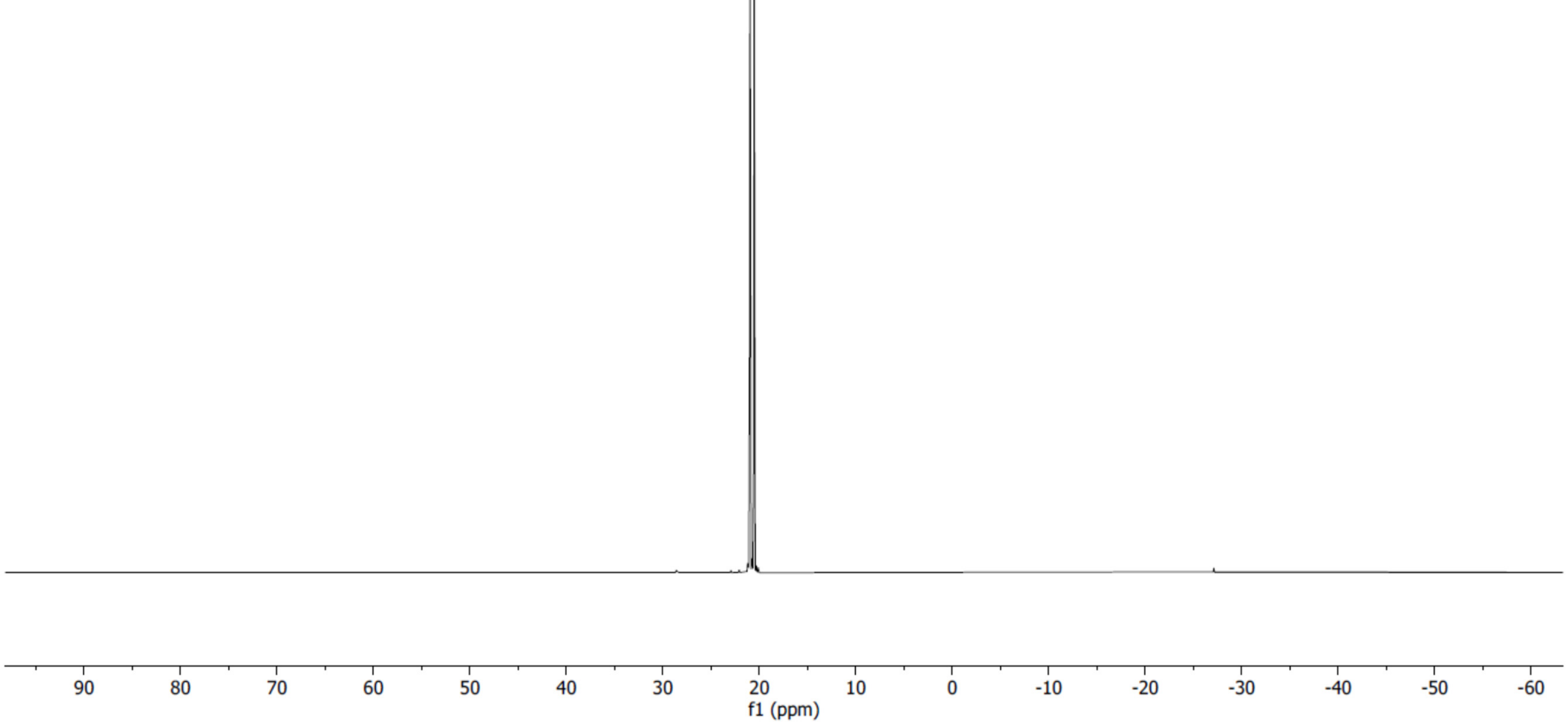

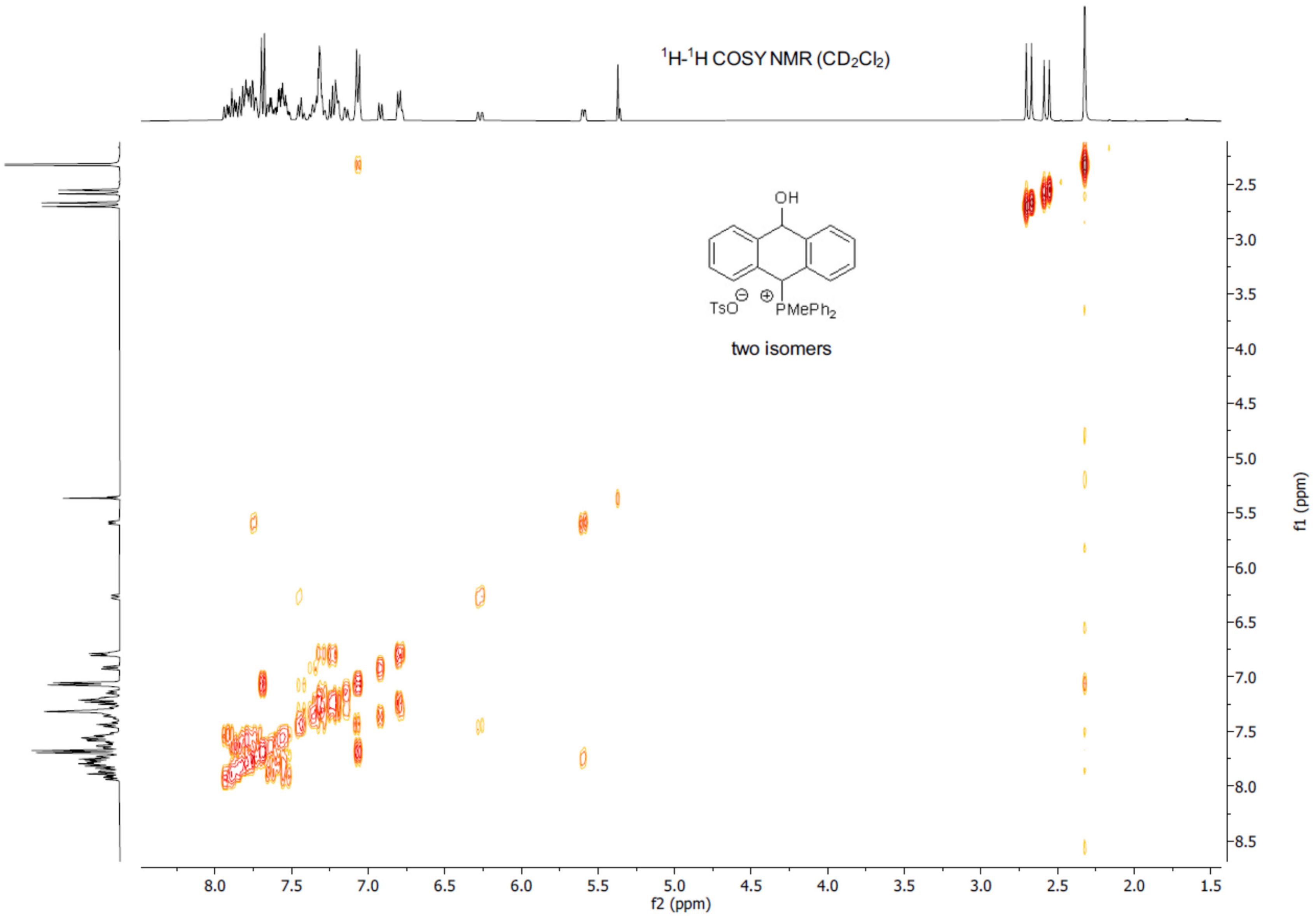

^{1}H-^{1}H COSY NMR (CD_2Cl_2)
OH
TsO
PMePh$_2$
two isomers
f1 (ppm)
f2 (ppm)

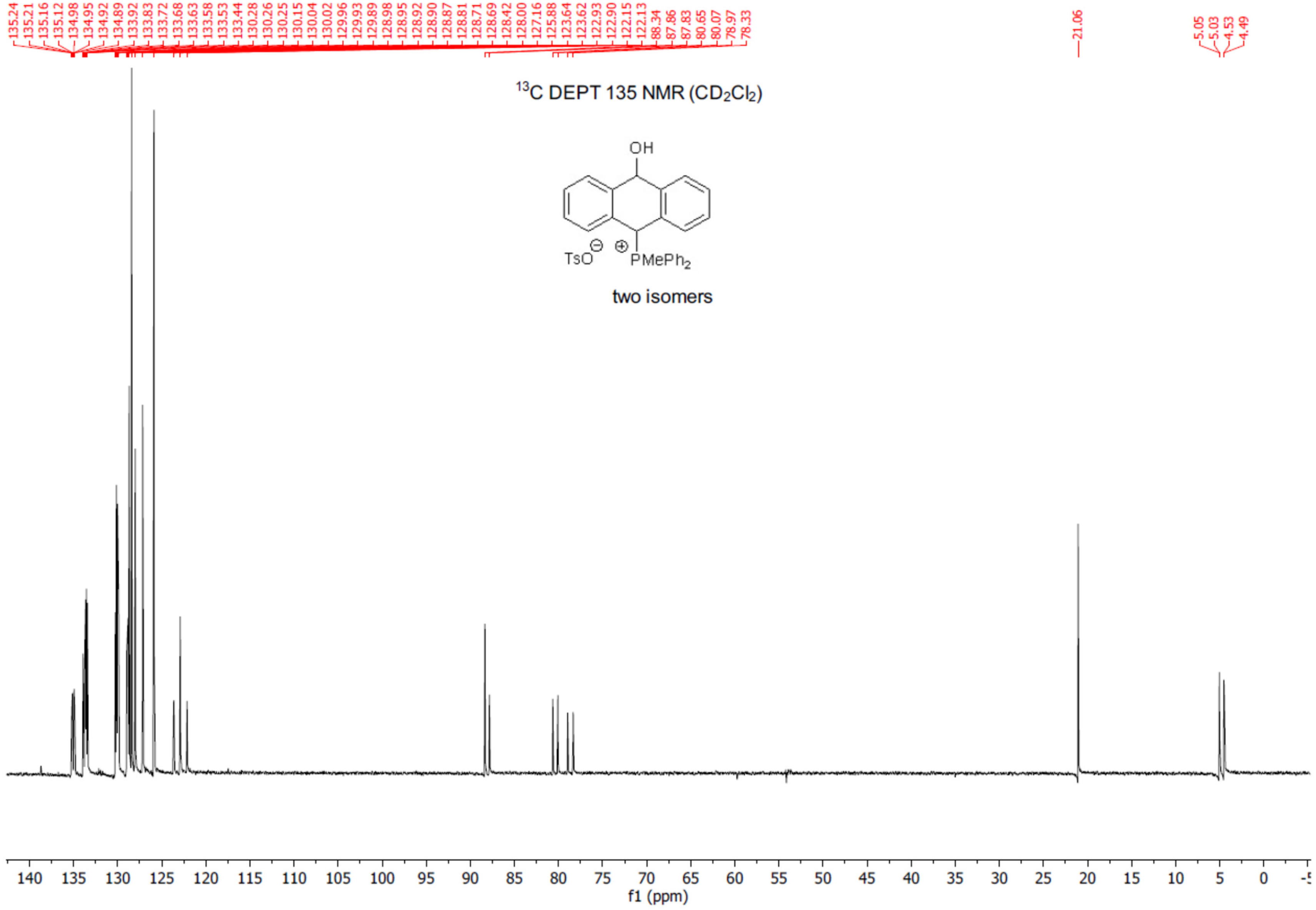

135.24
135.21
135.16
135.12
134.98
134.95
134.92
134.89
133.92
133.83
133.72
133.68
133.63
133.58
133.53
133.44
130.28
130.26
130.25
130.15
130.04
130.02
129.96
129.93
129.89
128.98
128.95
128.92
128.90
128.87
128.81
128.71
128.69
128.42
128.00
127.16
125.88
123.64
123.62
122.93
122.90
122.15
122.13
88.34
87.86
87.83
80.65
80.07
78.97
78.33
21.06
5.05
5.03
4.53
4.49
13C DEPT 135 NMR (CD2Cl2)
OH
TsO
PMePh2
two isomers
140 135 130 125 120 115 110 105 100 95 90 85 80 75 70 65 60 55 50 45 40 35 30 25 20 15 10 5 0
f1 (ppm)

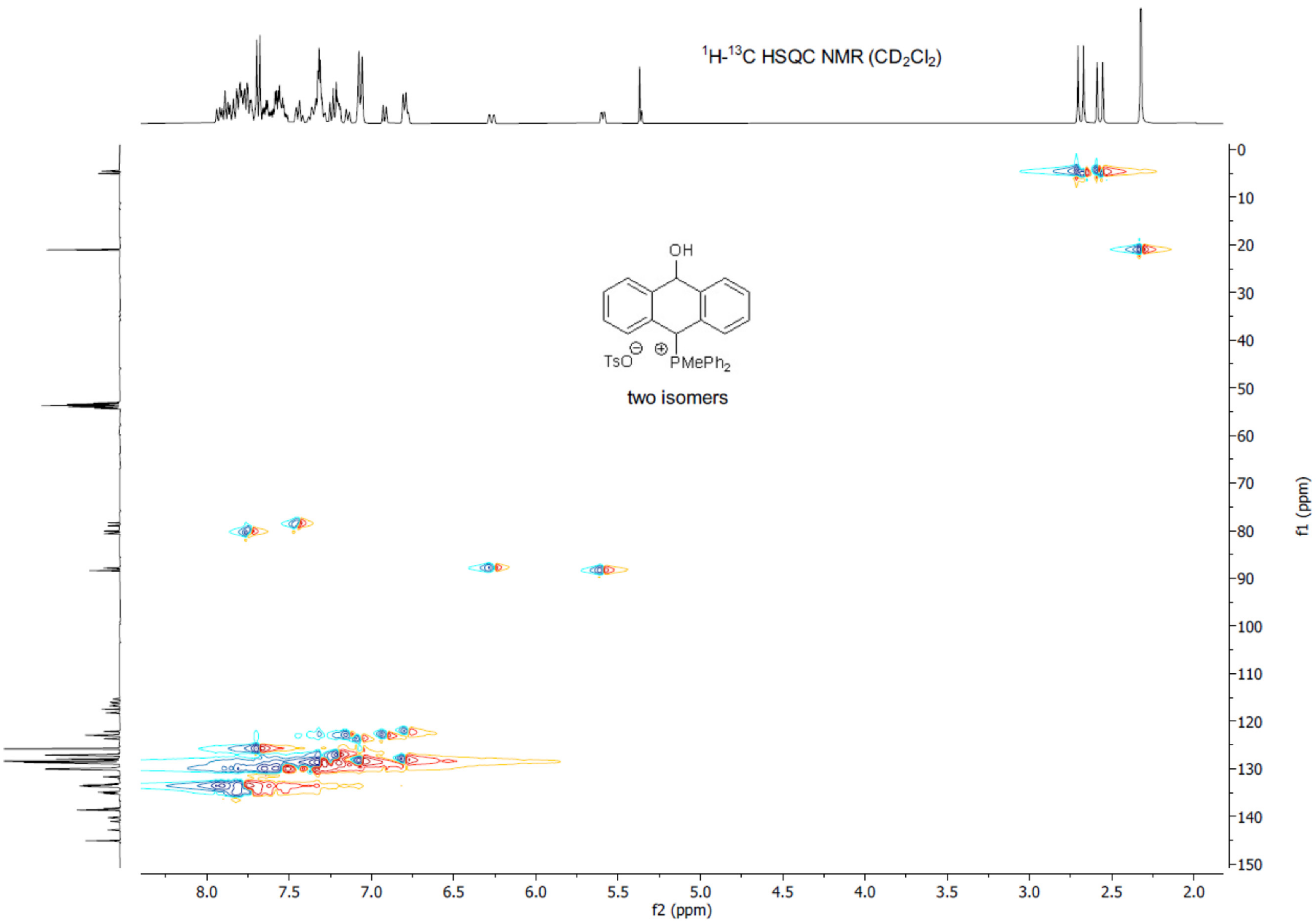

^{1}H-^{13}C HSQC NMR (CD_2Cl_2)
OH
TsO
PMePh$_2$
two isomers
f1 (ppm)
f2 (ppm)
0
10
20
30
40
50
60
70
80
90
100
110
120
130
140
150
8.0
7.5
7.0
6.5
6.0
5.5
5.0
4.5
4.0
3.5
3.0
2.5
2.0

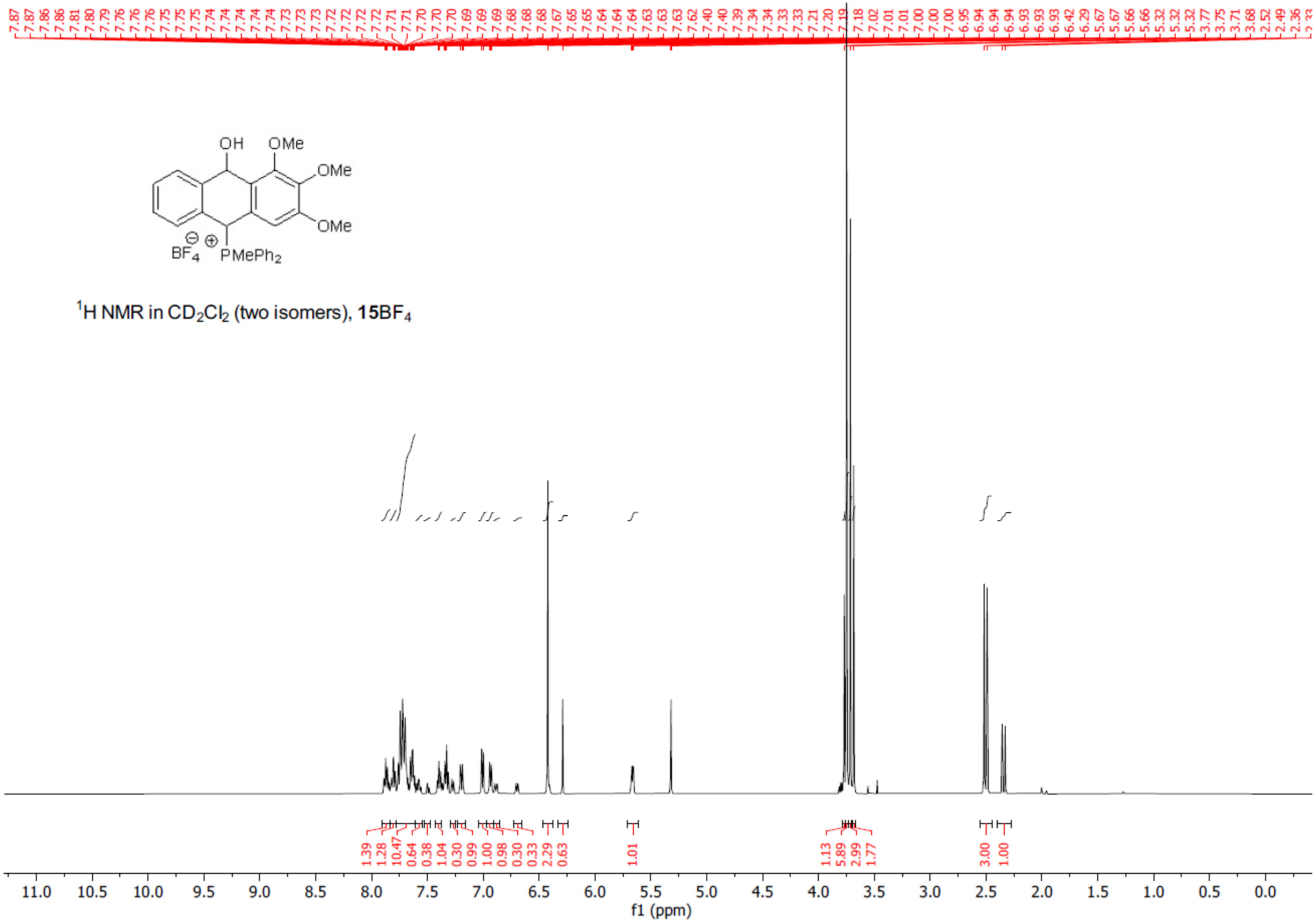

^{1}H NMR in CD_2Cl_2 (two isomers), **15**BF_4

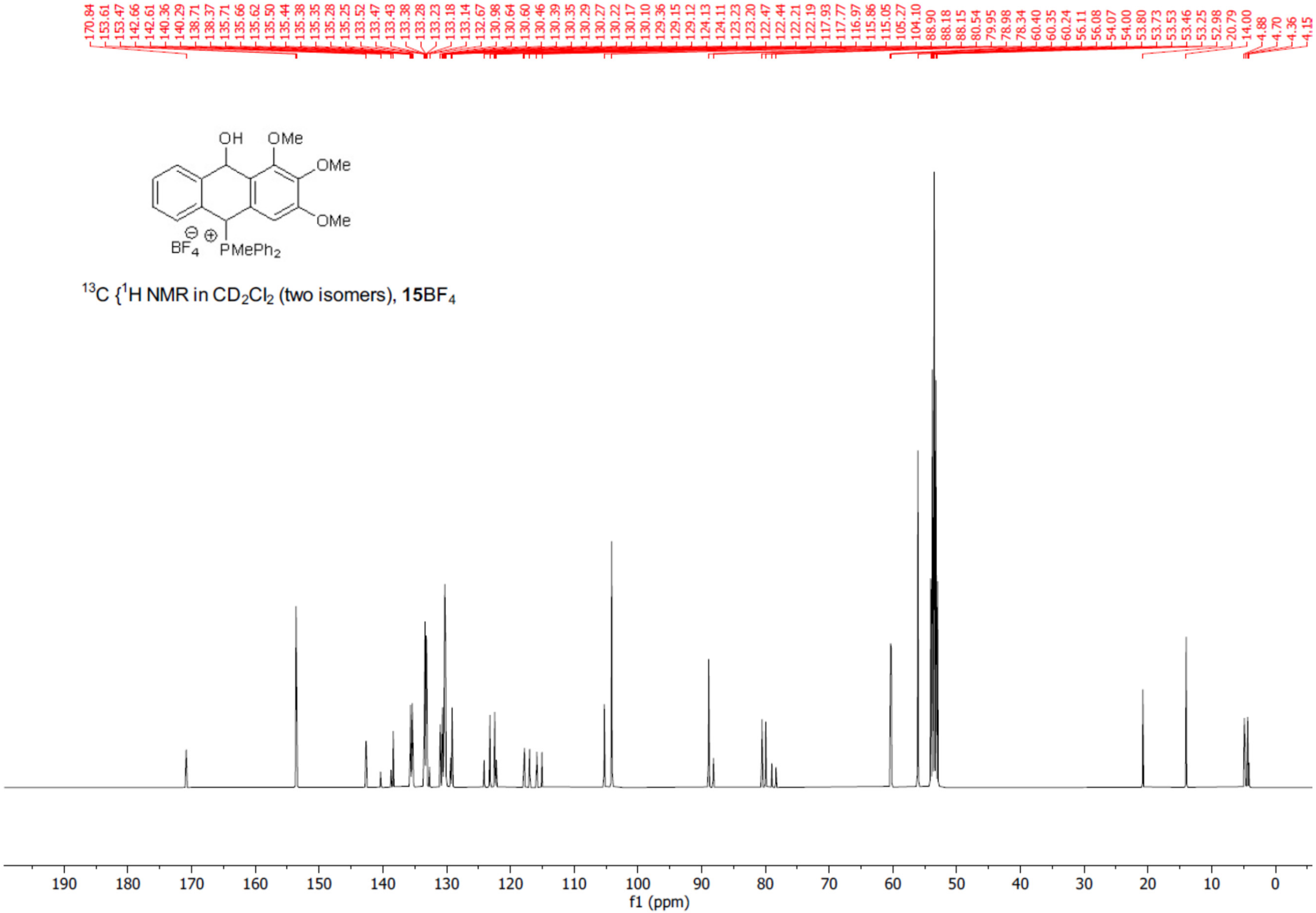

^{13}C {^1H NMR in CD_2Cl_2 (two isomers), **15**BF_4

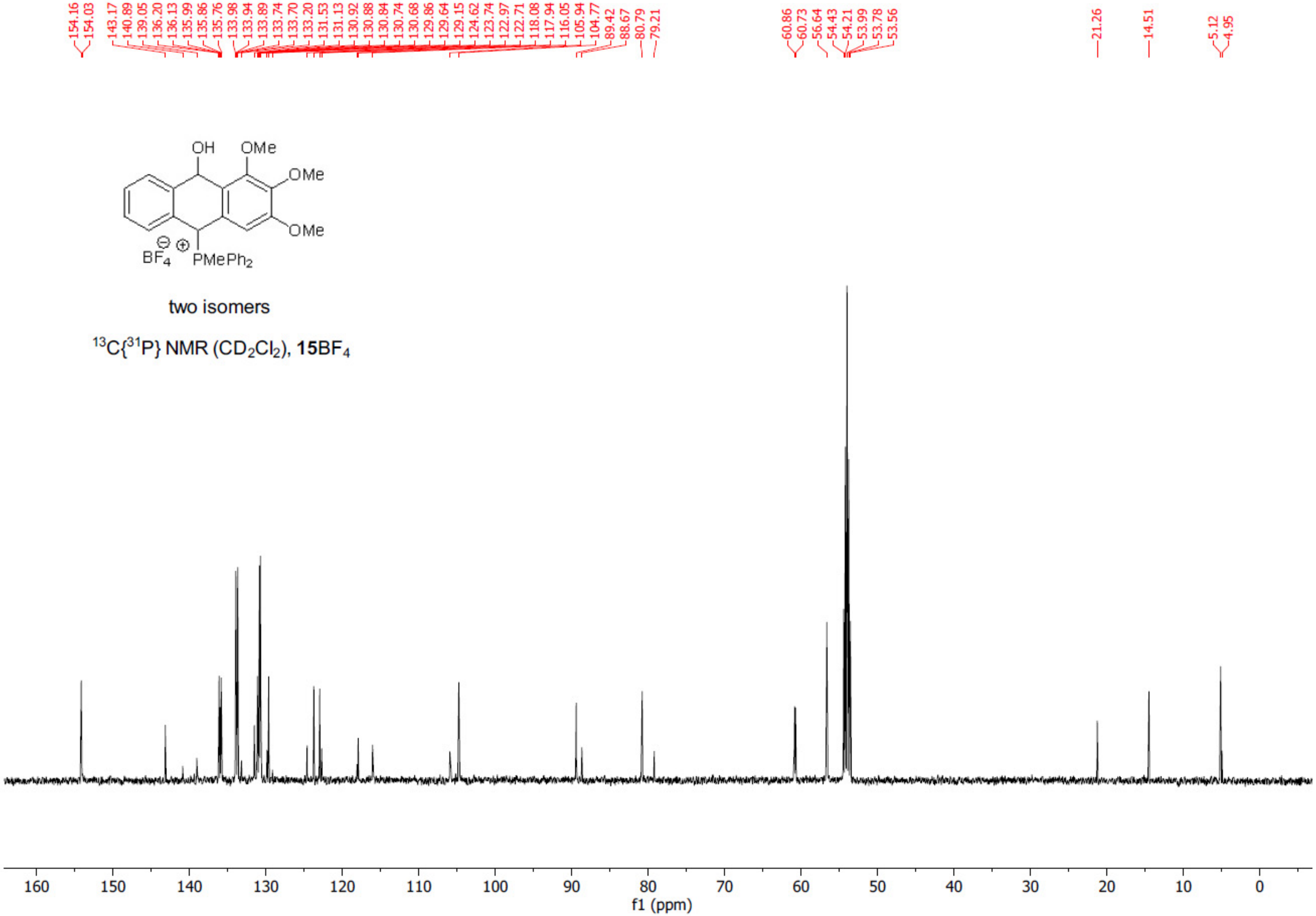
154.16
154.03
143.17
140.89
139.05
136.20
136.13
135.99
135.86
135.76
133.98
133.94
133.89
133.74
133.70
133.20
131.53
131.13
130.92
130.88
130.84
130.74
130.68
129.86
129.64
129.15
124.62
123.74
122.97
122.71
118.08
117.94
116.05
105.94
104.77
89.42
88.67
80.79
79.21
60.86
60.73
56.64
54.43
54.21
53.99
53.78
53.56
21.26
14.51
5.12
4.95
OH
OMe
OMe
OMe
BF4
PMePh2
two isomers
13C{31P} NMR (CD2Cl2), 15BF4
160
150
140
130
120
110
100
90
80
70
60
50
40
30
20
10
0
f1 (ppm)

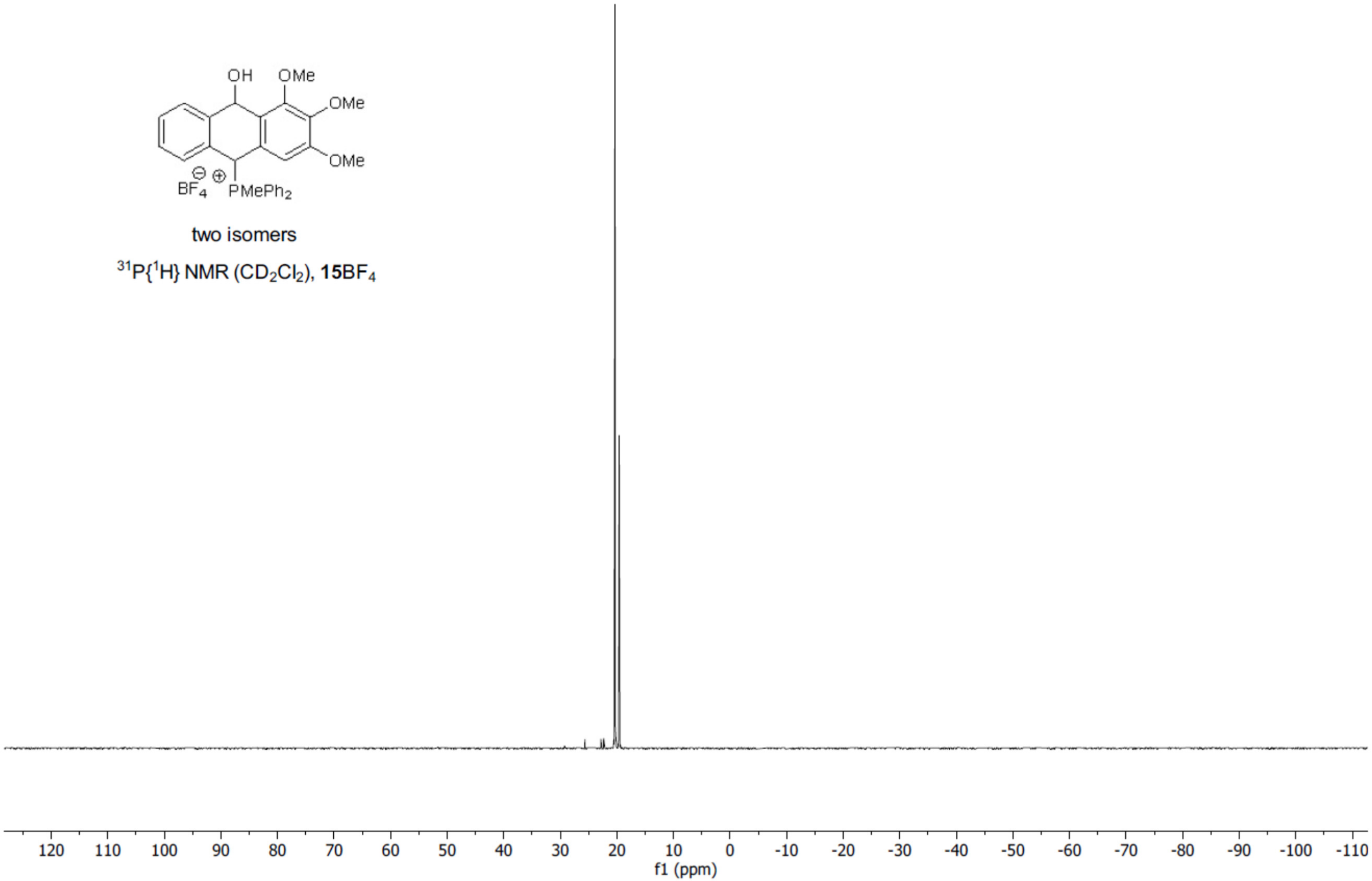

two isomers

$^{31}P\{^1H\}$ NMR (CD_2Cl_2), **15**BF_4

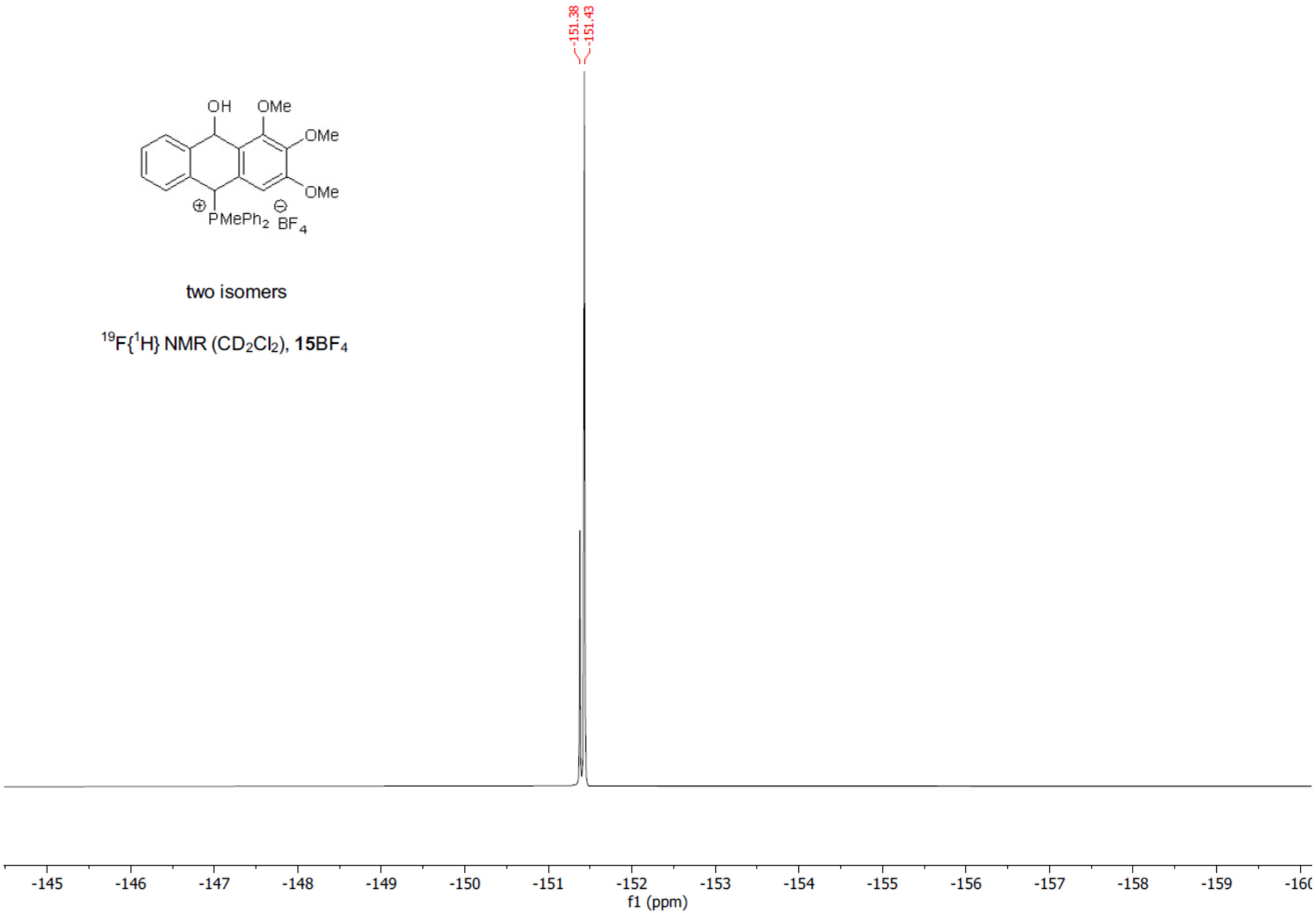

two isomers

$^{19}F\{^{1}H\}$ NMR (CD_2Cl_2), **15**BF_4

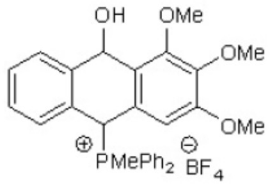

^{13}C-DEPT-135 NMR (CD_2Cl_2), **15**BF_4

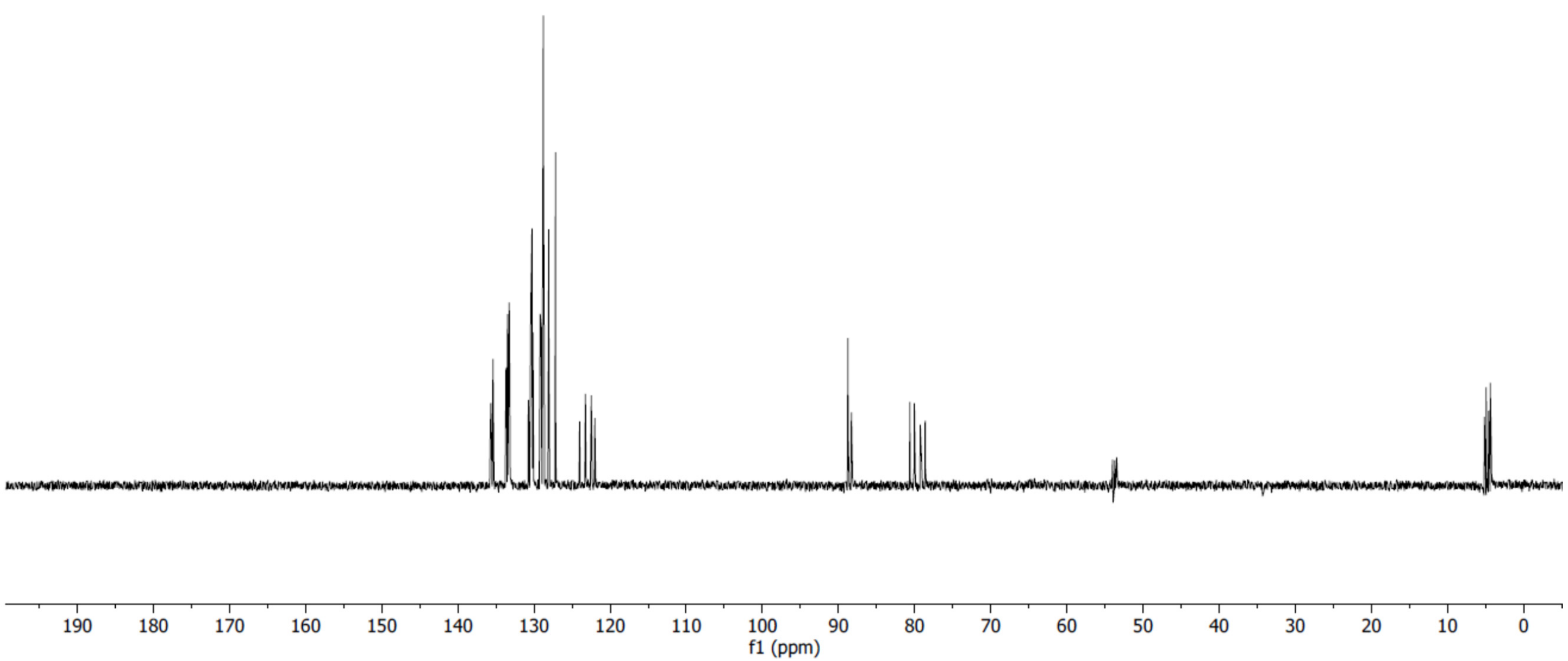

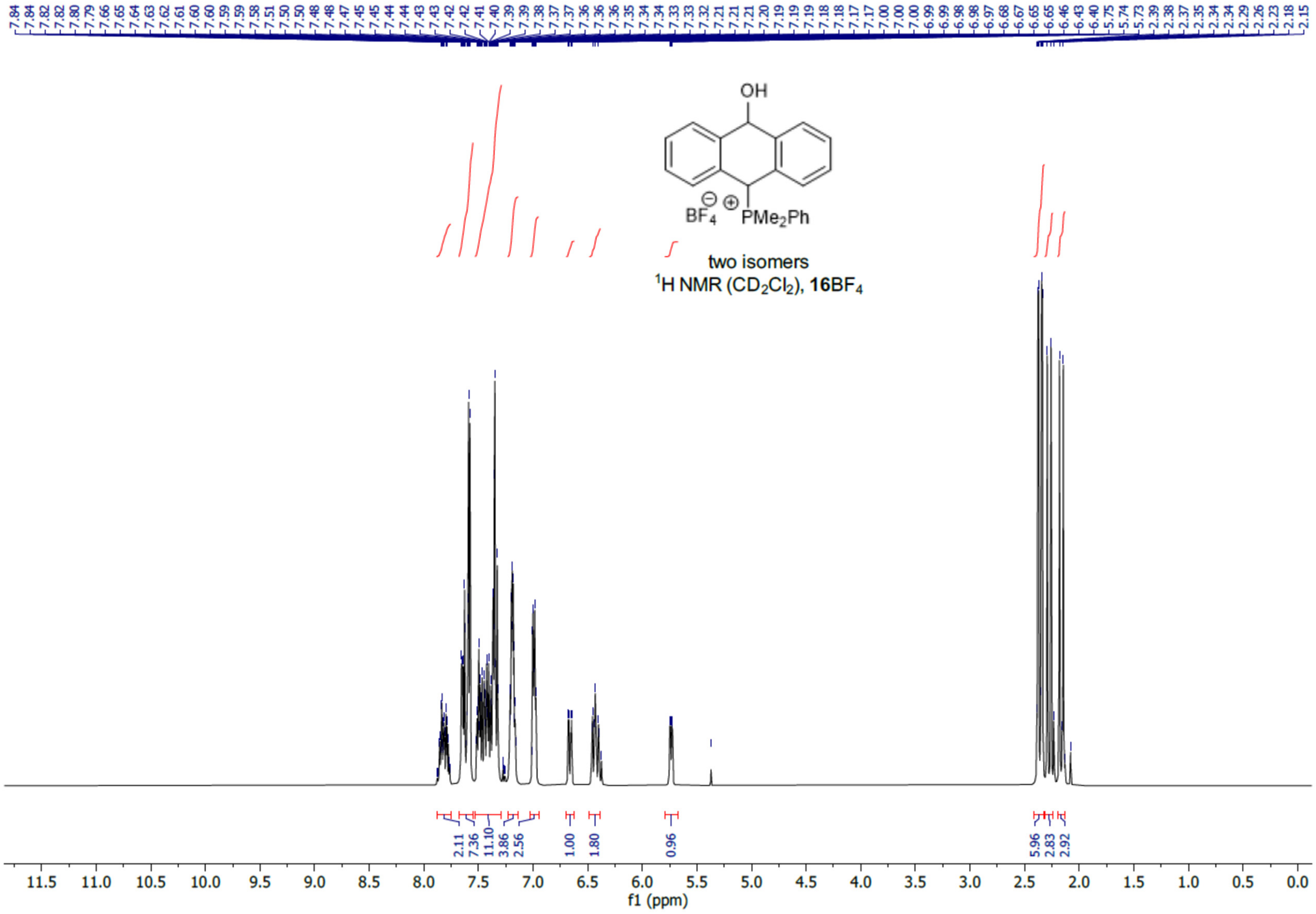
OH
BF4
PMe2Ph
two isomers
1H NMR (CD2Cl2), 16BF4
f1 (ppm)

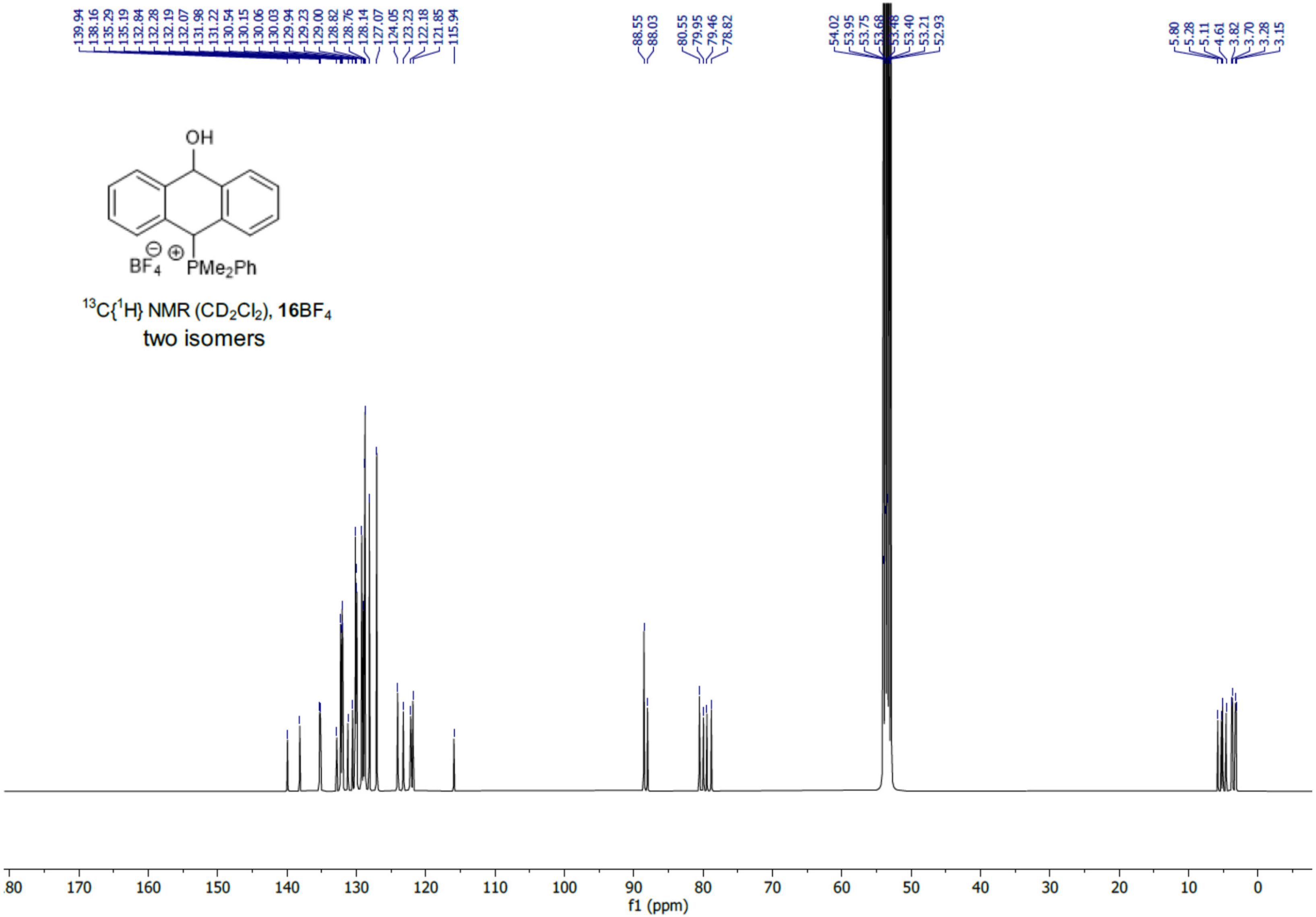
139.94
138.16
135.29
135.19
132.84
132.28
132.19
132.07
131.98
131.22
130.54
130.15
130.06
130.03
129.94
129.23
129.00
128.82
128.76
128.14
127.07
124.05
123.23
122.18
121.85
115.94
88.55
88.03
80.55
79.95
79.46
78.82
54.02
53.95
53.75
53.68
53.48
53.40
53.21
52.93
5.80
5.28
5.11
4.61
3.82
3.70
3.28
3.15
OH
BF4
PMe2Ph
13C{1H} NMR (CD2Cl2), 16BF4
two isomers
f1 (ppm)

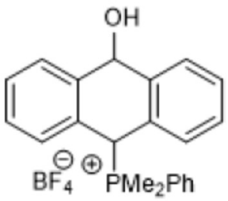

$^{31}P\{^{1}H\}$ NMR (CD_2Cl_2), **16**BF_4

two isomers (cis, trans)

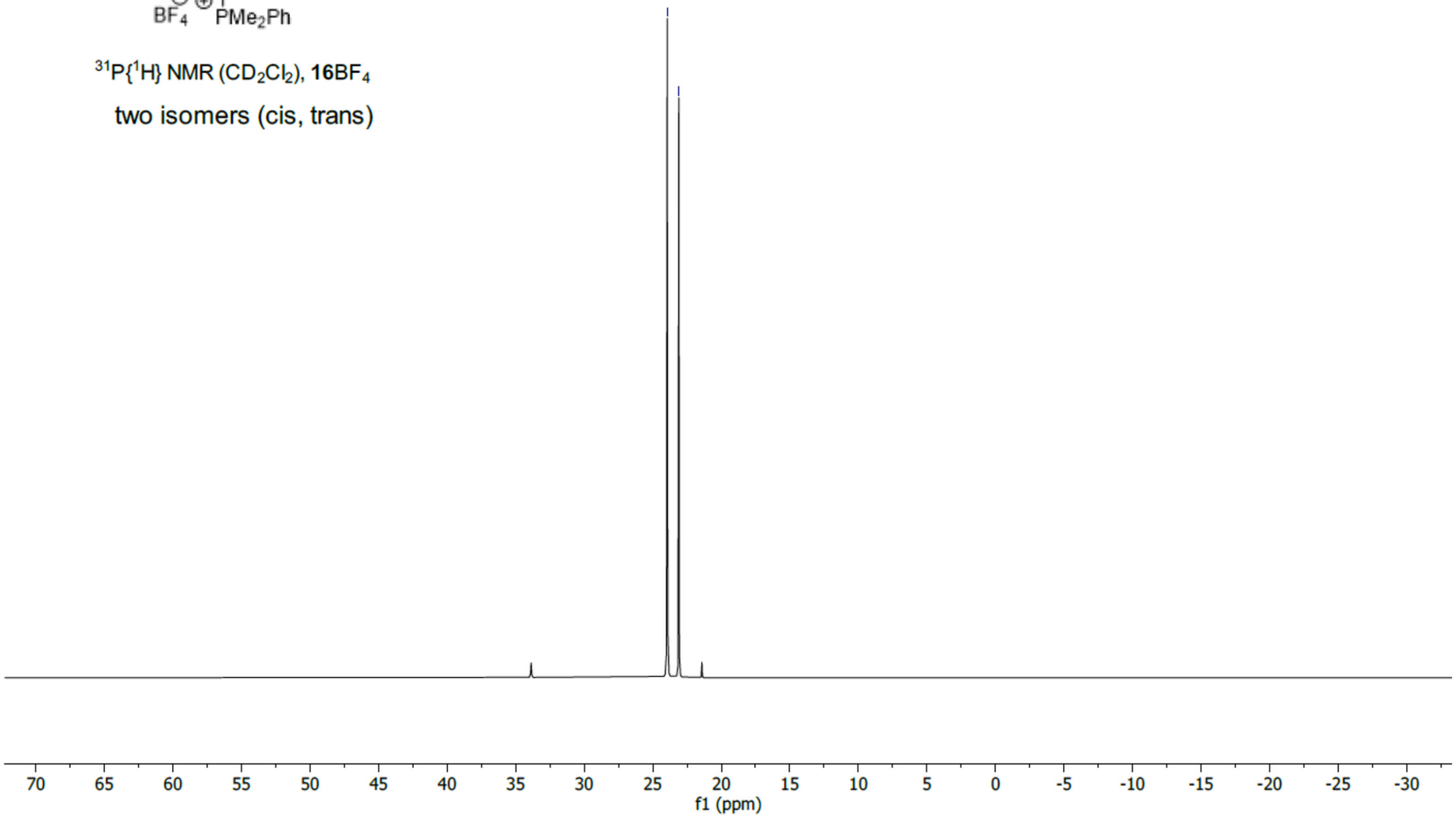

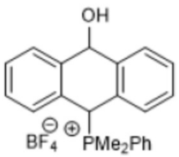

$^{11}B\{^{1}H\}$ NMR (CD_2Cl_2), **16**BF_4

two isomers (cis, trans)

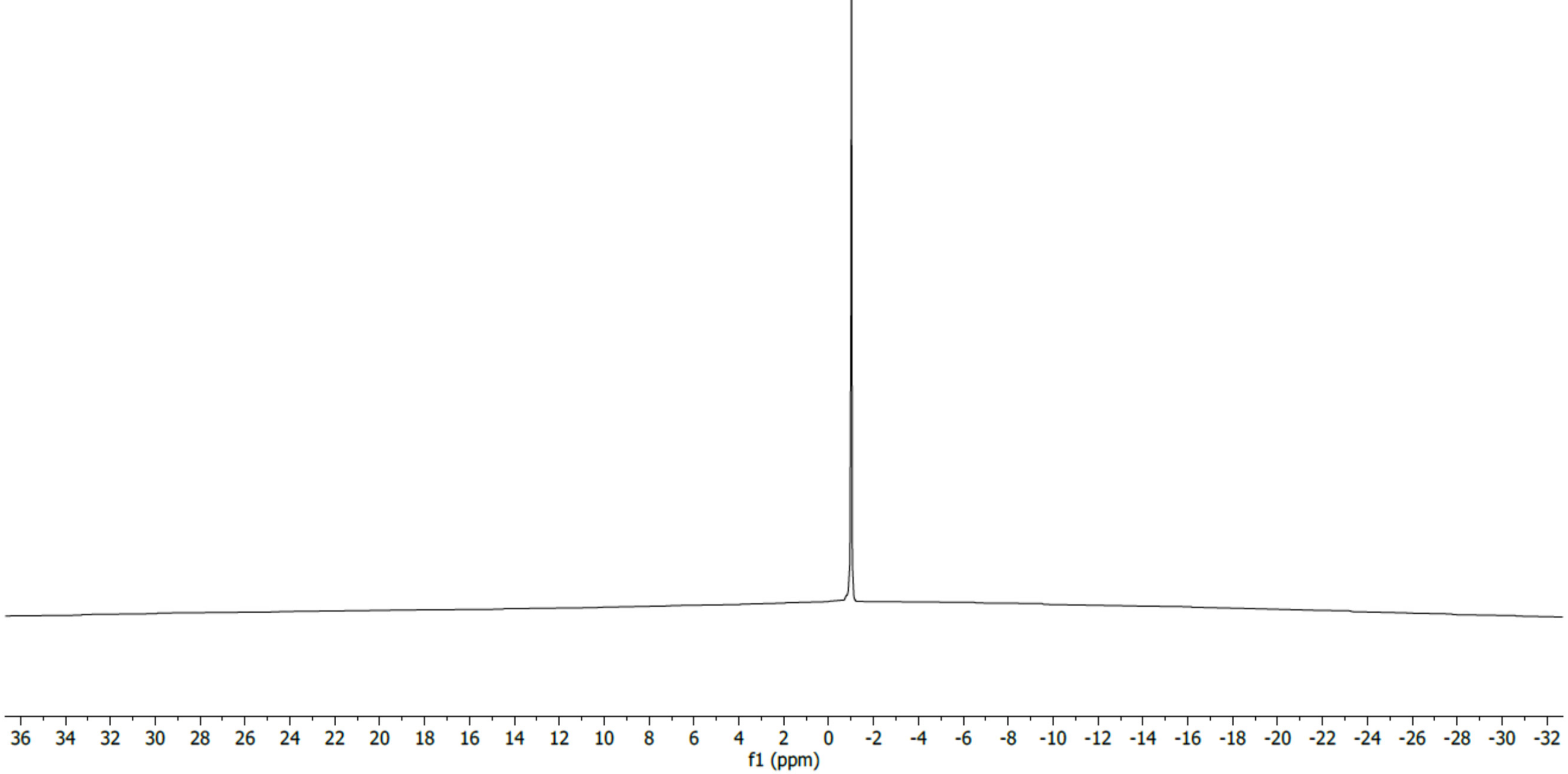

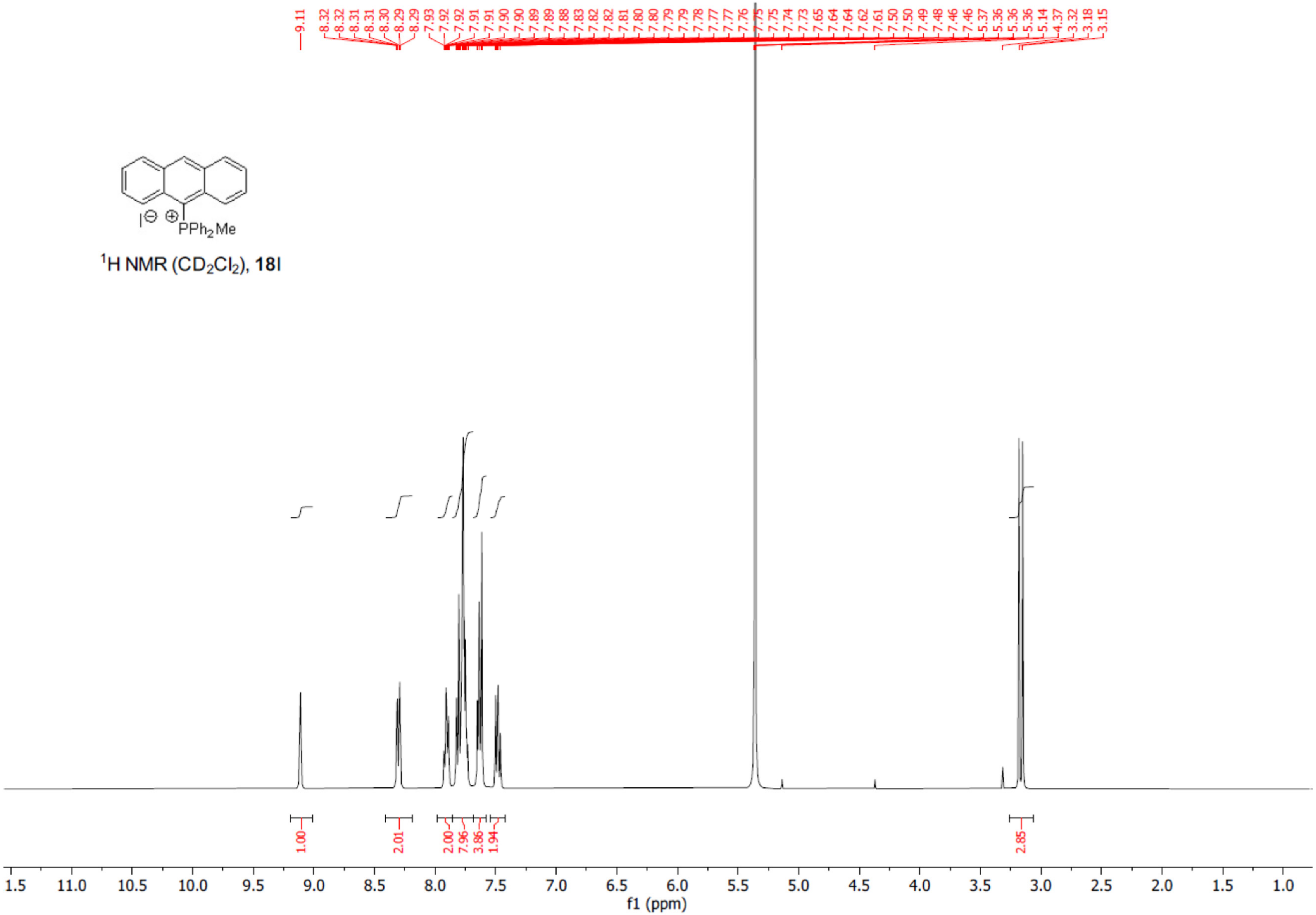
PPh2Me
1H NMR (CD2Cl2), 18I
f1 (ppm)

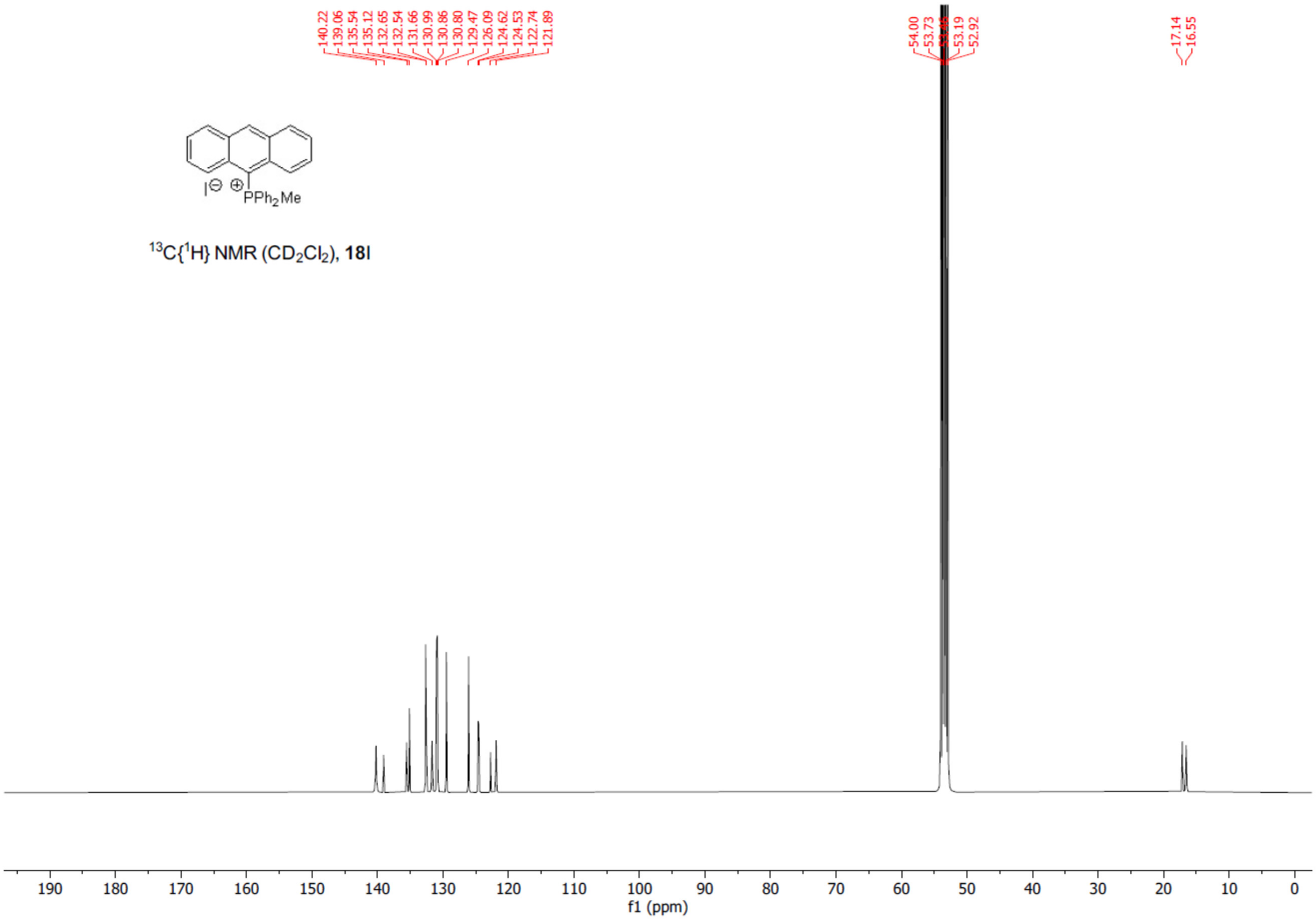

$^{13}C\{^{1}H\}$ NMR (CD_2Cl_2), **18I**

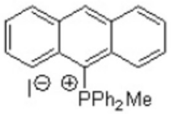

$^{31}P\{^{1}H\}$ NMR (CD_2Cl_2), **18I**

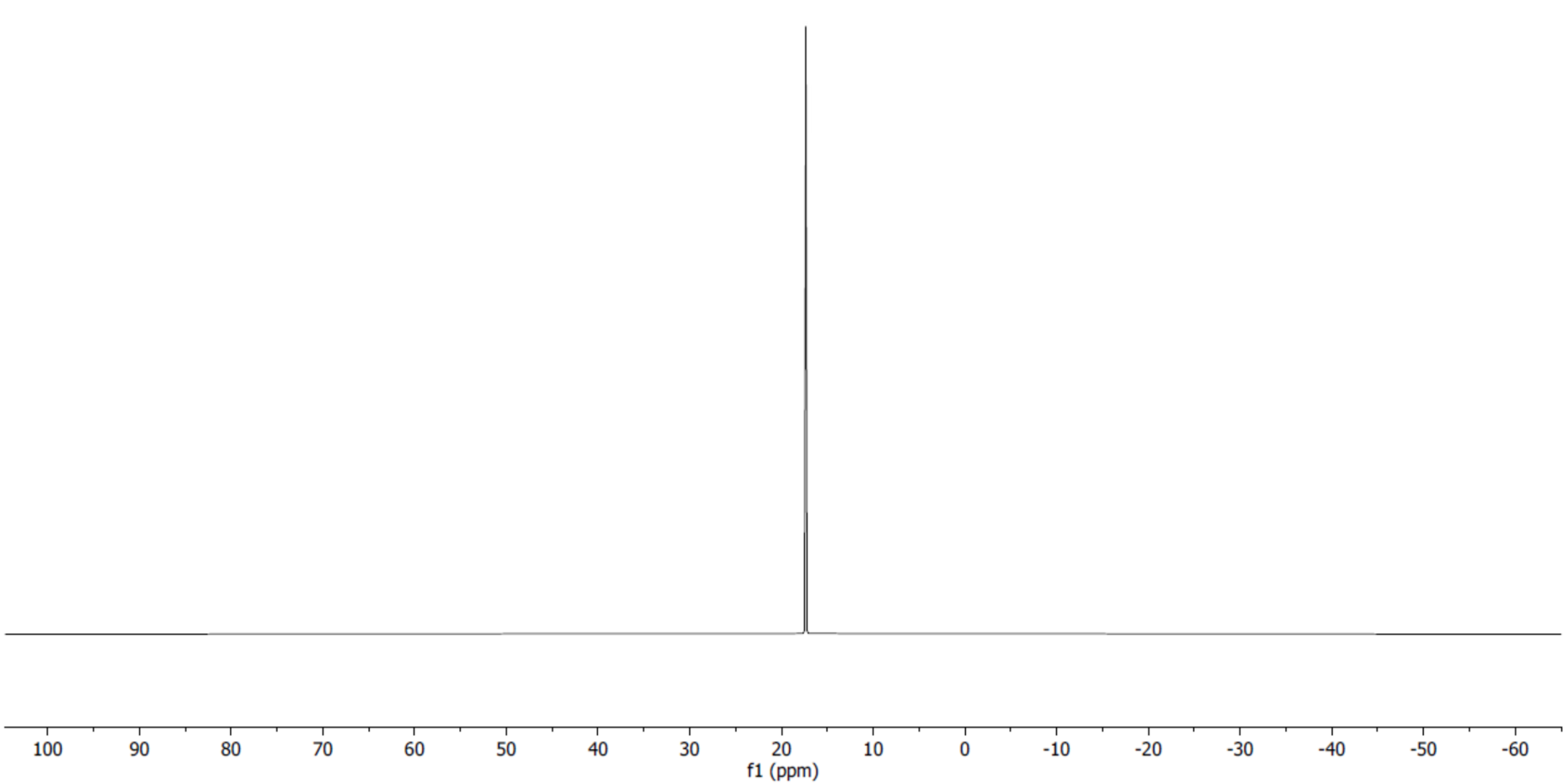

FT IR Spectra

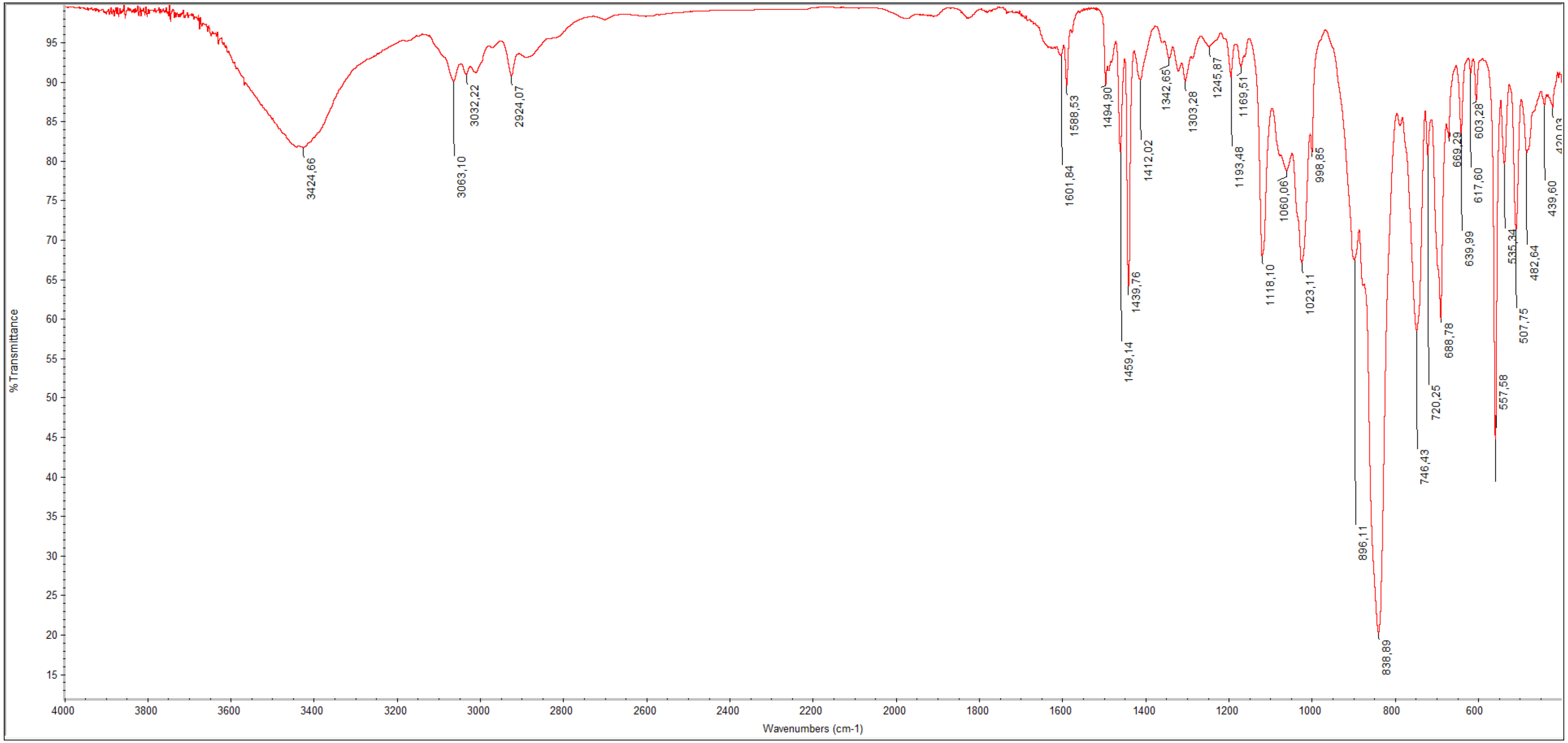

FT-IR (KBr) for **14**PF_6 (isomer *cis* and *trans*)

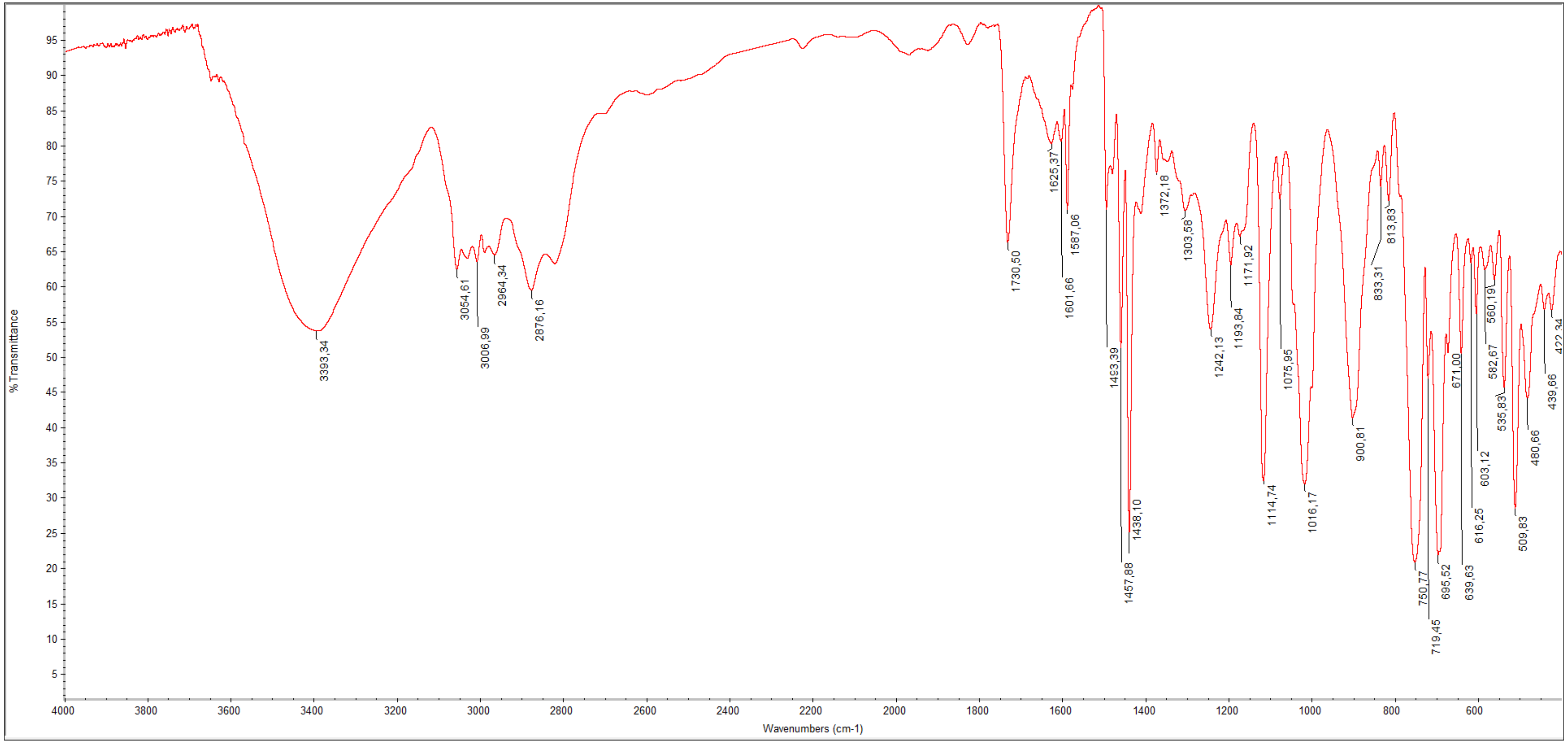

FT-IR (KBr) for **14**Cl (isomer *cis* and *trans*)

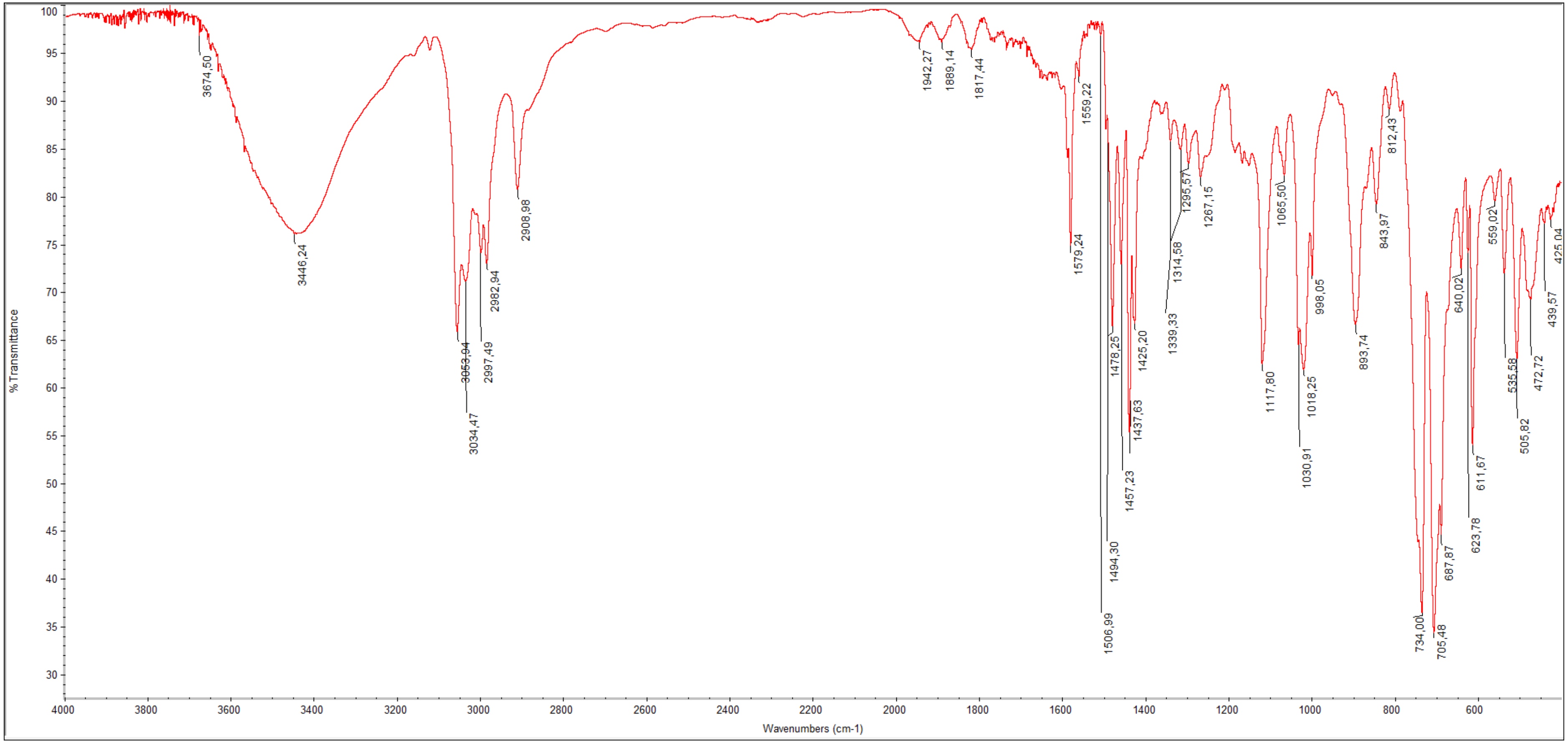

FT-IR (KBr) for **14**BPh4 (isomer *cis* and *trans*)
